# Formation of a C–C double bond from two aliphatic carbons. Multiple C–H activations in an iridium pincer complex†
†Electronic supplementary information (ESI) available: Experimental details, characterization data, Cartesian coordinates, additional graphs and computational details. CCDC 1024127–1024129. For ESI and crystallographic data in CIF or other electronic format see DOI: 10.1039/c4sc03839h



**DOI:** 10.1039/c4sc03839h

**Published:** 2015-01-26

**Authors:** Alexey V. Polukeev, Rocío Marcos, Mårten S. G. Ahlquist, Ola F. Wendt

**Affiliations:** a Centre for Analysis and Synthesis , Department of Chemistry , Lund University , PO Box 124 , 22100 Lund , Sweden . Email: ola.wendt@chem.lu.se; b Division of Theoretical Chemistry & Biology , School of Biotechnology , KTH Royal Institute of Technology , SE-106 91 Stockholm , Sweden

## Abstract

The search for novel, atom-economic methods for the formation of C–C bonds is of crucial importance in synthetic chemistry. Especially attractive are reactions where C–C bonds are formed through C–H activation, but the coupling of unactivated, alkane-type C_sp^3^_–H bonds remains an unsolved challenge. Here, we report iridium-mediated intramolecular coupling reactions involving up to four unactivated C_sp^3^_–H bonds to give carbon–carbon double bonds under the extrusion of dihydrogen. The reaction described herein is completely reversible and the direction can be controlled by altering the reaction conditions. With a hydrogen acceptor present a C–C double bond is formed, while reacting under dihydrogen pressure leads to the reverse process, with some of the steps representing net C_sp^3^_–C_sp^3^_ bond cleavage. Mechanistic investigations revealed a conceptually-novel overall reactivity pattern where insertion or deinsertion of an Ir carbene moiety, formed *via* double C–H activation, into an Ir–C bond is responsible for the key C–C bond formation and cleavage steps.

## Abstract 2

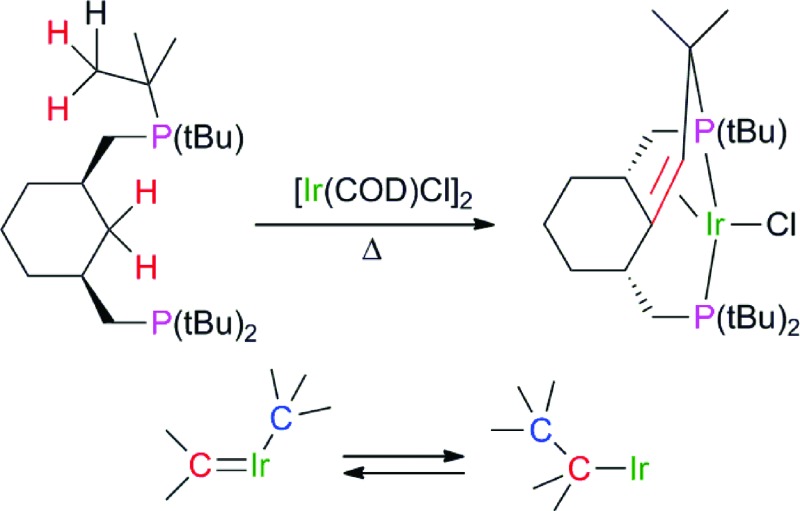
Iridium can mediate a reversible intramolecular coupling reaction involving up to four unactivated C_sp^3^_–H bonds, to give a carbon–carbon double bond.

## Introduction

The selective activation of C–H bonds by transition-metal complexes is one of the main goals in organometallic chemistry, as it may allow for efficient, low-waste methods for the functionalization of various organic molecules, not the least of cheap, but relatively inert alkanes.^1^ In particular, cross-coupling reactions where a C–C bond is formed *via* activation of C–H bonds of one^2^ or both^2d,3^ coupling partners have been intensively studied in recent years as a potential alternative to the traditional palladium-catalyzed couplings, where pre-functionalization of both substrates is required.^4^ Numerous methods have been developed for the connection of C_sp_–H, C_sp^2^_–H and C_sp^3^_–H bonds with each other,^2d,3^ with coupling of two C_sp^3^_–H bonds being the most challenging.^3*d*^ However, in the latter case protocols are rather substrate-specific and the scope is limited to substrates with activated C_sp^3^_–H bonds; strong oxidants like peroxides are used to drive the reaction.^3*d*^ Typically, one coupling partner bears a heteroatom adjacent to the C_sp^3^_–H bond, which facilitates oxidation and stabilizes the resulting electrophilic species, while the other partner with an acidic C–H bond is responsible for generating the nucleophile. In this respect, the coupling of unactivated C_sp^3^_–H bonds may provide a more universal procedure with a high potential in all branches of synthetic chemistry.

Benzene-based iridium pincer complexes have been shown to be effective catalysts for various dehydrogenation reactions, including those that involve non-activated C_sp^3^_–H bonds.^5^ Previously, we reported on the synthesis of the complex *trans*-(PCyP)IrHCl (PCyP = {*cis*-1,3-bis-[(di-*tert*-butylphosphino)-methyl]cyclohexyl}) (**1**) and the attempted synthesis of (POCyOP)IrHCl (POCyOP = {*cis*-1,3-bis-[(di-*tert*-butyl-phosphinoxy)]cyclohexyl}), which resulted in complete dehydrogenation and aromatization of the ligand and formation of the known benzene-based (POCOP)IrHCl complex.^6^ We have also explored the potential of these aliphatic pincer complexes as dehydrogenation catalysts.^7^

The introduction of a cyclohexane moiety instead of a benzene ring into the pincer complex affects its thermal stability. This thermal instability is most likely related to the behavior of the ligand, and given the aliphatic nature of all the ligand C–H bonds, we reasoned that coordinatively-unsaturated species could give rise to interesting reactivity. One way to probe this was to investigate the ligand transformations using the iridium hydrido-phenyl complex, which closely resembles the elusive hydrido-alkyl intermediates involved in dehydrogenation reactions. Here we report on C–H activation reactions with the aliphatic ligand involving both the ligand backbone and the *tert*-butyl groups on the phosphine. This two-site activation is shown to lead to the first example of an intramolecular ring-closing reaction where two carbon atoms with non-activated, alkane-like C_sp^3^_–H bonds are joined to form a carbon–carbon double bond under extrusion of dihydrogen. The reaction is mediated by a pincer carbene complex and thus proceeds *via* quadruple C–H activations at a single metal center.

## Results and discussion

### Dynamic oxidative addition of benzene in a phenyl-hydride complex

**Scheme 1:**
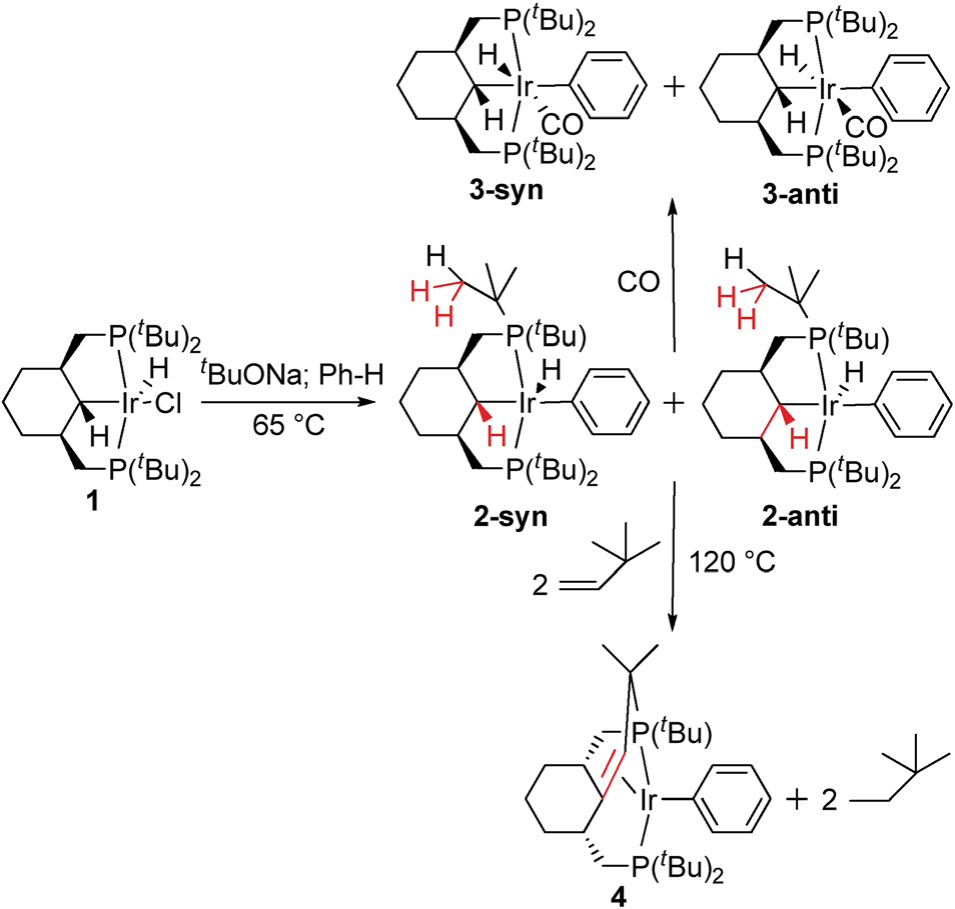
Reactions of complex **1** with benzene.

In the presence of ^*t*^BuONa, (PCyP)IrHCl (**1**) reacts with benzene, leading to the formation of extremely labile phenyl-hydride **2** (Scheme 1). At room temperature, complex **2** demonstrates dynamic behavior and its NMR signals are broad. Thus, a solution of **2** in methylcyclohexane-*d*_14_ reveals a broadened singlet at 63.1 ppm in the ^31^P{^1^H} NMR spectrum and a very broad signal at –47.8 ppm in the ^1^H NMR spectrum. At temperatures below *ca.* –10 °C, a P–H coupling with the high-field hydride resonance appears in the ^31^P{^1^H} NMR spectrum, while in the ^1^H NMR spectrum the hydride is observed as a triplet at –48.22 ppm (–40 °C, ^2^ *J*_PH_ = 12.3 Hz), shifted somewhat upfield at lower temperature. The slightly distorted shapes of the hydride and phosphorus resonances indicate the presence of a small amount of another compound with very close chemical shift values, most likely an isomer of **2** (in mesitylene-*d*_12_, the separation between the signals is better, and therefore two distinct hydride resonances are observed at –40 °C, and the amount of the isomer is bigger). It should be noted that the hydride signal remains significantly broadened (Δ*ν*_1/2_ = 16 Hz in the ^1^H{^31^P} spectrum at –40 °C) down to –90 °C, which may indicate the existence of an additional dynamic process (likely a rotation of the ^*t*^Bu groups). Also, two doublets and three triplets each integrating as 1H are observed in the aromatic region at low temperatures, consistent with *η* ^1^-coordination of the phenyl group. Complex **2** readily reacts with nitrogen (even with traces in low-quality argon) to give (PCyP)IrN_2_,^6^ and is sensitive to moisture. Hence, it can be considered as a source of a 14e (PCyP)Ir species. In this regard, **2** resembles the benzene-based complex [2,6-(^*t*^Bu_2_PCH_2_)_2_C_6_H_3_]Ir(H)Ph, which was found to undergo fast dissociative arene exchange at room temperature, with the rate being independent of the concentration of free benzene.^8^ Treating the coalescence of the two branches of the hydride-coupled doublet in the ^31^P{^1^H} NMR spectrum as a result of a simple exchange between two states of equal population, the barrier for the reductive elimination of benzene from **2** can be estimated as Δ*G* ^‡^ = 14.0 kcal mol^–1^ at –3 °C, which is very close to the value for the complex [2,6-(^*t*^Bu_2_PCH_2_)_2_C_6_H_3_]Ir(H)Ph (13.9 kcal mol^–1^ at –4 °C).^8^ The activation energy barrier for the reductive elimination of benzene from **2** was calculated by DFT, confirming the experimental data (Δ*G* ^‡^ = 16.0 kcal mol^–1^ at –3 °C).

The existence of two hydride signals for **2** at low temperatures can be explained by the lack of a horizontal plane of symmetry and the presence of two isomeric compounds – *syn* and *anti* with respect to the mutual orientation of C(α)–H and Ir–H. These isomers can be trapped by the addition of CO, giving 18e adducts **3-*syn*** and **3-*anti***, characterized by signals at 45.1 and 49.1 in the ^31^P{^1^H} NMR spectra as well as signals at –9.27 (td, ^3^ *J*_PH_ = 17.0, ^3^ *J*_HH_ = 1.6 Hz) and –8.76 (td, ^3^ *J*_PH_ = 19.0 Hz, ^3^ *J*_HH_ = 2.3 Hz) ppm in the ^1^H NMR spectra, correspondingly. At room temperature, three broadened signals from the phenyl group are observed for **3-*syn***; these do not demonstrate exchange with deuterobenzene up to 80 °C and therefore the broadening seems to be a result of phenyl group rotation. Interestingly, while for the major isomer (*syn*–*anti* ratio = 10 : 1) the *ortho* protons appear as a very broad signal almost at the coalescence point (Δ*G* ^‡^ = 14.0 kcal mol^–1^ at 25 °C), for the minor isomer two doublets are observed, as expected for the slow limit of rotation of the phenyl group. As far as the rate of rotation reflects the degree of crowding around the metal, it seems reasonable that the preference for the *syn* isomer is a result of mainly steric factors. In the IR spectrum, two intense bands are observed at 1951 cm^–1^ (**3-*syn***) and 1966 cm^–1^ (**3-*anti***), corresponding to C–O stretching vibrations, as well as two more broad, low-intensity bands at 2124 cm^–1^ (**3-*syn***) and 2199 cm^–1^ (**3-*anti***), from Ir–H. The latter bands moved to lower frequencies (but could not be unambiguously assigned) when deuterated benzene was used to prepare complex **3**, while the *ν*_CO_ bands moved to 1970 and 1983 cm^–1^, correspondingly.

**Fig. 1:**
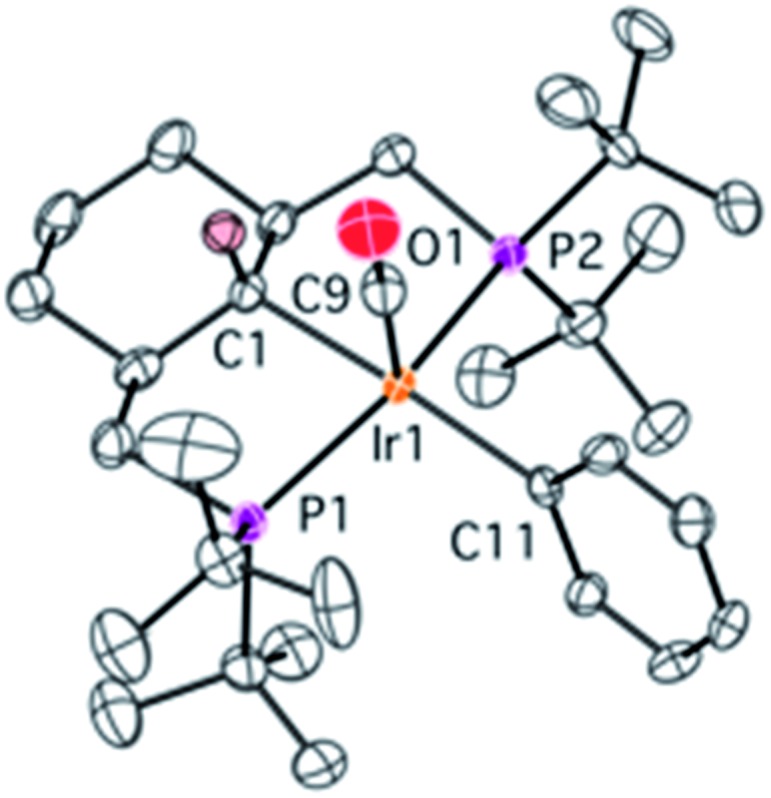
Molecular structure of complex **3-*anti***, with thermal ellipsoids at the 30% probability level. Hydrogen atoms, except for the one at C1, have been omitted for clarity. Selected bonds (Å) and angles (°): Ir1–C1 2.193(7), Ir1–C11 2.155(8), Ir1–C9 1.904(7), Ir1–P1 2.365(2), Ir1–P2 2.358(2), C9–O1 1.145(9), C1–Ir1–C11 176.5(3), P1–Ir–P2 158.06(7).

To confirm the arrangement of ligands around Ir, a ^13^CO-labelled sample of **3** was prepared. In the ^1^H NMR spectrum, the hydride resonances appear as doublets of triplets of doublets; the same multiplicity is observed for the CO signals in the non-decoupled ^13^C NMR spectrum. Large two-bond ^1^H–^13^C couplings of 42.9 Hz and 42.5 Hz for **3-*syn*** and **3-*anti***, correspondingly, indicate a mutual *trans* arrangement of the hydride and CO ligands.^9^ Therefore, the phenyl group occupies the position opposite to the pincer ligand. A three-bond ^1^H–^13^C coupling of 3.3 Hz is consistent with an *anti* mutual orientation of C(α)–H and Ir–CO in **3-*syn***, and the smaller coupling of 1.1 Hz implies a *syn* arrangement for **3-*anti***, according to the well-known dependence of couplings on the dihedral angle. The same trend is observed for ^3^ *J*_HH_, which is 1.6 Hz for **3-*syn*** (*syn* arrangement of C(α)–H and Ir–H) and 2.3 Hz for **3-*anti*** (*anti* arrangement of C(α)–H and Ir–H). Finally, despite a lower content in the mixture, X-ray quality crystals of **3-*anti*** were obtained, and the structure determination verified the conclusions based on spectroscopic data (Fig. 1).

### C–C coupling reaction in **2**

**Fig. 2:**
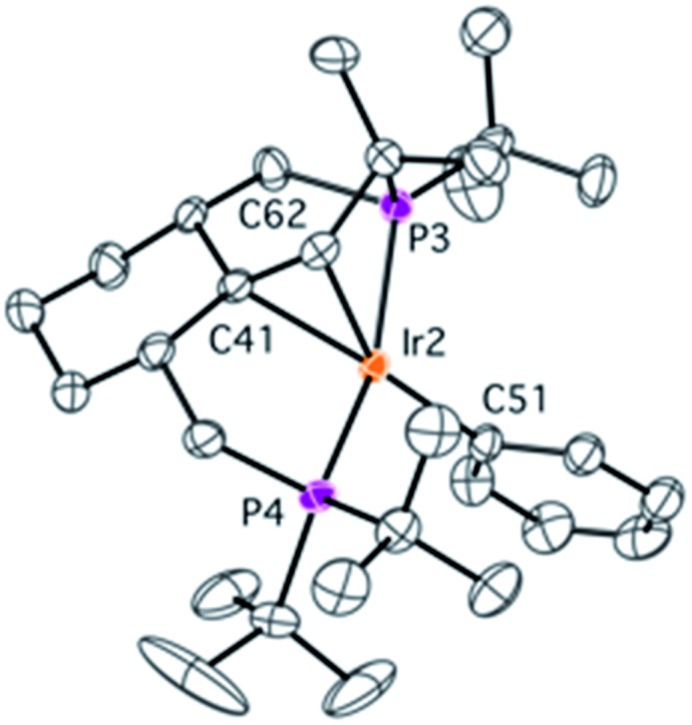
Molecular structure of complex **4** (one of two molecules from the asymmetric unit is shown) with thermal ellipsoids at the 30% probability level. Hydrogen atoms have been omitted for clarity. Selected bonds (Å) and angles (°): Ir2–C41 2.200(4), Ir2–C62 2.156(4), Ir2–C51 2.092(5), Ir2–P3 2.291(1), Ir2–P4 2.290(1), C41–C62 1.425(7), C41–Ir1–C51 164.0(2), P1–Ir–P2 160.05(5).

Upon heating a benzene solution of **2** to 120 °C, the NMR signals of **2** decrease in intensity and a new AX system appears in the ^31^P{^1^H} NMR spectrum. Gratifyingly, upon addition of *tert*-butylethylene as a hydrogen acceptor, complex **4** is formed in an almost quantitative yield. The ^1^H and, to some extent, ^13^C{^1^H} NMR spectra of compound **4** are very complex due to a number of overlapping signals. Doublets at 1.90 (^3^ *J*_PH_ = 13.0 Hz) and 0.91 (d, ^3^ *J*_PH_ = 9.7 Hz) ppm from methyl groups point to the activation of one of the *tert*-butyl groups. The =CH– proton resonates at 1.95 ppm (d, ^3^ *J*_PH_ = 20.5 Hz). In the ^13^C{^1^H} NMR spectrum the olefinic signals are observed at 76.6 (^2^ *J*_P2C_ = 13.1 Hz, ^2^ *J*_P1C_ = 4.8 Hz) and 49.5 (^2^ *J*_P2C_ = 12.3 Hz, ^2^ *J*_P1C_ = 1.3 Hz) ppm as doublets of doublets. An estimation (hampered due to overlap) of ^1^ *J*_CH_, which is around 156 Hz, is consistent with the expected sp^2^ hybridization of the olefinic carbons. The structure of complex **4** was confirmed using X-ray crystallography (Fig. 2). This unambiguously shows that the α-carbon and one of the methyl groups of the *t*-butyl have been coupled to form a new olefin functionality that the iridium(i) coordinates. The iridium atom has a distorted square-planar arrangement; average (from two molecules in the asymmetric unit) Ir–C bonds lengths of 2.16 and 2.20 Å as well as a C=C bond length of 1.42 Å fall in the range observed for electron-rich Ir olefin complexes.^10^ During the formation of **4** from **1**, three C_sp^3^_–H bonds are activated intramolecularly in addition to one external C_sp^2^_–H bond. Cyclometallation of *tert*-butyls^11^ or other groups^12,13^ bound to phosphorus is not unprecedented in the chemistry of iridium pincer complexes, but in the case of **4** it results in the formation of a new C–C double bond. To the best of our knowledge, this is the first example where the main product is the result of the formation of a C–C double bond from non-activated C_sp^3^_–H bonds.^14^

A few examples of a somewhat related, stoichiometric, dehydrogenative cross-coupling of C–H bonds to give olefin moieties have been reported in the literature, but they are all based on significantly more reactive benzylic C–H bonds of substituted arylphosphines.^15^

### Hydrogenation of the olefin complex **4**: C–C bond cleavage

**Scheme 2:**
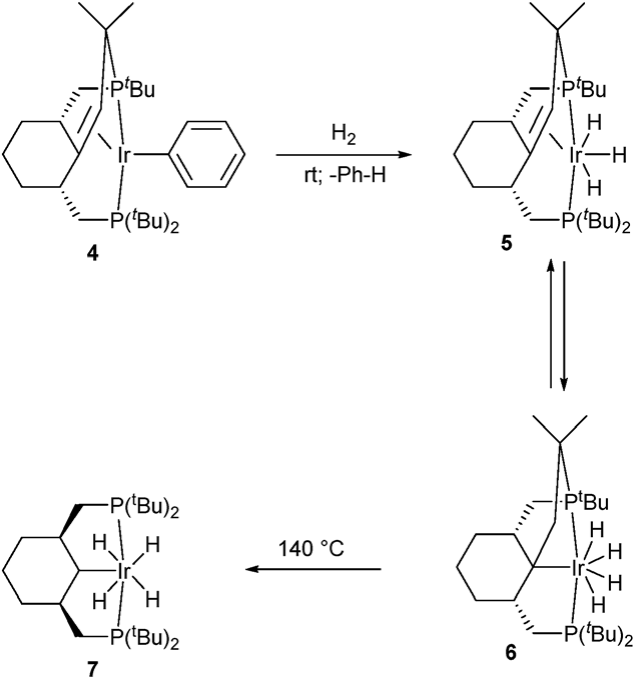
Reactions of complex **4** with hydrogen.

Interestingly, the process is reversible and the C=C bond in **4** can be cleaved under certain conditions. Thus, exposure of a solution of **4** in C_6_D_6_ at room temperature to 1 atm of hydrogen results in the simultaneous formation of a mixture of complexes **5** and **6** in a *ca.* 93 : 7 ratio (Scheme 2). This ratio is temperature and hydrogen pressure dependent, with the amount of **6** being raised under conditions favoring the solubility of H_2_ in benzene, and the equilibrium is established quite fast. For example, under 1.5 atm of H_2_, the proportion **5** : **6** is 74 : 26 at 4 °C, 86 : 14 at 25 °C and 98 : 2 at 80 °C. The ^31^P{^1^H} NMR spectrum of **5** consists of an AX system (75.7, 7.2 ppm, ^2^ *J*_P1P2_ = 344.0 Hz), while in the ^1^H NMR spectrum three separate hydride signals appear as rather complex multiplets, due to couplings with two non-equivalent phosphorus nuclei, two hydrides and the olefin moiety. From those, a ^2^ *J*_HH_ = 10.7 Hz should be mentioned as a rare example of a coupling between two mutually *trans* non-equivalent hydride ligands.^16^ The hydrides in complex **5** do not exchange positions up to 80 °C. In accordance with the increased oxidation state of the Ir atom, the NMR signals of the olefin moiety in **5** are shifted downfield compared to **4**; for example the –CH= proton resonates at 2.51 ppm (dd, ^3^ *J*_P2H_ = 21.7 Hz, ^3^ *J*_P1H_ = 3.3 Hz) and the quaternary carbon signal is observed at 81.3 ppm (m).

Complex **6** is characterized by an AB system (69.8, 63.3 ppm, ^2^ *J*_P1P2_ = 312.9 Hz) in the ^31^P{^1^H} NMR spectrum as well as a triplet resonance at –10.07 ppm (^2^ *J*_PH(avg)_ = 9.5 Hz) in the ^1^H NMR, integrating as 4H. 2D NMR spectra strongly argue that **6** is a product of insertion of the olefin moiety into one of the Ir–H bonds, which added one molecule of hydrogen. For instance, in contrast to **5**, no –CH– type carbons reveal a cross-peak with the hydride resonance in ^1^H–^13^C HMBC, while three correlations with –CH_2_– type carbons are observed, with two of them belonging to –CH_2_P– groups and one to the former –CH= group.

When a mixture of **5** and **6** is heated at 140 °C for 24 h under an atmosphere of hydrogen, a quantitative conversion to tetrahydride complex **7** is observed, where the initial structure of the pincer ligand is recovered. Hence, the observed formation of a C–C double bond *via* C_sp^3^_–H activation is fully reversible, and proceeds in the forward direction when a hydrogen acceptor like *tert*-butylethylene is used, and in the reverse direction when H_2_ pressure is applied. Remarkably, the transformation from **5** to **7** corresponds to a net rupture of a C–C double bond, while the transformation from **6** to **7** is a net cleavage of a nearly unstrained, unactivated C_sp^3^_–C_sp^3^_ bond, with few precedents previously reported.^17^ Together with the tandem catalytic systems for alkane metathesis,^18^ this process is a rare example of an organometallic compound capable of a net cleavage of unactivated C_sp^3^_–C_sp^3^_ bonds.

### Computational studies

**Fig. 3:**
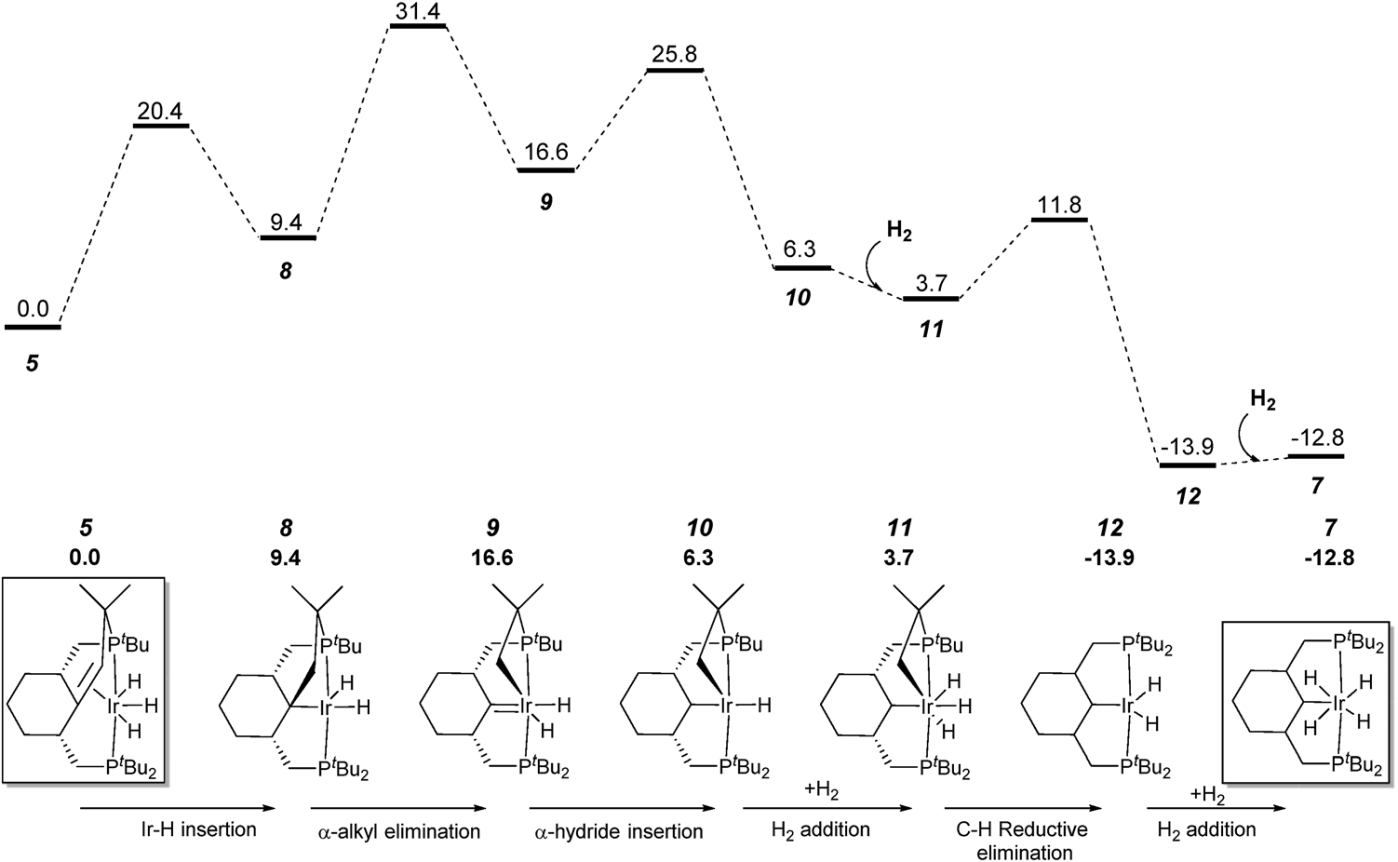
Profile of the calculated relative *G* (kcal mol^–1^) for the formation of complex **7** from complex **5** *via* carbene complex **9**.

To understand each step of the whole transformation we performed DFT calculations on the key reactions for forming and breaking C–C bonds, based on the experimentally observed species. Several mechanistic scenarios were considered for the formation of complex **7**; the one with the lowest barrier and that is fully consistent with the experimental conditions is depicted in Fig. 3. First, from complex **5**, a double bond insertion into the Ir–H bond leads to intermediate **8**, which can be transformed to the experimentally observed Ir(v) complex **6** *via* the oxidative addition of dihydrogen. The calculated barrier of *ca.* 20 kcal mol^–1^ is in qualitative agreement with the rate of establishment of the equilibrium (a few minutes) at ambient temperature. Intermediate **8** undergoes an α-alkyl elimination and carbene **9** is formed. The activation barrier is calculated at 31.4 kcal mol^–1^ *via* transition state **TS8–9**. Complex **9** proceeds through an α-hydride insertion to form intermediate **10**, which, under hydrogen atmosphere conditions, undergoes an H_2_ addition that finally forms the Ir(v) complex **11**. The transition state **TS9–10** is calculated at 25.8 kcal mol^–1^ on the free energy surface. This step is followed by C–H reductive elimination by Ir–C_sp^3^_ and Ir–H, which leads to formation of a new C_sp^3^_–H bond in the pincer ligand. The free energy barrier *via* transition state **TS11–12** is calculated at 11.8 kcal mol^–1^.

Ultimately, the process finishes with an oxidative addition of one dihydrogen molecule, leading to the Ir(v)H_4_ complex **7**. The overall activation free energy corresponds to the C–C bond cleavage step (31.4 kcal mol^–1^).

**Fig. 4:**
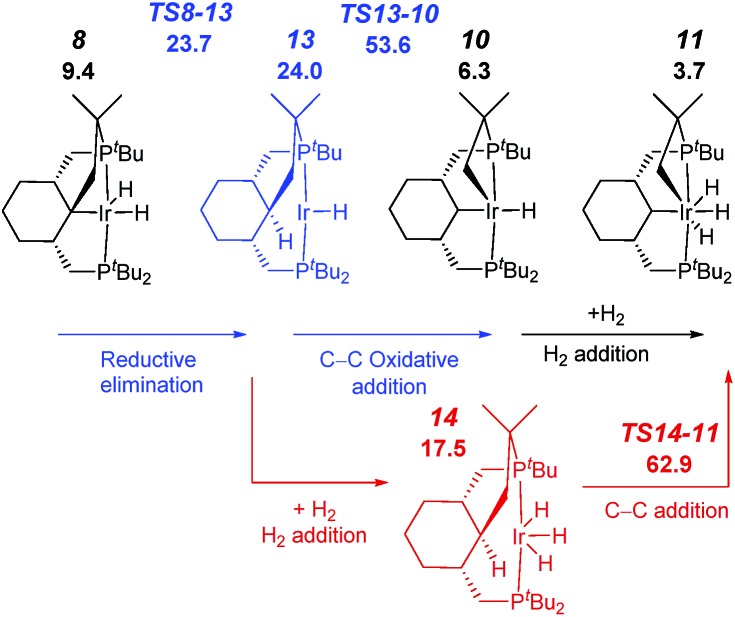
Profile of the calculated relative *G* (kcal mol^–1^) for the formation of complex **7** from complex **5** *via* reductive elimination from Ir(iii) (blue) or Ir(v) (red).

We also considered alternative mechanisms from **8**, involving *inter alia* C–H reductive elimination to give an alkane intermediate, which could be followed by C_sp^3^_–C_sp^3^_ oxidative addition (Fig. 4, red and blue pathways). However, the activation energies associated with this process were found to be prohibitively high. C–C bond oxidative addition/elimination at the metal have high barriers at least in part due to repulsion between the bulky alkyl ligands. The suggested mechanism is supported by the experimental observation of an α-hydrogen migration to Ir during the formation of a carbene complex (see below). Also, a number of R^1^ migrations from an R^1^M=CR^2^R^3^ fragment to M–CR^1^R^2^R^3^ are known,^19^ with a few precedents reported for vinylidene pincer complexes.^20^ It should be noted that for the somewhat-related coupling reaction involving benzylic C–H bonds, a similar reaction sequence was also proposed.^15b,c^ Interestingly, the previous examples where unactivated C_sp^3^_–C_sp^3^_ bonds were cleaved proceeded *via* another mechanism, namely β-carbon elimination.^17^ In addition, several examples of 1,2-shifts of methyl and benzyl groups to give arenium complexes (organometallic analogs of Wheland intermediates), which likely proceed through electrophilic attack of R^+^ on C_ipso_, are known; these may be followed by subsequent β-elimination.^21,22^

**Fig. 5:**
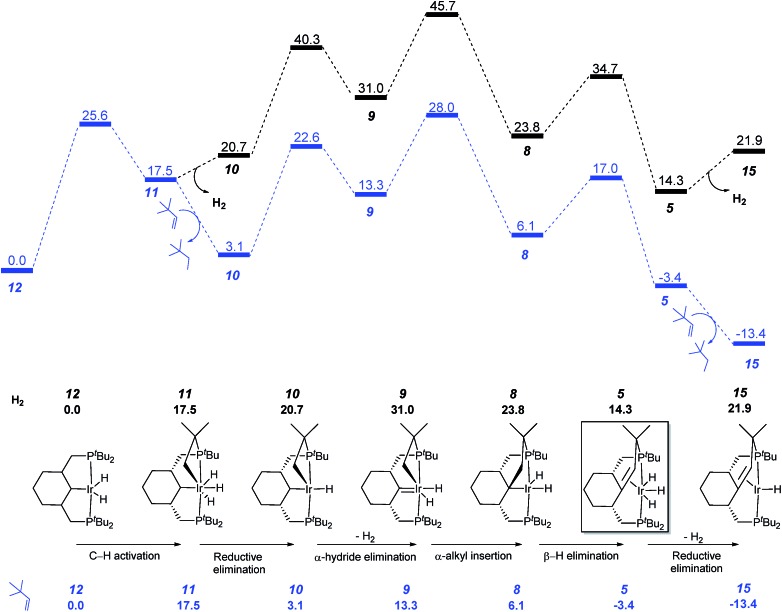
Profile of the calculated relative *G* (kcal mol^–1^) for the formation of complex **5** with and without hydrogen acceptor.

As mentioned before, the formation of the C–C double bond is reversible, proceeding in one direction or other depending on the reaction conditions. In agreement with the experiment, under hydrogen atmosphere, formation of complex **7** is favored, while in the presence of a hydrogen acceptor, such as *tert*-butylethylene, the reverse reaction occurs leading to the olefin complex **5** (Fig. 5). In the reaction with *tert*-butylethylene, the overall activation energy barrier corresponds to the C–C bond formation step (**TS9–8**) and is calculated at 28.0 kcal mol^–1^.

### Dehydrogenation of the cyclohexyl ring

**Scheme 3:**
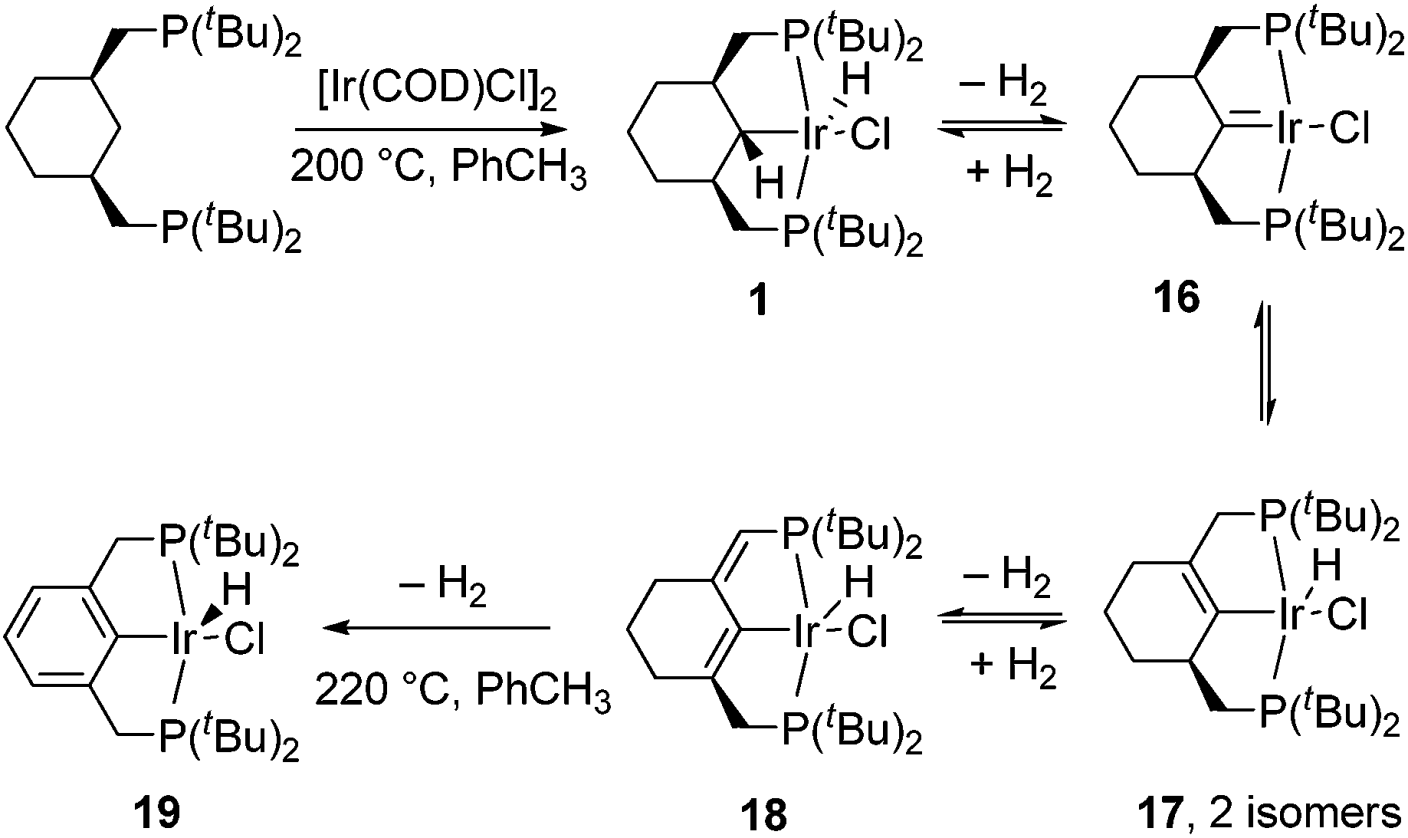
Aromatization of the cyclohexane-based pincer ligand.

For comparison and to gain more insight into the process, we attempted the thermolysis of the parent, less electron-rich hydrido-chloride complex **1**. Heating (PCyP)IrHCl (**1**) in toluene at higher temperatures reveals that it undergoes aromatization, and several intermediates were detected by NMR spectroscopy during this process (Scheme 3). All steps before aromatization are reversible, and carrying out the reaction in a sealed flask results in the simultaneous presence of compounds **1**, **16**, **17**, **18**, and **19** in the reaction mixture; to drive the process to completion it is necessary to purge the flask with Ar several times or to add a hydrogen acceptor like *tert*-butylethylene. In a control experiment, a mixture of **1**, **16**, **17**, and **18** was indeed converted to **1** under a H_2_ atmosphere at 155 °C. The ratio of compounds **1**, **16**, **17**, **18**, and **19** depends on the conditions, with the percentages of the more dehydrogenated products increasing with increasing temperature. Despite a number of attempts, including solution and solid-state thermolysis of **1** under various conditions, we were unable to obtain complex **16** in a high yield and isolate it from other products, and so far **16** and **17** have been characterized *in situ*.

**Scheme 4:**
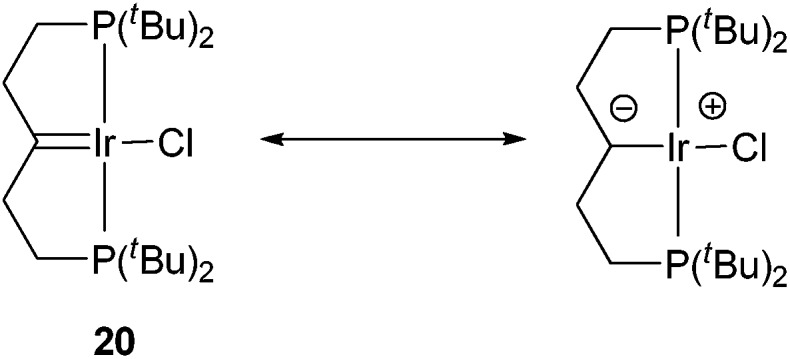
Shaw's carbene complex and the proposed contribution of the ylide structure.

The aromatization process begins with the formation of carbene complex **16**, which exhibits a singlet in the ^31^P{^1^H} NMR spectrum at 66.8 ppm and a remarkable high-field signal at –4.18 ppm in the ^1^H NMR spectrum from the β-C–H groups, probably due to the magnetic anisotropy effect of the C=Ir bond. The ^13^C{^1^H} NMR spectrum reveals a resonance at 246.9 ppm, corresponding to the α-carbon. This value dramatically differs from the 66.6 ppm reported by Shaw,^23^ who proposed the contribution of an ylide-type structure for the related complex **20** (Scheme 4) in order to explain the deviation of the observed chemical shift from that expected for carbene complexes at around 200 ppm. The ylide character of Shaw's complex has been mentioned in several papers and has been the subject of some discussion.^24^ Based on our data, we suggest that there is no reason to invoke an ylide structure for **16**, and probably not for **20** either. Complex **20** was characterized in a mixture where its concentration was fairly low, and the overall number of ^13^C signals observed by Shaw was three instead of the five expected from the symmetry of complex **20**. Therefore, given the similarity of complexes **20** and **16**, it could very well be that the signal at 66.6 ppm comes from the –CH_2_– groups adjacent to the carbene moiety since the analogous –CH–groups in complex **16** resonate at 75.0 ppm, while the true low-intensity, low-field signal of the carbene carbon atom of **20** was not observed. In fact, according to the calculations, the bonding pattern in complex **16** is close to those in Fisher-type carbenes and thus **16** is better described as an Ir(i) compound (see ESI†).

The formation of complex **16** requires high temperature and at these conditions, **16** readily turns into the isomeric compounds **17**, with the equilibrium shifted to the side of the latter complexes. The ^31^P{^1^H} NMR spectrum reveals two isomers of **17**, each with an ABX system (67.7, 64.9 ppm, ^2^ *J*_P1P2_ = 336.8 Hz and 66.6, 63.0 ppm, ^2^ *J*_P1P2_ = 335.0 Hz; high-field hydride signals are not decoupled), which indicates the presence of two non-equivalent phosphorus nuclei and a lack of the usual symmetry of the molecule. The corresponding hydride signals are observed at –41.25 (t, ^2^ *J*_P1H_ = ^2^ *J*_P2H_ = 13.2 Hz) and –44.63 (t, ^2^ *J*_P1H_ = ^2^ *J*_P2H_ = 12.3 Hz) ppm as apparent triplets, due to virtually equal coupling constants with both P atoms.^25^ The ^13^C{^1^H} NMR spectrum confirms the presence of one tetra-substituted double bond per molecule.

**Scheme 5:**
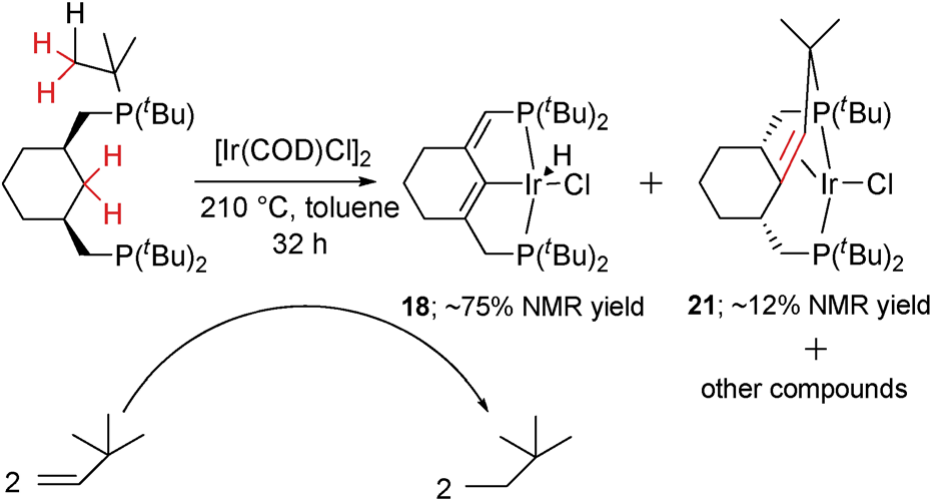
Quadruple C–H activation of the cyclohexane-based pincer ligand.

Further dehydrogenation results in the formation of diene complex **18**, which again is characterized by an ABX system in the ^31^P{^1^H} NMR spectrum (67.7, 60.3 ppm, dd, ^2^ *J*_P1P2_ = 334.3), as well as a doublet (^2^ *J*_PH_ = 8.0 Hz) at 5.61 ppm from the olefinic proton and an apparent triplet at –43.30 (^2^ *J*_P1H_ = ^2^ *J*_P2H_ = 12.7 Hz) ppm from the hydride. The =CH– group appears at 117.4 in the ^13^C{^1^H} spectrum and a large ^13^C–^31^P coupling of 44.4 Hz, together with 2D spectra, confirms the position of the double bond near the phosphorus atom. Under the conditions specified in Scheme 5, the relative kinetic stability of **18** allows an accumulation (up to 75%) in the reaction mixture, from which **18** can be isolated by chromatography.

### C–C coupling reaction in complex **1**

**Fig. 6:**
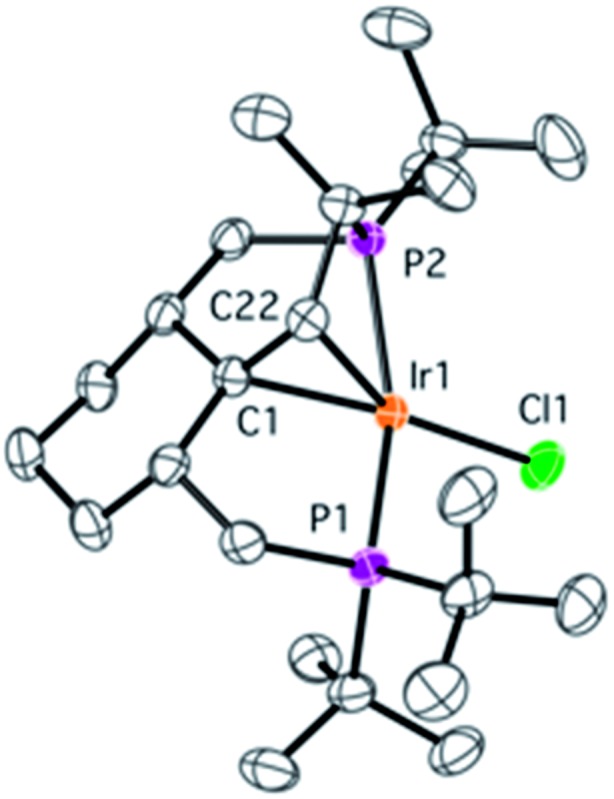
Molecular structure of complex **21** with thermal ellipsoids at 30% probability level. Hydrogen atoms have been omitted for clarity. Selected bonds (Å) and angles (°): Ir1–C1 2.142(4), Ir1–C22 2.112(4), Ir1–Cl1 2.380(1), Ir1–P1 2.2857(9), Ir1–P2 2.3153(9), C1–C22 1.441(5), Cl1–Ir1–C1 163.4(1), P1–Ir–P2 160.24(3).

During NMR spectroscopic monitoring of the formation of **18**, the appearance of a new AX system (66.3, 23.7 ppm, ^2^ *J*_P1P2_ = 370.0 Hz) was detected in the ^31^P{^1^H} spectrum. The yield of the new compound **21** was low, but we were able to isolate it by crystallization. The NMR features of **21** are similar to those of **4**, with signals for the olefinic proton and carbons being significantly high-field shifted, and the X-ray structure of **21** (Fig. 6) confirms that a similar olefin complex has formed. The C–C distance (1.441(5) Å) is comparable to that in **4** and the Ir–C bonds (2.142(4) and 2.112(4) Å) are somewhat shorter. As shown in Scheme 5, four non-activated C_sp^3^_–H bonds are cleaved during the synthesis of **21** (starting from the ligand precursor), making this process a rare example of a quadruple C–H activation at a single metal center. It also shows that the C–C coupling is a general reaction for this class of compounds.

While for complexes **4** and **21** high ligand binding energy did not allow the determination of a catalytic cycle, we believe that this reactivity pattern can form a basis for future catalytic cross-couplings of non-activated C_sp^3^_–H and possibly other C–H bonds under relatively mild conditions. In addition, whereas one of the reasons for the unreactive nature of C_sp^3^_–C_sp^3^_ bonds is their low exposure to metal atoms due to the screening by C–H or other bonds, we here show that at least in some cases such screening can be used in a beneficial way, during which simple metallation of one of the C–H bonds leads to a rearrangement where a purely aliphatic fragment is cut into two others.

## Conclusions

In conclusion, we have presented the C–H activation reactivity of cyclohexane-based iridium pincer complexes. The major difference between these systems and their arene-based counterparts is the non-innocent character of the pincer ligand, where both α- and β-hydrogens can be eliminated. This opened up an unprecedented reactivity, where non-activated C_sp^3^_–H bonds extrude two molecules of dihydrogen in the formation of a C–C double bond. This process is reversible and upon pressurizing with H_2_ the resulting C–C bond can be hydrogenated to recover the ligand structure; remarkably, this happens *via* a net C_sp^3^_–C_sp^3^_ bond cleavage. Mechanistic studies indicate that a key step to open up such reactivity is the formation of the carbene complex, which, *via* migratory insertion or deinsertion into an Ir–C bond, is responsible for C–C bond formation and cleavage. This knowledge will hopefully enable a catalytic variety of this transformation allowing the intermolecular formation of double bonds from the coupling of two non-activated aliphatic carbons.
