# Supplementary material for: Formation of a C–C double bond from two aliphatic carbons. Multiple C–H activations in an iridium pincer complex
Source: Chem Sci. 2015 Jan 26;6(3):2060–7. doi: 10.1039/c4sc03839h (PMC5496475; doi:10.1039/c4sc03839h)
Supplement: Supplementary file 1 [file SC-006-C4SC03839H-s001.pdf]

Electronic Supplementary Material (ESI) for Chemical Science.
This journal is © The Royal Society of Chemistry 2015

Electronic Supporting Information for

Formation of a C–C double bond from two aliphatic carbons. Multiple C–H activations in an iridium pincer complex

Alexey V. Polukeev, Rocío Marcos, Mårten S. G. Ahlquist and Ola F. Wendt*

Centre for Analysis and Synthesis, Department of Chemistry, Lund University, P.O. Box 124, 221 00 Lund, Sweden, and Division of Theoretical Chemistry & Biology, School of Biotechnology, KTH Royal Institute of Technology, Stockholm 106 91, Sweden.

*correspondence to: ola.wendt@chem.lu.se

Table of contents

1. Experimental part S2
2. Computational part S12
Summary of additional computational results S12
Calculated energies for all the species S29
Cartesian coordinates for all the species calculated S32
3. NMR Spectra of 3, 5, 6, 7, and 21 S66
4. References S72

1. Experimental part

General considerations. All manipulations were conducted under an inert gas atmosphere using standard Schlenk, high vacuum line and glovebox techniques unless otherwise stated. All solvents were distilled under vacuum from Na/benzophenone. Hydrocarbon deuterated solvents were distilled under vacuum from Na/benzophenone, CD_2Cl_2 was distilled under vacuum from calcium hydride. NMR spectra were recorded on a Varian Unity INOVA 500 MHz instrument. ^{1}H and ^{13}C NMR chemical shifts are reported in parts per million and referenced to the signals of deuterated solvents. $^{31}P\{^{1}H\}$ NMR chemical shifts are reported relative to external 85% solution of phosphoric acid. Systems of two coupled nuclei are treated as AB for $\Delta\nu/J < 4$ and as AX for $\Delta\nu/J \geq 4$. In some $^{31}P\{^{1}H\}$ and $^{13}C\{^{1}H\}$ NMR spectra decoupling of high-field hydride ligands was incomplete or totally absent; in the latter case coupling constants are indicated in the text. Assignments of signals were confirmed using COSY, TOCSY, NOESY, ^{1}H-^{13}C HSQC, ^{1}H-^{13}C and ^{1}H-^{31}P HMBC spectra. When multiplets cannot be resolved in complex ^{1}H spectra due to overlap with other resonances, the center of the signal according to the 2D spectrum is reported. IR spectra were recorded on a Bruker Alpha spectrometer. Elemental analyses were performed by H. Kolbe Microanalytisches Laboratorium, Mülheim an der Ruhr, Germany. Despite several attempts, satisfactory elemental analysis for **3** was not obtained, presumably due to decomposition; nevertheless, the compound is pure as indicated by NMR spectra.

Synthesis of hydrido-phenyl complex 2. A Straus flask was charged with (PCyP)IrHCl (**1**) (0.025 g, 0.040 mmol) and ^{t}BuONa (0.0057 g, 0.060 mmol) inside a nitrogen atmosphere glovebox, and 5 ml of C_6H_6 were vacuum-transferred to this mixture followed by refilling with Ar. After heating the flask on an oil bath for 15 h at 65 ºC, complex **2** is formed almost quantitatively. This compound is extremely unstable and readily reacts with traces of oxygen, nitrogen and moisture, as well as traces of impurities in commercial solvents, in particular with those in mesitylene-d_{12}.

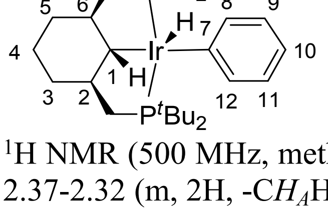

^{1}H NMR (500 MHz, methylcyclohexane-d_{14}): δ 7.2 and 6.9 (very br, overlapping, Ar-H), 2.37-2.32 (m, 2H, -CH_AH$_B$-P), 2.16-2.12 (m, 2H, 3-H$_A$ + 5-H$_A$), 2.92-1.87 (m, 1H, 4-H$_A$),

1.68 (t, $^3J_{HH}$ = 10.3 Hz, 1-H), 1.63-1.56 (overlapping m, 2-H + 6-H and -CH$_A$$H_B$-P), 1.28 (overlapping, 4-H$_B$), 1.17 (vt, $|^3J_{PH} + ^5J_{PH}|$ = 12.4 Hz, 18H, 2 ^{t}Bu), 1.05 (vt, $|^3J_{PH} + ^5J_{PH}|$ = 12.3 Hz, 18H, 2 ^{t}Bu), 0.99-0.91 (m, 2H, 3-H$_B$ + 5-H$_B$), -47.8 (very br, Ir-H).
^{1}H NMR (500 MHz, methylcyclohexane-d_{14}, -40 ºC, selected signals): δ 7.46 (d, $^3J_{HH}$ = 7.3 Hz, 1H, 12-H), 7.43 (d, $^3J_{HH}$ = 7.3 Hz, 1H, 8-H), 6.89 (apparent dt, $^3J_{HH}$ = 7.3 Hz, $^4J_{HH}$ = 1.6 Hz, 1H, 11-H), 6.67 (apparent dt, $^3J_{HH}$ = 7.3 Hz, $^4J_{HH}$ = 1.6 Hz, 1H, 9-H), 6.52 (apparent t, $^3J_{HH}$ = 7.3 Hz, 1H, 10-H), -48.22 (br t, $^2J_{PH}$ = 12.3 Hz, 1H, Ir-H). ^{31}P{^{1}H} NMR (202 MHz, methylcyclohexane-d_{14}, -40 ºC): δ 63.1 (s).

Synthesis of isomeric hydrido-phenyl carbonyl complexes 3. To a degassed solution of complex **2** in C_6H_6, prepared as described previously from complex **1** (0.025 g, 0.040 mmol) and ^{t}BuONa (0.0057 g, 0.060 mmol), 1 atm of CO was added, and the solution was stirred for 30 min, accompanied with a color change from red to pale yellow. After this, 5 ml of degassed water was added, the mixture was stirred for 10 min, the organic phase was decanted and passed through a thin layer of Celite; the Celite was washed with small portion of benzene. Evaporation of the volatiles gave **3** in ca. 95% purity according to NMR, further purification could be achieved by flash chromatography on basic alumina using C_6H_6 as eluent. **3** is obtained as white powder; yield 0.024 g, 86%.

Characterization of complex **3-*syn***:

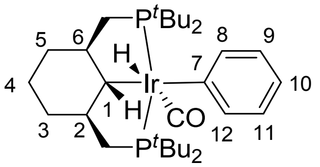

^{1}H NMR (500 MHz, C_6D_6): δ 8.30 (br, 2H, 8-H and 12-H), 7.10 (br m, 2H, 9-H + 11-H), 7.03 (br t, $^3J_{HH}$ = 7.1 Hz, 1H, 10-H), 2.20 (dd(vt), $^2J_{HH}$ = 13.6 Hz, $^3J_{HH}$ = 4.1 Hz, $|^2J_{PH} + ^4J_{PH}|$ = 7.0 Hz, 2H, CH_AH$_B$-P), 2.08-1.99 (m, 3H, 3-H$_A$ + 5-H$_A$ and 4-H$_A$), 1.70 (td, 1H, $^3J_{HH}$ = 10.2 Hz, $^3J_{H(IrH)}$ = 1.6 Hz, 1-H), 1.66-1.58 (m, 2H, 2-H + 6-H), 1.50 (overlapping, apparent qt, J_1 = 13.3 Hz, J_2 = 4.1 Hz, 1H, 4-H$_B$), 1.44 (overlapping, apparent t(vt), $^2J_{HH}$ = $^3J_{HH}$ = 13.6 Hz, $|^2J_{PH} + ^4J_{PH}|$ = 7.0 Hz, 2H, CH$_A$$H_B$-P), 1.25 (vt, $|^3J_{PH} + ^5J_{PH}|$ = 12.8 Hz, 18H, 2 ^{t}Bu), 1.12 (vt, $|^3J_{PH} + ^5J_{PH}|$ = 13.0 Hz, 18H, 2 ^{t}Bu), 1.03-0.95 (m, 2H, 3-H$_B$ + 5-H$_B$), -9.27 (td, $^2J_{PH}$ = 17.0 Hz, $^3J_{HH}$ = 1.6 Hz, 1H, Ir-H). ^{13}C{^{1}H} NMR (126 MHz, C_6D_6): δ 187.88 (m, CO), 149.1 (very br, 8-C and 12-C), 135.43 (t, $^2J_{PC}$ = 7.9 Hz, 7-C), 127.3 (very br, 9-C and 11-C), 121.01 (t, $^5J_{PC}$ = 0.9 Hz, 10-C), 51.19 (vt, $|^2J_{PC} + ^3J_{PC}|$ = 11.0 Hz, 2-C and 6-C), 39.96 (vt, $|^1J_{PC} + ^3J_{PC}|$ = 26.0 Hz, CH_2-P), 37.24 (vt, $|^1J_{PC} + ^3J_{PC}|$ = 20.0 Hz, $C(CH_3)_3$), 36.45 (vt, $|^1J_{PC} + ^3J_{PC}|$ = 25.4 Hz, $C(CH_3)_3$), 35.16 (s, 1-C), 34.42 (vt, $|^3J_{PC} + ^4J_{PC}|$ = 17.8 Hz, 3-C and 5-C), 31.38 (vt, $|^2J_{PC} + ^4J_{PC}|$ = 3.6 Hz, $C(CH_3)_3$), 30.79 (vt, $|^2J_{PC} + ^4J_{PC}|$ = 4.4 Hz, $C(CH_3)_3$), 28.08 (t, $^4J_{PC}$ = 1.6 Hz, 4-C). ^{31}P NMR (202 MHz, C_6D_6): δ 48.05 (br d). IR (hexane), cm^{-1}: 2124 (w, Ir-H), 1951 cm^{-1} (s, ν_{CO}).

Characterization of complex **3-*anti***:

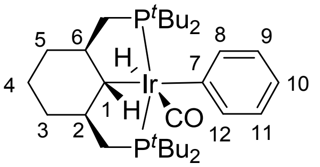

^{1}H NMR (500 MHz, C_6D_6): δ 8.39 (d, $^3J_{HH}$ = 7.1 Hz, 1H, 12-H), 8.21 (d, $^3J_{HH}$ = 6.6 Hz, 1H, 8-H), 2.03 (overlapping, CH_2-P), 1.74 (overlapping, 2-H + 6-H), 1.28 (overlapping,

1-H), 1.20 (vt, $|^3J_{PH} + ^5J_{PH}|$ = 13.0 Hz, 18H, 2 ^{t}Bu), 1.10 (overlapping, vt, $|^3J_{PH} + ^5J_{PH}|$ = 12.6 Hz, 2 ^{t}Bu), 0.89-0.81 (m, 2H, 3-H_B + 5-H_B), -8.76 (td, $^2J_{PH}$ = 19.0 Hz, $^3J_{HH}$ = 2.3 Hz, 1H, Ir-H). $^{13}C\{^1H\}$ NMR (126 MHz, C_6D_6): δ 188.6 (m, CO), 150.12 (s, 12-C), 147.90 (s, 8-C), 136.34 (t, $^2J_{PC}$ = 7.4 Hz, 7-C), 126.29 (9-C or 11-C), 120.88 (t, $^5J_{PC}$ = 0.9 Hz, 10-C), 53.02 (vt, $|^2J_{PC} + ^3J_{PC}|$ = 13.0 Hz, 2-C and 6-C), 37.88 (vt, $|^1J_{PC} + ^3J_{PC}|$ = 19.2 Hz, $C(CH_3)_3$), 37.57 (vt, $|^1J_{PC} + ^3J_{PC}|$ = 25.6 Hz, CH_2-P), 36.33 (overlapping, vt, $|^1J_{PC} + ^3J_{PC}|$ = 26.6 Hz, $C(CH_3)_3$), ca. 34.49 (overlapping, 3-C and 5-C), 30.26 (vt, $|^2J_{PC} + ^4J_{PC}|$ = 4.8 Hz, $C(CH_3)_3$), 30.79 (br, $C(CH_3)_3$), 27.49 (t, $^4J_{PC}$ = 1.8 Hz, 4-C).$^{31}P\{^1H\}$ NMR (202 MHz, C_6D_6): δ 49.11 (br d). IR (hexane), cm^{-1}: 2199 (w, Ir-H), 1966 cm^{-1} (s, ν_{CO}).

Synthesis of isomeric hydrido-phenyl carbonyl complexes 3 (*syn* and *anti*), labeled with ^{13}CO. Starting from complex **1** (0.015 g, 0.024 mmol), ^{t}BuONa (0.0034 g, 0.035 mmol) and ^{13}CO, complex **3-^{13}CO** (0.014 g, 84%) was prepared as described previously. **3-^{13}CO-*syn***, selected signals: ^{1}H NMR (500 MHz, C_6D_6): δ -9.26 (dtd, $^2J_{CH}$ = 42.9 Hz, $^2J_{PH}$ = 17.0 Hz, $^3J_{HH}$ = 1.6 Hz, 1H, Ir-H). $^{31}P\{^1H\}$ NMR (202 MHz, C_6D_6): δ 48.06 (m). ^{13}C NMR (126 MHz, C_6D_6) δ 187.89 (dtd, $^2J_{CH}$ = 42.9 Hz, $^2J_{PC}$ = 5.7 Hz, $^3J_{CH}$ = 3.3 Hz, CO). IR (hexane), cm^{-1}: 2117 (w, Ir-H), 1909 cm^{-1} (s, ν_{CO}). Calculated from ν_{12CO}: 1908 cm^{-1}. **3-^{13}CO-*anti***, selected signals: ^{1}H NMR (500 MHz, C_6D_6): δ -8.76 (dtd, $^2J_{CH}$ = 42.5 Hz, $^2J_{PH}$ = 19.0 Hz, $^3J_{HH}$ = 2.3 Hz, 1H, Ir-H). $^{31}P\{^1H\}$ NMR (202 MHz, C_6D_6): δ 49.11 (m). ^{13}C NMR (126 MHz, C_6D_6) δ 188.64 (dtd, $^2J_{CH}$ = 42.5 Hz, $^2J_{PC}$ = 6.2 Hz, $^3J_{CH}$ = 1.1 Hz, CO). IR (hexane), cm^{-1}: 2196 (w, Ir-H), 1925 cm^{-1} (s, ν_{CO}). Calculated from ν_{12CO}: 1922 cm^{-1}.

Synthesis of isomeric hydrido-phenyl carbonyl complexes 3-(D,C_6D_5) (*syn* and *anti*), labeled with deuterium. Starting from complex **1** (0.015 g, 0.024 mmol), ^{t}BuONa (0.0034 g, 0.035 mmol) and 1 ml of C_6D_6, complex **3-(D,C_6D_5)** (0.014 g, 83%) was prepared as described previously. **3-(D,C_6D_5)-syn**, selected signals: $^{31}P\{^1H\}$ NMR (162 MHz, C_6D_6): δ 48.0 (br). IR (hexane), cm^{-1}: 1970 cm^{-1} (s, ν_{CO}). **3-(D,C_6D_5)-anti**, selected signals: $^{31}P\{^1H\}$ NMR (162 MHz, C_6D_6): δ 49.0 (br). IR (hexane), cm^{-1}: 1983 cm^{-1} (s, ν_{CO}). An additional medium-intense band at 1991 cm^{-1} was also observed, presumably from decomposition product.

Synthesis of C-C coupling product 4. A Straus flask was charged with (PCyP)IrHCl (**1**) (0.050 g, 0.080 mmol) and ^{t}BuONa (0.012 g, 0.125 mmol) inside a nitrogen atmosphere glovebox, and 15 ml of C_6H_6 was vacuum-transferred to this mixture. The flask was refilled with Ar followed by addition of *tert*-butylethylene (0.05 ml, 0.388 mmol). Then the reaction mixture was heated on an oil bath for 18 h at 120 ºC, allowed to reach room temperature, and 10 ml of degassed water was added. The mixture was stirred for 10 min, the organic phase was decanted and passed through a thin layer of Celite; the Celite was washed with a small portion of benzene. The volatiles were evaporated and the residue was dried in vacuum to give **4** as red-orange powder in quantitative yield.

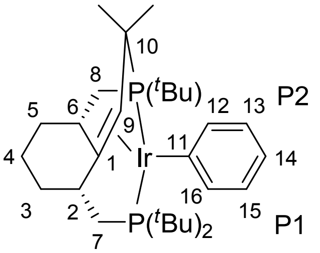

Anal. Calc. for $C_{30}H_{51}IrP_2$: C, 54.11; H, 7.72. Found C, 54.46, H, 7.66.
^{1}H NMR (500 MHz, C_6D_6): δ 7.78 (slightly br d, $^3J_{HH}$ = 7.5 Hz, 2H, 12-H + 16-H), 7.36 (apparent t, $^3J_{H(12+16)H(13+15)}$ = $^3J_{H(13+15)H14}$ = 7.5 Hz, 2H, 13-H +15-H), 6.90 (slightly br t, $^3J_{HH}$ = 7.5 Hz, 1H, 14-H) 2.00 (apparent tdd, $^2J_{HH}$ = $^2J_{P2H}$ = 12.7 Hz, $^3J_{HH}$ = 8.2 Hz, $^2J_{P1H}$ = 4.4 Hz, 1H, 8-H_A), 1.95 (d, $^2J_{P2H}$ = 20.5 Hz, 1H, 9-H), 1.93-1.84 (m, 3H, 3-H_A + 5H_A + 7H_A), 1.90 (d, $^3J_{PH}$ = 13.0 Hz, 3H, CH_3), 1.79 (apparent d of quintets, $^2J_{HH}$ = 13.1 Hz, $^3J_{HH}$ = 3.9 Hz, 1H, 4-H_A), 1.71-1.60 (m, 2H, 5-H_B + 7H_B), 1.54-1.48 (m, 1H, 3-H_B), 1.45-1.25 (overlapping m, 2-H + 4-H_B + 8-H_B + 6-H), 1.29 (d, $^3J_{P1H}$ = 11.9 Hz, 9H, ^{t}Bu at P1) overlapping with 1.27 (d, $^3J_{P1H}$ = 12.5 Hz, 9H, ^{t}Bu at P1), 0.91 (d, 3H, $^3J_{P2H}$ = 9.7 Hz, CH_3), 0.85 (d, 9H, $^3J_{P2H}$ = 13.5 Hz, ^{t}Bu at P2).
^{1}H NMR (500 MHz, toluene-d_8, -95 ºC, selected signals) 8.00 (slightly br d, $^3J_{HH}$ = 7.3 Hz, 1H, 12-H), 7.66 (slightly br d, $^3J_{HH}$ = 7.3 Hz, 1H, 16-H), 7.41 (two overlapping triplets, 2H, 15-H + 13-H,), 6.91 (overlapping, 1H, 14-H).
$^{13}C\{^1H\}$ NMR (126 MHz, C_6D_6): δ 173.69 (apparent t, $^2J_{P1C}$ = $^2J_{P2C}$ = 8.6 Hz, 11-C), 138.40 (apparent br d, $^3J_{PC}$ = 2.7 Hz, 12-C + 16-C), 126.49 (apparent t, $^4J_{PC}$ = 1.0 Hz, 13-C + 15-C), 120.30-120.27 (m, 14-C), 76.59 (dd, $^2J_{P2C}$ = 13.1 Hz, $^2J_{P1C}$ = 4.8 Hz, 1-C), 49.79 (dd, $^1J_{P2C}$ = 22.7 Hz, $^3J_{P1C}$ = 1.5 Hz, 10-C), 49.48 (dd, $^2J_{P2C}$ = 12.3 Hz, $^2J_{P1C}$ = 1.3 Hz, 9-C), 39.83 (dd, $^2J_{P1C}$ = 7.2 Hz, $^4J_{P2C}$ = 1.8 Hz, 2-C), 37.12 (dd, $^1J_{P1C}$ = 17.8 Hz, $^3J_{P2C}$ = 3.2 Hz, *C*(CH_3)$_3$ at P1), 35.76 (d, $^3J_{P2C}$ = 5.1 Hz, 5-C), 35.58 (dd, $^1J_{P1C}$ = 13.4 Hz, $^3J_{P2C}$ = 4.1 Hz, *C*(CH_3)$_3$ at P1), 34.95 (d, $^2J_{P2C}$ = 1.9 Hz, 6-C), 34.30 (d, $^1J_{P1C}$ = 19.7 Hz, 7-C), 32.44 (d, $^3J_{P1C}$ = 2.2 Hz, 3-C), 32.05 (dd, $^2J_{P1C}$ = 5.3 Hz, $^4J_{P2C}$ = 1.0 Hz, C(*C*H_3)$_3$ at P1), 31.74 (dd, $^2J_{P2C}$ = 9.4 Hz, $^4J_{P1C}$ = 3.7 Hz, *C*(CH_3)$_3$ at P2), 30.59 (dd, $^2J_{P1C}$ = 4.2 Hz, $^4J_{P2C}$ = 0.9 Hz, C(*C*H_3)$_3$ at P1), 29.46 (d, $^2J_{P2C}$ = 4.9 Hz, CH_3), 28.12 (dd, $^1J_{P2C}$ = 18.6 Hz, $^3J_{P1C}$ = 1.4 Hz, 8-C), 27.50 (dd, $^2J_{P2C}$ = 3.6 Hz, $^4J_{P1C}$ = 1.3 Hz, C(*C*H_3)$_3$ at P2), 25.30 (d, $^4J_{P2C}$ = 1.5 Hz, 4-C), 22.58 (dd, $^2J_{P2C}$ = 3.5 Hz, $^4J_{P1C}$ = 1.5 Hz, CH_3). $^{31}P\{^1H\}$ NMR (202 MHz, C_6D_6): δ 66.36 (d, $^2J_{P1P2}$ = 350.3 Hz, P1), 18.63 (d, $^2J_{P1P2}$ = 350.3 Hz, P2).

Hydrogenation of 4. Synthesis of complexes 5 and 6. A J. Young NMR tube was charged with complex **4** (0.023 g, 0.035 mmol) inside a nitrogen atmosphere glovebox, followed by vacuum-transfer of 0.7 ml of C_6D_6. The tube was refilled with 1 atm of H_2 and 93:7 mixture of compounds **5** and **6** was simultaneously formed according to NMR spectroscopy.
Characterization of complex **5**:

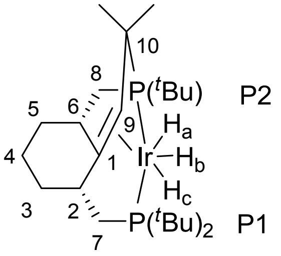

^{1}H NMR (500 MHz, C_6D_6): δ 2.51 (br dd, $^2J_{P2H}$ = 21.7 Hz, $^2J_{P1H}$ = 3.4 Hz, 1H, 9-H), 2.37-2.31 (overlapping, m, 1H, 6-H), 2.32-2.24 (overlapping, m, 1H, 8-H_A), 2.17-2.08 (m, 1H, 2-H), 2.05 (d, $^3J_{P2H}$ = 13.1 Hz, Me), 1.93 (apparent qd, J_1 = 12.0 Hz, J_2 = 3.8 Hz, 1H, 5-H_A), 1.87-1.73 (m, 3H, 5-H_B and 4-H_A and 3-H_A), 1.62-1.59 (m, 2H, 7-H_A + 7-H_B), 1.48-1.39 (m, 4-H_B), 1.36-1.31 (overlapping, m, 3-H_B), 1.33 (overlapping, d, $^2J_{P1H}$ = 12.0 Hz, ^{t}Bu at P1), 1.32 (overlapping, d, $^2J_{P1H}$ = 12.7 Hz, ^{t}Bu at P1), 0.96 (d, $^2J_{P2H}$ = 10.8 Hz, Me), 0.92 (d, $^2J_{P2H}$ = 14.1 Hz, ^{t}Bu at P2), -9.60 (dd of apparent quintets, $^2J_{P1H}$ = 13.8 Hz,

$^2J_{P2H}$ = 6.4 Hz, $^2J_{HaHb}$ ≈ $^2J_{HcHb}$ ≈ $^3J_{9\text{-}HHb}$ ≈ $^4J_{6\text{-}HHb}$ ≈ 1.4 Hz, 1H, H_b), -9.68 (m, $^2J_{PH}$ = 13.8 Hz, $^2J_{PH}$ = 13.0 Hz, $^2J_{HcHa}$ = 10.7 Hz, 1H, H_a), -12.24 (apparent br dt, $^2J_{PH}$ = 11.0 Hz, $^2J_{PH}$ = 10.7 Hz, $^2J_{HbHa}$ = 10.7 Hz, 1H, H_c). $^{13}C\{^1H\}$ NMR (126 MHz, C_6D_6): δ 81.26 (m, 1-C, $^2J_{P2C}$ = 14.6 Hz,1-C), 50.83 (d, $^1J_{P2C}$ = 27.3 Hz, 10-C), 46.11 (br d, $^2J_{P2C}$ = 14.6 Hz, 9-C), 42.03 (dd, $^2J_{P1C}$ = 6.3 Hz, $^3J_{P2C}$ = 3.0 Hz, 2-C), 38.32 (d, $^3J_{P2C}$ = 5.4 Hz, 5-C), 36.77 (m, $^2J_{P2C}$ = 0.7 Hz, 6-C), 35.83 (d, $^1J_{P2C}$ = 19.9 Hz, 8-C), 35.39 (dd, $^1J_{P1C}$ = 22.7 Hz, $^3J_{P2C}$ = 4.9 Hz, *C*(CH_3)$_3$ at P1), 35.21 (d, $^1J_{P1C}$ = 17.7 Hz, 7-C), 34.09 (d, $^3J_{P1C}$ = 1.6 Hz, 3-C), 32.08 (dd, $^1J_{P1C}$ = 5.2 Hz, $^3J_{P2C}$ = 1.1 Hz, C(*C*H_3)$_3$ at P1), 31.93 (d, $^1J_{P2C}$ = 2.0 Hz, CH_3), 31.82 (dd, $^1J_{P1C}$ = 15.6 Hz, $^3J_{P2C}$ = 4.5 Hz, *C*(CH_3)$_3$ at P1), 29.54 (br d, $^3J_{P1C}$ = 3.4 Hz, C(*C*H_3)$_3$ at P1), 26.92 (dd, $^1J_{P2C}$ = 9.1 Hz, $^3J_{P1C}$ = 6.6 Hz, *C*(CH_3)$_3$ at P1), 26.72 (dd, $^1J_{P2C}$ = 5.2 Hz, $^3J_{P1C}$ = 1.4 Hz, C(*C*H_3)$_3$ at P2), 25.85 (d, $^4J_{P2C}$ = 1.6 Hz, 4-C), 24.85 (dd, $^2J_{P2C}$ = 3.5 Hz, $^4J_{P1C}$ = 1.6 Hz, CH_3). $^{31}P\{^1H\}$ NMR (202 MHz, C_6D_6): δ 75.65 (m, $^2J_{P1P2}$ = 344.0 Hz, P1), 7.23 (m, $^2J_{P1P2}$ = 344.0 Hz, P2).

Characterization of complex **6**:

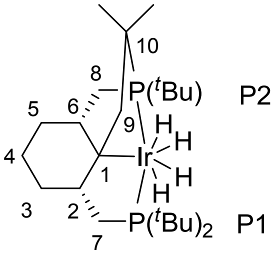

^1H NMR (500 MHz, C_6D_6): δ 2.57-2.50 (overlapping, m, 8-H_A), 2.33 (7-H_A), 2.22 (9-H_A), 2.14 (9-H_B), 2.01 (2-H), 1.98 (7-H_B), 1.79 (3-H_A), 1.77 (5-H_A), 1.53 ($^3J_{P2H}$ =14.1 Hz, CH_3), 1.49 (5-H_B), 1.44 (8-H_B), 1.42 (3-H_A), 1.28 (d, $^3J_{P1H}$ = 12.3 Hz, 9H, ^{t}Bu at P1), 1.28 (overlapping, CH_3)1.23 (d, $^3J_{P1H}$ = 12.2 Hz, ^{t}Bu at P1), 1.09 (d, $^3J_{P2H}$ = 13.1 Hz, 9H, ^{t}Bu at P2) 0.97 (6-H), -10.07 (apparent t, $^2J_{PH(avg)}$ = 9.5 Hz, 4H, Ir-H). $^{13}C\{^1H\}$ NMR (126 MHz, C_6D_6): δ 73.93 (d, $^2J_{P2C}$ = 7.9 Hz, 9-C), 54.05 (d, $^2J_{P2C}$ = 10.1 Hz, 1-C), 47.55 (dd, $^2J_{P1C}$ = 8.1 Hz, $^3J_{P2C}$ = 5.9 Hz, 2-C), 45.79 (d, $^2J_{P2C}$ = 3.2 Hz, 6-C), 43.56 (d, $^1J_{P2C}$ = 27.0 Hz, 10-C), 43.43 (d, $^1J_{P1C}$ = 24.2 Hz, 7-C), 39.08 (d, $^1J_{P2C}$ = 31.4 Hz, 8-C), 33.51 (dd, $^1J_{P1C}$ = 17.7 Hz, $^3J_{P2C}$ = 4.2 Hz, *C*(CH_3)$_3$ at P1), 31.77 (d, $^1J_{P1C}$ = 4.3 Hz, *C*(CH_3)$_3$ at P1), 30.83 (d, $^3J_{P1C}$ = 5.8 Hz, 3-C), 30.60 (d, $^2J_{P2C}$ = 6.5 Hz, CH_3), 30.39 (d, $^2J_{P1C}$ = 3.7 Hz, C(*C*H_3)$_3$ at P1), 29.92 (dd, $^2J_{P1C}$ = 4.7 Hz, $^4J_{P2C}$ = 1.3 Hz, C(*C*H_3)$_3$ at P1), 28.56 (dd, $^1J_{P2C}$ = 10.8 Hz, $^3J_{P1C}$ = 7.1 Hz, *C*(CH_3)$_3$ at P2), 28.28 (dd, $^2J_{P2C}$ = 5.1 Hz, $^4J_{P1C}$ = 1.7 Hz, C(*C*H_3)$_3$ at P2), 23.35 (dd, $^2J_{P2C}$ = 3.1 Hz, $^4J_{P1C}$ = 2.2 Hz, CH_3), 19.99 (d, $^3J_{P2C}$ = 15.8 Hz, 5-C), 19.08 (d, $^4J_{P2C}$ = 1.9 Hz, 4-C). $^{31}P\{^1H\}$ NMR (202 MHz, C_6D_6): δ 69.77 (m, $^2J_{P1P2}$ = 313.0 Hz, P1), 61.41 (m, $^2J_{P1P2}$ = 313.0 Hz, P2).

Hydrogenation of 4 with cleavage of the C–C bond. Synthesis of complex 7. A Straus flask was charged with complex **4** (0.015 g, 0.023 mmol) inside a nitrogen atmosphere glovebox, followed by vacuum-transfer of 5 ml of benzene. The flask was cooled to -196 ºC, refilled with 1 atm of H_2 and heated on an oil bath for 24 h at 140 ºC. Evaporation of the volatiles in vacuum afforded **7** as white powder in quantitative yield.

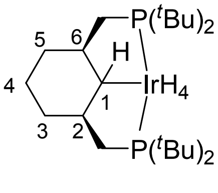

^{1}H NMR (500 MHz, C_6D_6) δ 2.42 (br t, $^3J_{HH}$ = 10.4 Hz, 1H, 1-H), 2.12-2.04 (m, 5H, CH_AH_B-P + 3-H_A and 5-H_A + 4-H_A), 1.86-1.78 (m, 2H, 2-H + 6-H), 1.62-1.53 (m, 1H, 4-H_B), 1.35 (apparent t(vt), $^2J_{HH}$ = $^3J_{HH}$ = 13.5 Hz, $|^2J_{PH} + ^4J_{PH}|$ = 7.8 Hz, 2H, CH_AH_B-P), 1.27 (vt, $|^3J_{PH} + ^5J_{PH}|$ = 12.6 Hz, 18H, 2 ^{t}Bu), 1.23 (vt, $|^3J_{PH} + ^5J_{PH}|$ = 12.6 Hz, 18H, 2 ^{t}Bu), 1.07-0.99 (m, 2H, 3-H_B and 5-H_B), -10.11 (td, $^2J_{PH}$ = 10.5 Hz, $^3J_{HH}$ = 2.2 Hz, 4H, Ir-H). ^{13}C{^{1}H} NMR (126 MHz, C_6D_6) δ 55.35 (vt, $|^2J_{PC} + ^3J_{PC}|$ = 14.9 Hz, 2-C and 6-C), 37.68 (vt, $|^1J_{PC} + ^3J_{PC}|$ = 25.3 Hz, CH_2-P), 34.73 (vt, $|^3J_{PC} + ^4J_{PC}|$ = 18.3 Hz, 3-C and 5-C) overlapping with 34.73 (s, 1-C), 32.48 (vt, $|^1J_{PC} + ^3J_{PC}|$ = 22.6 Hz, $C(CH_3)_3$), 32.02 (vt, $|^1J_{PC} + ^3J_{PC}|$ = 24.3 Hz, $C(CH_3)_3$), 30.06 (br s, $C(CH_3)_3$), 29.89 (vt, $|^2J_{PC} + ^4J_{PC}|$ = 5.7 Hz, $C(CH_3)_3$), 28.00 (t, $^4J_{PC}$ = 1.6 Hz, 4-C). ^{31}P{^{1}H} NMR (202 MHz, C_6D_6) δ 71.73 (s). IR (hexane, cm^{-1}): 2092 (m), 2060 (m), 1940 (vs).

Aromatization of the cyclohexane-based pincer pigand. A Straus flask was charged with PCyP ligand (0.085 g, 0.212 mmol), $[Ir(COD)Cl]_2$ (0.071 g, 0.106 mmol) and 4 ml of toluene as well as 0.1 ml of *tert*-butylethylene inside a nitrogen atmosphere glovebox. The flask was sealed, fully immersed into an oil bath and heated at 220 ºC for 48 h. The resulting solution was filtered through a thin layer of Celite and evaporated. The residue was dissolved in hexane, partially evaporated, and kept in the freezer (-20 ºC) overnight. The mother liquor was decanted and the residue was washed with a small amount of cold hexane to give $[2,6\text{-}(^tBu_2PCH_2)_2C_6H_3]IrHCl$ (**19**) as a red powder (0.105 g, 80%). NMR spectra are consistent with the literature data[1].

Comparison of the aromatization of the cyclohexane-based pincer ligand with and without hydrogen acceptor. Two Straus flasks were charged with (PCyP)IrHCl (**1**) (0.015 g, 0.024 mmol) and 5 ml of toluene inside a nitrogen atmosphere glovebox. To one of them, 0.1 ml of *tert*-butylethylene was added. The flasks were sealed, fully immersed into the same oil bath and heated at 220 ºC for specified times (note: the reaction is quite sensitive to the temperature gradients in the oil bath and to the heat flow, therefore the times may differ somewhat from the ones stated below). According to NMR monitoring, in the flask with *tert*-butylethylene, aromatization was complete within 48 h, while the flask without hydrogen acceptor contained ca. 55% **18**, 25% **1**, 10% of **19** and 10% of **21** (average from three experiments). Further heating, if the flask is not opened, does not result in any significant changes; if the flask is opened and purged with Ar several times the reaction slowly goes to completion. Therefore, for NMR monitoring of the acceptorless experiments, the flasks were not used further after being opened.

Synthesis of carbene complex 16 and mono-dehydrogenated isomeric complexes 17. Complex **1** (100 mg, 0.16 mmol) was placed into a Schlenk flask and heated in an oil bath for 9 h at 200 ºC while passing a slow stream of argon above the flask. Periodically, the flask was removed from the oil bath and the solid which sublimed above the oil level was returned to the bottom of the flask. While several solution and solid-state techniques for thermolysis of **1** were tried, this was found to produce the best yields of compounds **16** and **17**. A mixture containing 43% of starting material, 5% of **16**, 47% of **17** (28% of one isomer and 19% of another isomer), as well as 4% of **18** and 1% of **19** was formed, which was analyzed by NMR. Despite a number of tries, including solution and solid-state thermolysis of **1** under various conditions, we were unable to obtain complex **16** in a high yield and isolate it from the other products and so far **16** and

17 were characterized *in situ*. Attempts to isolate **16** and **17** by chromatography failed due to the instability of the compounds.

Characterization of complex **16**:

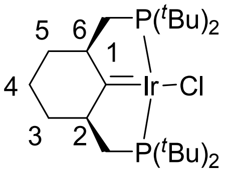

^{1}H NMR (500 MHz, CD_2Cl_2): selected signals: δ 2.29 (CH_2-P), 2.02 (3-H and 5-H), 1.57 (4-H_A), 1.47 (vt, $|^3J_{PH} + {}^5J_{PH}|$ = 13.0 Hz, 2 ^{t}Bu), 1.46 (4-H_B), -4.02 (m, 2H, 2-H + 6-H). ^{13}C{^{1}H} NMR (126 MHz, CD_2Cl_2): δ 246.93 (s, 1-C), 75.03 (vt, $|^2J_{PC} + {}^3J_{PC}|$ = 20.8 Hz, 2-C + 6-C), 30.63 (overlapping, CH_2-P) 30.52 (vt, $|^2J_{PC} + {}^4J_{PC}|$ = 5.8 Hz, C($C$$H_3$)$_3$), 29.93 (3-C +5-C), 26.63 (t, $^4J_{PC}$ = 1.9 Hz, 4-C). ^{31}P{^{1}H} NMR (202 MHz, CD_2Cl_2): δ 66.67 (s, 2P).

Characterization of isomeric compounds **17**:

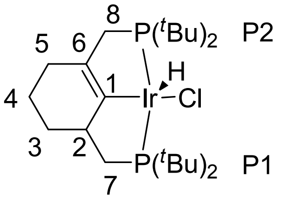

isomer 1

^{1}H NMR (500 MHz, C_6D_6): δ 2.52 (overlapping, 8-H_A), 2.51 (overlapping, 2-H), 2.25 (overlapping, 3-H_A + 3-H_B), 2.23 (overlapping, 8-H_B), 2.11 (overlapping, 5-H_A), 2.02 (overlapping, 7-H_A), 1.91 (m, 1H, overlapping 4-H and 4-H'), 1.65 (m, 1H, overlapping 4-H and 4-H'), 1.37-1.25 (overlapping, 4 ^{t}Bu), 1.24 (overlapping, 7-H_B), 1.13 (dt, $^2J_{HH}$ = 13.6 Hz, $^3J_{HH}$ = 6.9 Hz, 1H, 5-H_B), -41.25 (apparent t, $^2J_{P1H}$ = $^2J_{P2H}$ = 13.2 Hz, 1H, Ir-H). ^{13}C{^{1}H} NMR (126 MHz, C_6D_6): δ 138.01 (dd, $^2J_{P2C}$ = 10.7 Hz, $^3J_{P1C}$ = 5.1 Hz, 6-C), 137.52 (dd, $^2J_{CH}$ = 6.3 Hz, $^2J_{P2C}$ = 1.4 Hz, 1-C), 49.26 (dd, $^2J_{P1C}$ = 8.4 Hz, $^3J_{P2C}$ = 3.5 Hz, 2-C), 38.08 (d, $^1J_{P2C}$ =25.9 Hz, 8-C), 37.67-37.43 (overlapping, 3 C(CH_3)$_3$ and C(CH_3)$_3$ from isomer 2), 34.8 (overlapping, 5-C), 34.29 (overlapping, C(CH_3)$_3$), 32.42 (dd, $^1J_{P1C}$ = 16.3 Hz, $^3J_{P2C}$ = 0.8 Hz, 7-C), 30.87 (dd, $^2J_{PC}$ = 3.7 Hz, $^4J_{PC}$ = 1.3 Hz, C($C$$H_3$)$_3$), 30.47 (dd, $^2J_{PC}$ = 3.5 Hz, $^4J_{PC}$ = 1.3 Hz, C($C$$H_3$)$_3$), 30.39 (d, $^3J_{P1C}$ = 13.4 Hz, 3-C), 30.02 (dd, $^2J_{PC}$ = 3.5 Hz, $^4J_{PC}$ = 1.4 Hz, C($C$$H_3$)$_3$), 29.85 (dd, $^2J_{PC}$ = 3.3 Hz, $^4J_{PC}$ = 1.5 Hz, C($C$$H_3$)$_3$), 24.75-24.68 (m, overlapping 4-C and 4-C'). ^{31}P{^{1}H} NMR (202 MHz, C_6D_6): δ 67.65, 64.87 (AB part of ABX, $^2J_{P1P2}$ = 336.8 Hz, $^2J_{P1H}$ = $^2J_{P2H}$ = 13.2 Hz).

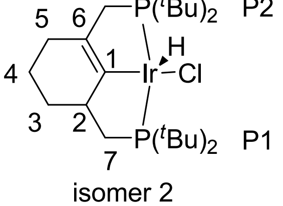

isomer 2

^{1}H NMR (500 MHz, C_6D_6): δ 2.67 (m, $^2J_{HH}$ = 17.2 Hz, 2H, 8-H_A), 2.54 (overlapping, 3-H_A), 2.35 (apparent ddt, $^2J_{HH}$ = 17.2 Hz, $^2J_{P2H}$ = 10.1 Hz, $^4J_{HH}$ = $^4J_{P1H}$ = 2.3 Hz, 8-H_B), 2.13 (overlapping, 3-H_B), 1.99 (overlapping, 7-H_A), 1.94 (overlapping, 5-H_A), 1.91

(overlapping, 4-H and 4-H'), 1.65 (m, 1H, overlapping 4-H and 4-H'), 1.41 (d, $^3J_{PH}$ = 12.4 Hz, ^{t}Bu), 1.38 (overlapping, 7-H_B), 1.31 (overlapping, 5-H_B), 1.30-1.23 (overlapping, 2 ^{t}Bu), 1.20 (d, $^3J_{PH}$ = 12.2 Hz, ^{t}Bu), -44.63 (apparent, $^2J_{P1H}$ = $^2J_{P2H}$ = 12.2 Hz, 1H, Ir-H). $^{13}C\{^1H\}$ NMR (126 MHz, C_6D_6): δ 143.35 (d, $^2J_{CH}$ = 3.6 Hz, 1-C), 139.43 (dd, $^2J_{P2C}$ = 12.0 Hz, $^3J_{P1C}$ = 5.3 Hz, 6-C), 52.44 (dd, $^2J_{P1C}$ = 9.1 Hz, $^3J_{P2C}$ = 3.9 Hz, 2-C), 37.99 (d, $^2J_{P2C}$ =25.8 Hz, 8-C), 37.67-37.43 (overlapping, $C(CH_3)_3$ and 3 $C(CH_3)_3$ from isomer 1), 36.31 (dd, $^1J_{PC}$ = 16.1 Hz, $^3J_{PC}$ = 4.7 Hz, $C(CH_3)_3$), 35.24 (dd, $^1J_{PC}$ = 16.6 Hz, $^3J_{PC}$ = 3.9 Hz, $C(CH_3)_3$), 34.5 (overlapping, 5-C), 32.65 (d, $^2J_{P1C}$ = 16.3 Hz, 7-C), 30.18 (d, $^3J_{P1C}$ = 14.2 Hz, 3-C), 29.48 (overlapping, dd, $^2J_{PC}$ = 3.6 Hz, $^4J_{PC}$ = 1.7 Hz, $C(CH_3)_3$), 29.11 (m, overlapping 2 $C(CH_3)_3$), 28.80 (dd, $^2J_{PC}$ = 3.7 Hz, $^4J_{PC}$ = 1.4 Hz, $C(CH_3)_3$), 24.75-24.68 (m, overlapping 4-C and 4-C'). $^{31}P\{^1H\}$ NMR (202 MHz, C_6D_6): δ 66.60, 62.96 (AB part of ABX, $^2J_{P1P2}$ = 335.0 Hz, J_{PH} not completely resolved due to broadening of signals).

Hydrogenation of 16, 17 and 18. A solution of complex **1** (0.015 g, 0.024 mmol) in 5 ml of toluene in a sealed Straus flask was heated for 2 h at 205 ºC, and a mixture of **1**, **16**, **17**, and **18** was formed according to NMR. The solution was degassed, cooled to -196 ºC and the flask was refilled with H_2. Upon heating the solution for 6 h at 155 ºC, a clean and quantitative conversion to **1** was observed.

Attempted hydrogenation of 19. A degassed solution of benzene-based complex **19** (0.015 g, 0.024 mmol) in 5 ml of toluene in a sealed Straus flask was cooled to -196 ºC and the flask was refilled with H_2. After this, the flask was fully immersed into an oil bath and heated; at temperatures up to 215 ºC, no formation of hydrogenated products was observed.

Synthesis of diene complex 18 and C-C coupling product 21. A Straus flask was charged with PCyP ligand (0.150 g, 0.374 mmol), $[Ir(COD)Cl]_2$ (0.126 g, 0.188 mmol) and 6 ml of toluene as well as 0.1 ml of *tert*-butylethylene inside a nitrogen atmosphere glovebox. The flask was sealed, fully immersed into an oil bath and heated at 210 ºC for 32 h. According to NMR, at that moment the reaction mixture contained 75% of **18** and 12% of **21**, and small amounts of **1** and **19** as well as traces of **16** and **17**. The volatiles were removed in vacuum and the residue was chromatographed under inert atmosphere on alumina, which was preliminary heated for 2 h at ca. 150 ºC under vacuum. Dry and degassed benzene-hexane 1:1 mixture was used as eluent. As far as color changes are difficult to distinguish and target compounds rapidly decompose on TLC plates, NMR monitoring was required. Compounds **21** and **1** were eluted first, together with some amount of **18**. These fractions were evaporated, the residue was dissolved in a minimum amount of hexane at 120 ºC into a Straus flask and the flask was allowed to reach ambient temperature. Relatively large, yellow crystals of **21** were formed which could be readily separated from the rest of solid material. Yield: 0.013 g, 6%. The middle fractions contained complex **18** in an up to 90% purity, while complex **16** eluted the last. Those middle fractions were evaporated and subjected to another chromatography on silica, which was preliminary heated for 2 h at ca. 150 ºC under vacuum. Dry and degassed benzene was used as eluent. Complex **19** was eluting first together with **18**, while the next colored fractions contained pure **18**. After evaporating the volatiles and drying in vacuum complex **18** was obtained as a red-orange powder. Yield: 0.036 g, 15%.

Characterization of complex **18**:

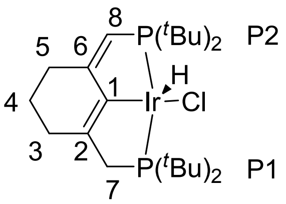

Anal. Calc. for $C_{24}H_{46}ClIrP_2$: C, 46.18; H, 7.43. Found C, 46.09, H, 7.47. ^{1}H NMR (500 MHz, C_6D_6): δ 5.61 (br d, $^2J_{P2H}$ = 8.0 Hz, 1H, 8-H), 2.66-2.61 (m, 1H, 5-H_A), 2.54-2.44 (m, 3H, 5-H_B and 7-H_A + 7-H_B), 2.28 (apparent dt, $^2J_{HH}$= 17.1 Hz, $^3J_{HH}$ = 5.4 Hz, 1H, 3-H_A), 2.15 (overlapping apparent dt, $^2J_{HH}$= 17.1 Hz, $^3J_{HH}$= 6.3 Hz, 1H, 3-H_B), 1.74-1.61 (m, 2H, 4-H_A + 4-H_B), 1.37-1.32 (m, 27H, 3 ^{t}Bu), 1.25 (d, $^2J_{PH}$ = 12.9 Hz, 9H, ^{t}Bu), -43.30 (apparent t, $^2J_{P1H}$ = $^2J_{P2H}$ = 12.7 Hz, 1H, Ir-H). ^{13}C{^{1}H} NMR (126 MHz, C_6D_6): δ 171.37 (dd, $^2J_{P2C}$ = 20.1 Hz, $^3J_{P1C}$ = 4.0 Hz, 6-C), 147.99 (dd, $^2J_{P1C}$ = 12.1 Hz, $^3J_{P2C}$ = 4.3 Hz, 2-C), 134.89 (ddd, $^2J_{CH}$ = 6.3 Hz, $^2J_{P2C}$ = 2.4 Hz, $^2J_{P1C}$ = 0.8 Hz, 1-C), 117.40 (d, $^1J_{P2C}$ = 44.4 Hz, 8-C), 38.63 (dd, $^1J_{PC}$ = 20.1 Hz, $^3J_{PC}$ = 3.3 Hz, *C*(CH_3)$_3$), 38.43 (d, $^1J_{P1C}$ = 26.7 Hz, 7-C), 37.10 (dd, $^1J_{PC}$ = 15.3 Hz, $^3J_{PC}$ = 3.9 Hz, *C*(CH_3)$_3$)), 34.62 (dd, $^1J_{P1C}$ = 17.0 Hz, $^3J_{P2C}$ = 3.5 Hz, *C*(CH_3)$_3$ at P1), 34.44 (dd, $^1J_{PC}$ = 21.1 Hz, $^3J_{PC}$ = 4.5 Hz, *C*(CH_3)$_3$), 30.37 (d, $^3J_{P2C}$ = 17.1 Hz, 5-C), 30.29 (d, $^3J_{P1C}$ = 14.2 Hz, 3-C), 29.95 (dd, $^2J_{PC}$ = 3.8 Hz, $^4J_{PC}$ = 1.4 Hz, C(*C*H_3)$_3$), 29.48 (m, 2 C(*C*H_3)$_3$ at P2), 29.36 (dd, $^2J_{P1C}$ = 3.8 Hz, $^4J_{P2C}$ = 1.4 Hz, C(*C*H_3)$_3$ at P1), 24.75 (d, $^4J_{P2C}$ = 1.0 Hz, 4-C). ^{31}P{^{1}H} NMR (202 MHz, C_6D_6): δ 67.73 (dd, $^2J_{P1P2}$ = 334.3, $^2J_{P1H}$ = 12.7 Hz, P1), 60.33 (dd, $^2J_{P21}$ = 334.3, $^2J_{P1H}$ = 12.7 Hz, P2).

Characterization of complex **21**:

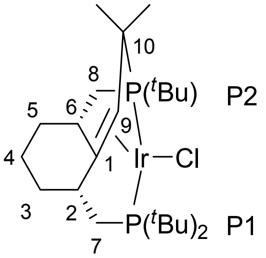

^{1}H NMR (500 MHz, C_6D_6): δ 2.00 (apparent tdd, $^2J_{HH}$ = $^2J_{P2H}$ = 13.4 Hz, $^3J_{HH}$ = 8.4 Hz, $^4J_{P1H}$ = 5.0 Hz, 1H, 8-H_A), 1.92-1.83 (m, 2H, 3-H_A + 5-H_A), 1.88 (d, $^3J_{P2H}$ = 12.8 Hz, 3H, CH_3) 1.72 (apparent d of quintets, $^2J_{HH}$ = 13.2 Hz, $^3J_{HH}$ = 3.4 Hz, 1H, 4-H_A), 1.64-1.59 (m, 1H, 5-H_B), 1.52 (overlapping, d, $^3J_{PH}$ = 22 Hz, 1H, 9-H), 1.52 (d, $^3J_{P1H}$ = 12.6 Hz, 9H, ^{t}Bu at P1), 1.51-1.46 (overlapping, m, 1H, 7-H_A), 1.43-1.30 (m, 4H, 3-H_B + 4-H_B + 7-H_B + 8-H_B), 1.37 (d, $^3J_{P1H}$ = 11.8 Hz, 9H, ^{t}Bu at P1), 1.22-1.06 (m, 2H, 2-H + 6-H), 1.13 (d, $^3J_{P2H}$ = 13.9 Hz, 9H, ^{t}Bu at P2), 0.84 (d, $^3J_{P2H}$ = 10.0 Hz, 3H, CH_3). ^{13}C{^{1}H} NMR (126 MHz, C_6D_6): δ 63.60 (dd, $^2J_{P2C}$ = 11.9 Hz, $^2J_{P1C}$ = 5.0 Hz, 1-C), 50.14 (dd, $^1J_{P2C}$ = 23.6 Hz, $^3J_{P1C}$ = 1.3 Hz, 10-C), 41.05 (dd, $^2J_{P1C}$ = 7.5 Hz, $^3J_{P2C}$ = 2.0 Hz, 2-C), 36.37-36.31 (m, overlapping 5-C + 6-C), 35.89 (dd, $^1J_{P1C}$ = 19.1 Hz, $^3J_{P2C}$ = 3.3 Hz, *C*(CH_3)$_3$ at P1), 35.28 (dd, $^2J_{P2C}$ = 13.7 Hz, $^2J_{P1C}$ = 1.4 Hz, 9-C), 34.91 (dd, $^1J_{P1C}$ = 12.0 Hz, $^3J_{P2C}$ = 4.7 Hz, *C*(CH_3)$_3$ at P1), 33.88 (s, 3-C), 33.14 (dd, $^2J_{P1C}$ = 4.9 Hz, $^4J_{P2C}$ = 1.2 Hz, C(*C*H_3)$_3$ at P1), 32.55 (dd, $^1J_{P2C}$ = 10.0 Hz, $^3J_{P1C}$ = 3.4 Hz, *C*(CH_3)$_3$ at P2), 29.69 (dd, $^2J_{P1C}$ = 3.4 Hz, $^4J_{P1C}$ = 1.1 Hz, C(*C*H_3)$_3$ at P1), 29.44 (d, $^2J_{P2C}$ = 4.6 Hz, CH_3), 28.72 (dd, $^1J_{P1C}$ = 19.5 Hz, $^3J_{P2C}$ = 1.5 Hz, 7-C), 27.49 (dd, $^1J_{P2C}$ = ca. 18 Hz, $^3J_{P1C}$ = 1.0 Hz, 8-C, overlapping with ^{t}Bu), 27.40 (dd, $^2J_{P2C}$ = 3.8 Hz, $^4J_{P1C}$ = 1.4 Hz, C(*C*H_3)$_3$ at P2), 26.72 (d,

$^3J_{P1C}$ = 1.5 Hz, 4-C), 21.40 (dd, $^2J_{P2C}$ = 3.6 Hz, $^4J_{P1C}$ = 1.0 Hz, CH_3). $^{31}P\{^1H\}$ NMR (202 MHz, C_6D_6): δ 66.28 (d, $^2J_{P1P2}$ = 370.0 Hz), 23.74 (d, $^2J_{P1P2}$ = 370.0 Hz).

Crystallography. Intensity data were collected at 293 K with an Oxford Diffraction Xcalibur 3 system using ω-scans and Mo-Kα (λ = 0.71073 Å). CCD data were extracted and integrated using Crysalis RED[2]. The structures were solved using direct methods and refined by full-matrix least-squares calculations on F^2 using SHELXTL 5.1[3]. Non-H atoms were refined with anisotropic displacement parameters. Hydrogen atoms were constrained to parent sites, using a riding model.

Table 1. Crystal data collection and refinement details for compounds **3-*anti***, **4 and 21**.

	**3-*anti***	**4**	**21**
formula	$C_{31}H_{55}IrOP_2$	$C_{30}H_{51}IrP_2$	$C_{24}H_{46}ClIrP_2$
Fw	697.89	665.85	624.20
crystal system	monoclinic	triclinic	monoclinic
space group	$P2_1/c$	P-1	$P2_1/c$
color	colorless	red	yellow
a/Å	17.0030(5)	10.264(5)	11.4108(10)
b/Å	20.0591(5)	16.311(5)	13.6574(12)
c/Å	9.4403(3)	19.425(5)	17.7933(15)
α/deg	90	103.753(5)	90
β/deg	102.986(3)	105.008(5)	104.097(8)
γ/deg	90	93.129(5)	90
V/Å^3	3137.42(16)	3027.6(19)	2689.4(4)
temperature, K	293	293	293
Z	4	4	4
D_{calcd}/g cm^{-3}	1.306	1.461	1.542
μ/mm^{-1}	4.379	4.531	5.191
θ/ range/deg	2.46-29.01	2.54-28.39	2.44-28.99
no. reflns collected	23942	65691	30088
no. of unique reflns	7396	14034	6485
$R(F)$ (I>2σ(I))[a]	0.0572	0.0350	0.0260
$wR2(F^2)$ (all data)[b]	0.1144	0.0915	0.0788
S[c]	1.056	1.222	1.269
R_{int}	0.0600	0.0463	0.0324

[a] $R = \Sigma(|F_o| - |F_c|)/\Sigma|F_o|$. [b] $wR2 = [\Sigma w(F_o^2 - F_c^2)^2/\Sigma(F_o^2)^2]^{1/2}$. [c] $S = [\Sigma w\, F_o^2 - F_c^2)^2/(n-p)]^{1/2}$.

2. Computational part

Summary of Additional Computational Results

All Density Functonal Theory (DFT) calculations were carried out with Jaguar 7.6 program package by Schrödinger LLC[4]. For geometry optimization, solvation energy and frequency calculations, Becke´s three-parameter hybrid functional and the LYP correlation functional (B3LYP)[5,6] was used with LACVP** level core potential and basis set, while single point energy corrections were performed with the M06[7], M06-L[8] or B3LYP-D3[9] functional using the LACV3P**++ basis set which, as suggested by Martin[10], was augmented with two f-polarization functions on Ir. Frequency calculations at the same level were performed on the optimized geometries to verify that the geometries correspond to minima (no imaginary frequency) o transition states (one imaginary frequency) and to provide the thermochemical data at different temperatures (298 K, 393 K and 413 K), which include entropy contributions. Single-point solvation energies were also calculated using the Poisson-Boltzmann reactive field implemented in Jaguar 7.6 (PBF)[11] with standard parameters for benzene. The Gibbs free energies were defined as following equation:

$G = E(\text{B3PLYP-D3/LACV3P**++2f on Ir}) + G_{solv} + ZPE + H_{298} - TS_{298} + 1.9$ [concentration correction to the free energy of solvation from M(g) → M(aq) to atm(g) → M(aq)].

Choice of functional: M06, M06-L versus B3LYP-D3

Since DFT studies for iridium complex with PCP pincer ligands with different density functionals have been reported[12], we carried out comparable calculations using these three functionals (M06, M06-L and B3LYP-D3). In general all the calculations follow the send trend and agree with the experimental data, however the agreement with the experimental observations is a bit larger with the density functional B3LYP-D3. Therefore, we believe that the results from B3LYP-3/ LACV3P**++ method are more reliable.

Hydrogenation of 4. DFT calculations on the reaction of complex **4** with dihydrogen confirmed the experimental observation that complexes **5** and **6** are in equilibrium depending on the temperature and hydrogen-pressure used in the reaction (Figure 2). The formation of complex **6** occurs, through H_2– addition to complex **4** forming the Ir(III) complex **5**. After this, Ir–H insertion into the double bond of the pincer ligand leads to the intermediate **8**. This Ir(III) complex undergoes an oxidative addition to form the experimentally observed Ir(V) complex **6**. The activation energy barrier for this conversion is calculated to be 20.5 kcal/mol *via* **TS8-5**.The calculations showed that complex **6** is more stable than intermediate **8** (under dihydrogen atmosphere). Depending on the choice of functional to calculate the electronic energy, (a) M06, (b) M06-L, or (c) B3LYP-D3, they all favor formation of complex **6** vs complex **8**, however the preference for **6** over **8** is a bit larger with B3LYP-D3 in agreement with the experimental observations. The equilibrium between **5** and **6** appears to be most accurate with B3LYP-D3 followed by M06 and M06-L (Figure S1).

.

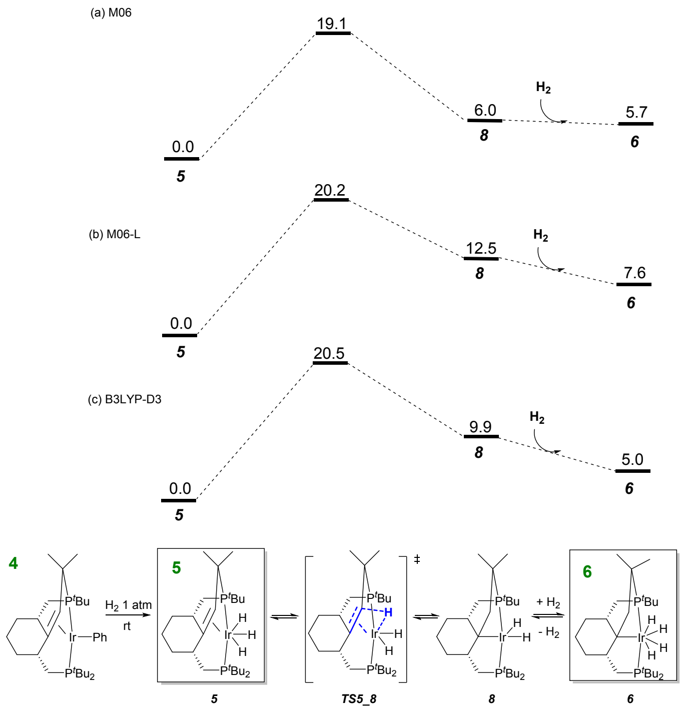

Figure S1. Profile of the calculated relative G (kcal/mol) for the formation of mixture of complexes **5** and **6** from complex **4** at 25 ºC (a) M06 functional, (b) M06-L, (c) B3LYP-D3

Different mechanistic scenarios were considered for the formation of complex **7** at the experimental conditions (5 atm of H_2, 140 ºC) (Figure S2). The green and black paths of Figure S2 shows how complex **5**, *via* intermediate **8** undergoes an α-alkyl elimination to form carbene **9**. The activation barrier is calculated at 31.4 kcal/mol *via* transition state **TS8-9** (See Figure S3 for a more detailed picture of transition states). Complex **9** proceeds through an α-insertion to form intermediate **10**, which under hydrogen atmosphere conditions undergoes a H_2 addition that finally forms the more stable Ir(V) complex **11**. The transition state **TS9-10** is calculated at 25.8 kcal/mol on the free energy surface. This step is followed by C–H reductive elimination from Ir–C_{sp3} and Ir-H, which leads to formation of a new C_{sp3}–H bond in the pincer ligand, a phosphorus-bound *tert*-butyl group. The free energy barrier *via* transition state **TS11-12** is calculated to be 11.8 kcal/mol. Finally the process finishes with an oxidative addition of one dihydrogen molecule leading to Ir(V)H_4 complex **7**. The overall activation free energy corresponds to the C–C bond cleavage step (31.4 kcal/mol). Note that the results that are reported here have been calculated at 1 atm of H_2, and the experimental were carried out at 5 atm. The effect of the pressure can affect the total values around $\approx$1 kcal. Other mechanistic proposals are evaluated in Figure S2 for the formation of complex **7**: (a) through complex **22**, and (b) through complex **13**. (A) Carbene **9** can undergo a reductive elimination forming the C–H bond and generating Ir complex **22**. This transition state has a free energy barrier of 41.0 kcal/mol. By comparing with the green α-insertion pathway, it is clear that this path is essentially impossible under the experimental conditions, and the reaction favorably goes *via* transition state **TS9-10**. (B) Through complex **13** (figure 2b), there are two possible alternative pathways. From

complex **8**, a reductive elimination step can occur to generate intermediate **13** with a plausible free energy barrier for the transition state (23.7 kcal/mol), followed by a C–C oxidative addition that leads to complex **10**. The free energy of the transition state **TS13-10** is 53.6 kcal/mol. Alternatively, from complex **13**, an oxidative addition of a dihydrogen molecule to Ir can occur to form complex **14** which undergoes C–C oxidative addition generating complex **11**. The activation energy barrier for this pathway is even higher at 62.9 kcal/mol *via* **TS14-11**. It can clearly be concluded, similarly to the statement above, that the proposal the green α-insertion pathway is the most plausible pathway due to the significantly lower free energy barriers, (**TS9-8** : 31.4 kcal/mol) *vs* (**TS13-10** : 53.6 kcal/mol) and (**TS14-11** : 62.9 kcal/mol).

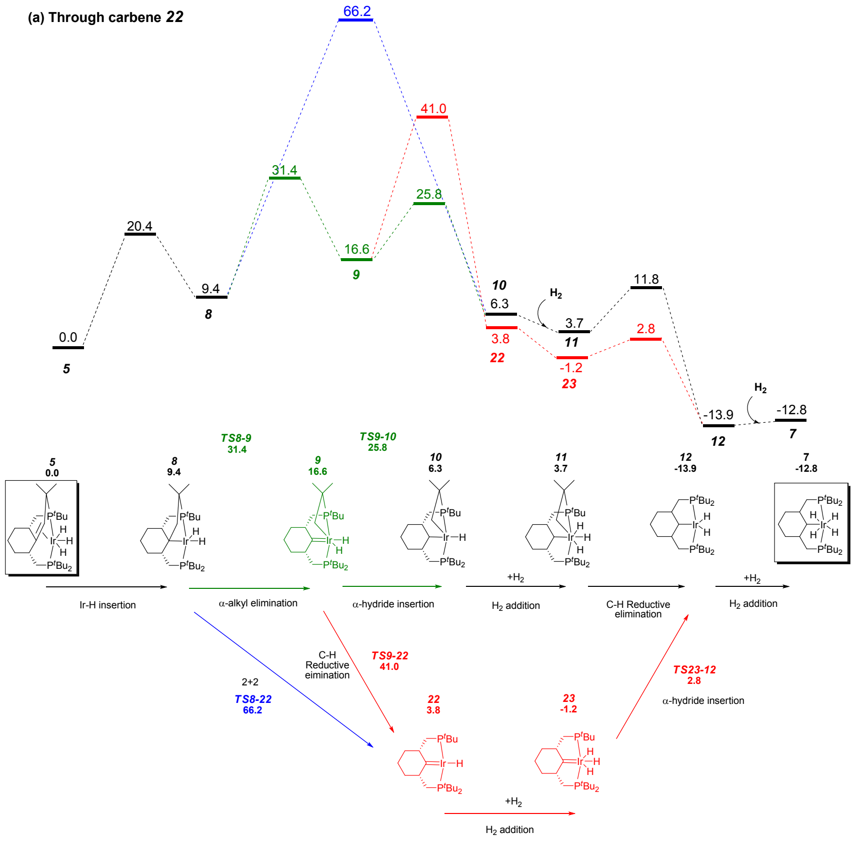

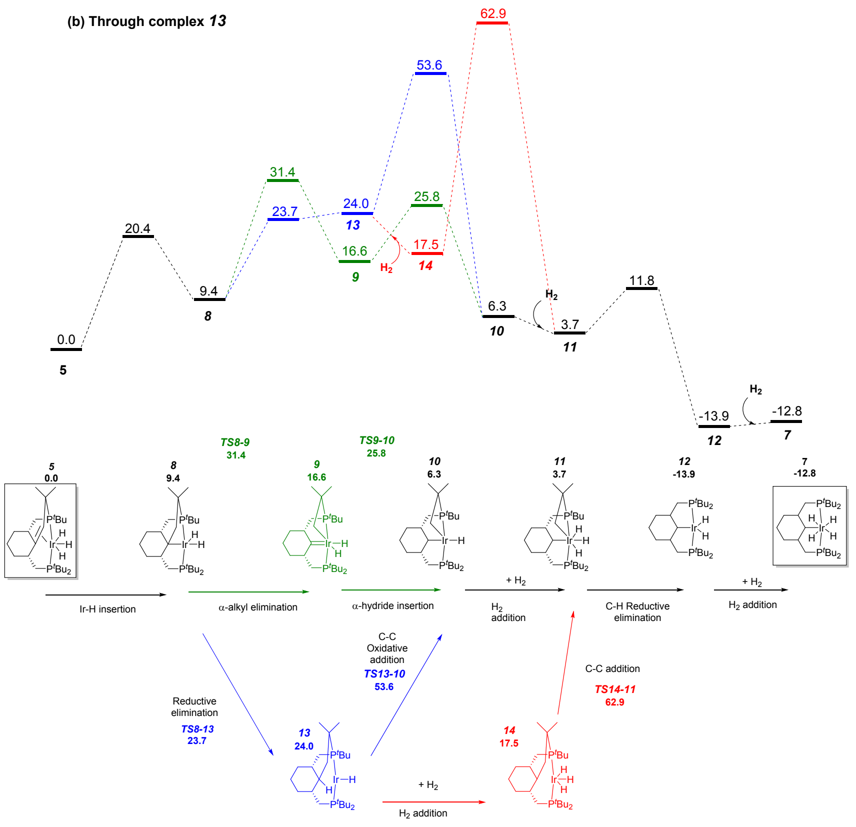

Figure S2. Proposed pathways for the formation of complex **7** from complex **5**: (a) through carbene **22**; (b) through complex **13**. The relative Gibbs free energies are given in kcal/mol. In both a) and b) the lowest energy pathway is marked by green and black and involves formation of carbene **9**.

Figure. S3. Optimized structures for intermediate **9/10** and transition states **TS8-9/TS9-10**, **TS8-13/TS13-10** and **TS14-11/TS9-22/TS8-22**. Relative energies are given in kcal/mol and distances in Å.

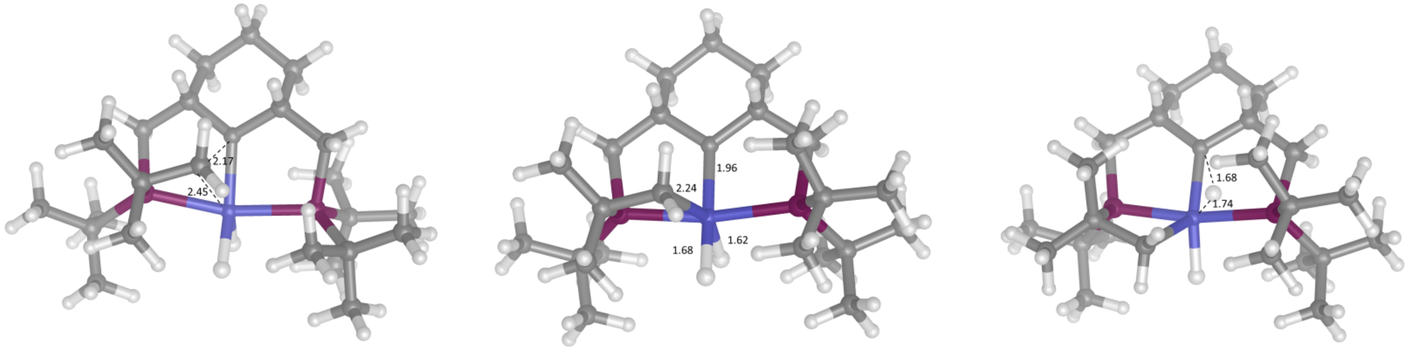

TS8-9 (31.4)   ***9*** (16.6)   ***TS9-10*** (25.8)

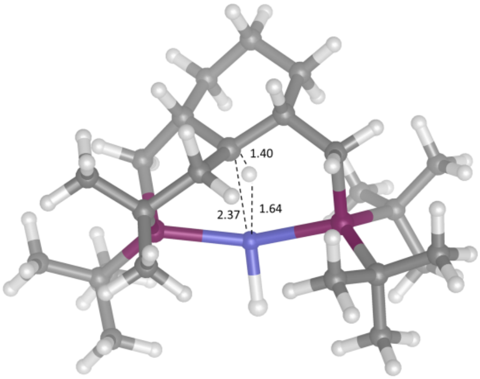

TS8-13
(23.7)

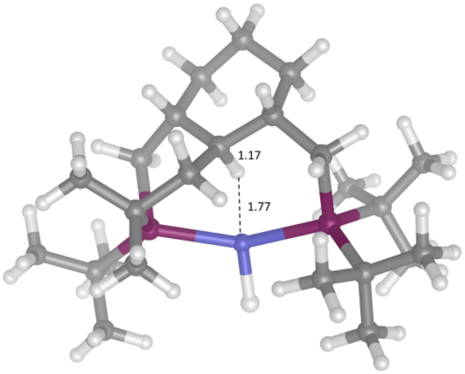

13
(24.0)

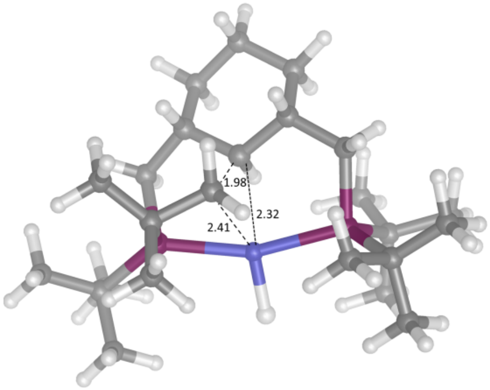

TS13-10
(53.6)

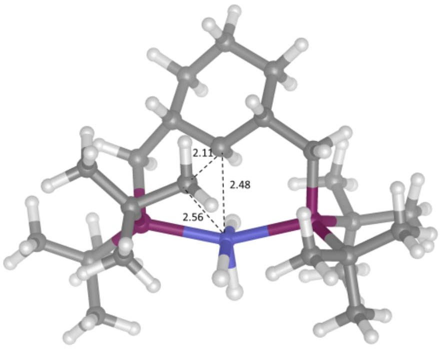

TS14-11
(62.9)

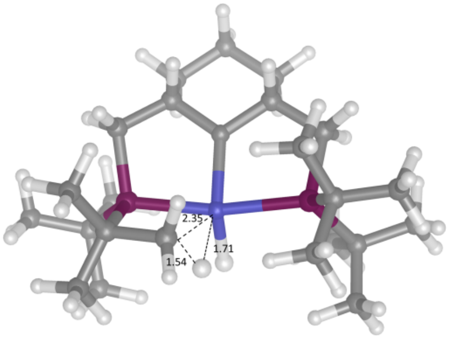

TS9-22
(41.0)

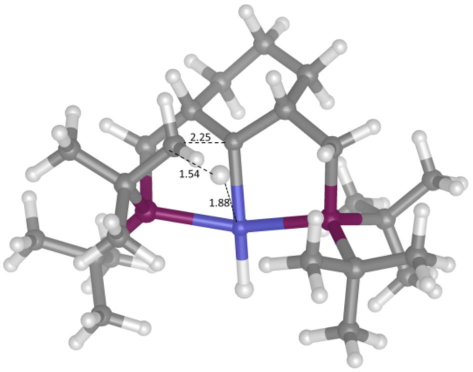

TS8-22
(66.2)

Carbon-carbon bond formation. As mentioned, it was found experimentally that the formation of complex **7** is reversible, proceeding in one direction or other depending on the reaction conditions. In the presence of a hydrogen atmosphere, complex **7** is obtained quantitatively, and in the presence of a hydrogen acceptor, such as *tert*-butylethylene, the forward reaction occurs leading to the olefin complex **15** (Figure S7). Complex **12** proceeds through a C−H oxidative addition process forming Ir(V) complex **11**. The free energy barrier via transition state **TS12-11** is calculated to be 25.6 kcal/mol. This step is the only one that is feasible for both conditions (under H_2 and in the presence of *tert*-butylethylene). In the reaction with *tert*-butylethylene, complex **11** goes through reductive elimination followed by α-elimination forming iridium complex carbene **9**. The activation barrier is calculated at 22.6 kcal/mol via transition state **TS10-9.** Complex **9** *via* C−C coupling transition state **TS9-8** (28.0 kcal/mol) leads to the formation of intermediate **8**, which proceeds through β-elimination and finally reductive elimination to

generate the more stable complex **15**. The activation barrier is calculated at 17.0 kcal/mol *via* transition state **TS8-5**. In the reaction with *tert*-butylethylene, the overall activation energy barrier corresponds to the C–C bond formation step (**TS9-8**) that is 28.0 kcal/mol. The forward reaction is not possible under hydrogen pressure due to the higher energy barriers. Under hydrogen pressure, the formation of complex **5** needs to cross over a free energy barrier of 45.7 kcal/mol (**TS9-8**), that makes this C–C coupling step unfavorable under these conditions.

Figures S4-S7 list the energy profiles calculated using various functionals ((a) M06 functional, (b) M06-L, (c) B3LYP-D3)

(a) M06

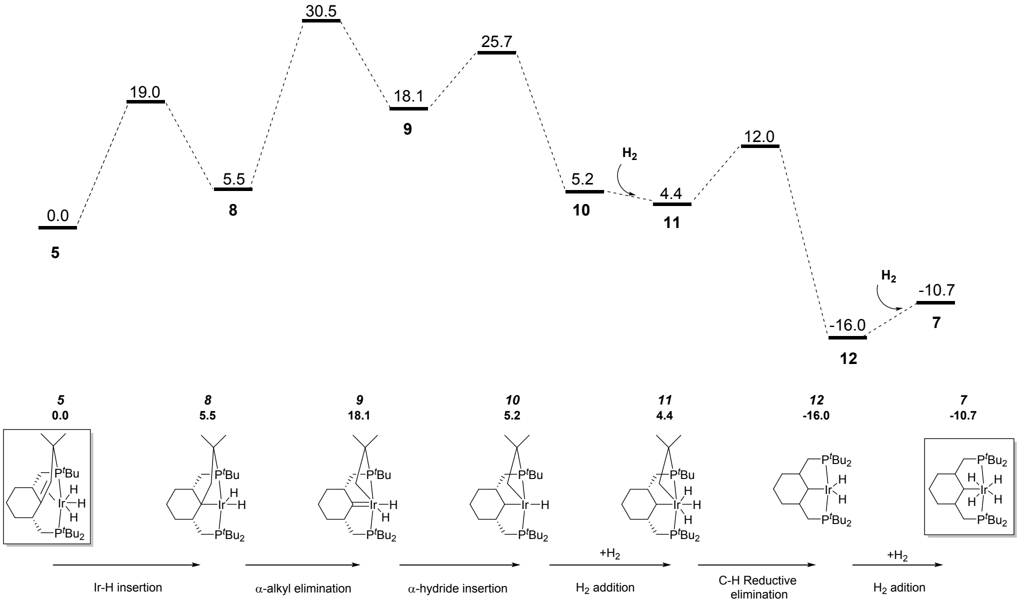

(b) M06-L

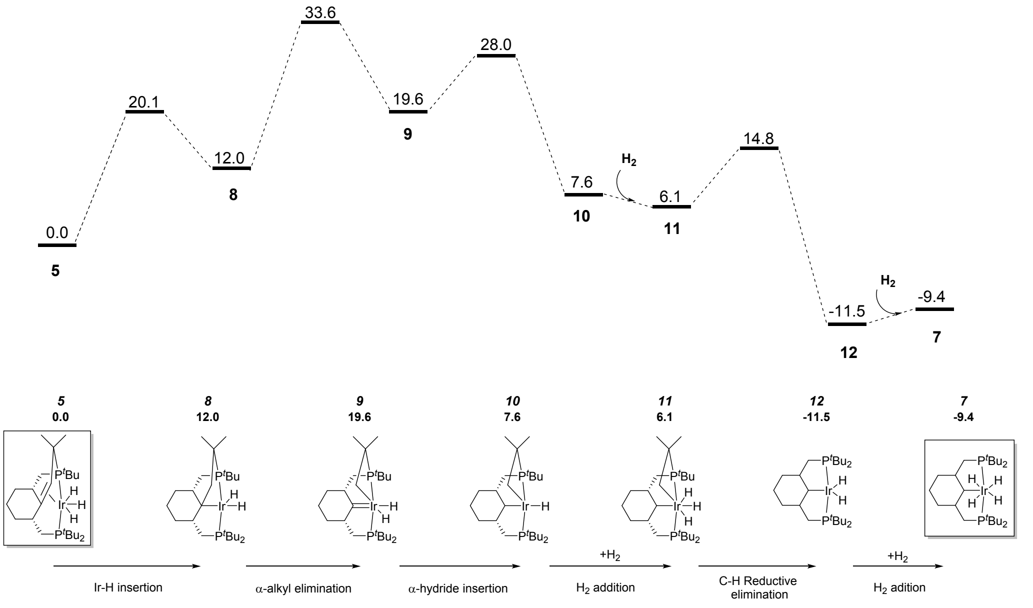

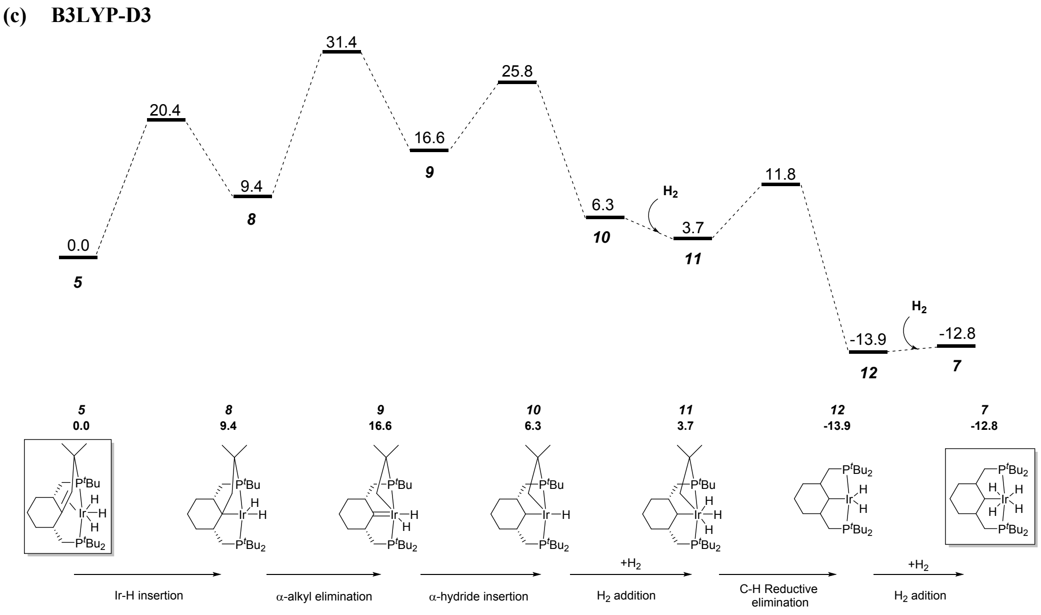

Figure S4. Profiles of the calculated relative G (kcal/mol) for the formation of complex **7** from complex **5** at 140 ºC: (a) M06 functional, (b) M06-L, (c) B3LYP-D3.

(a) M06

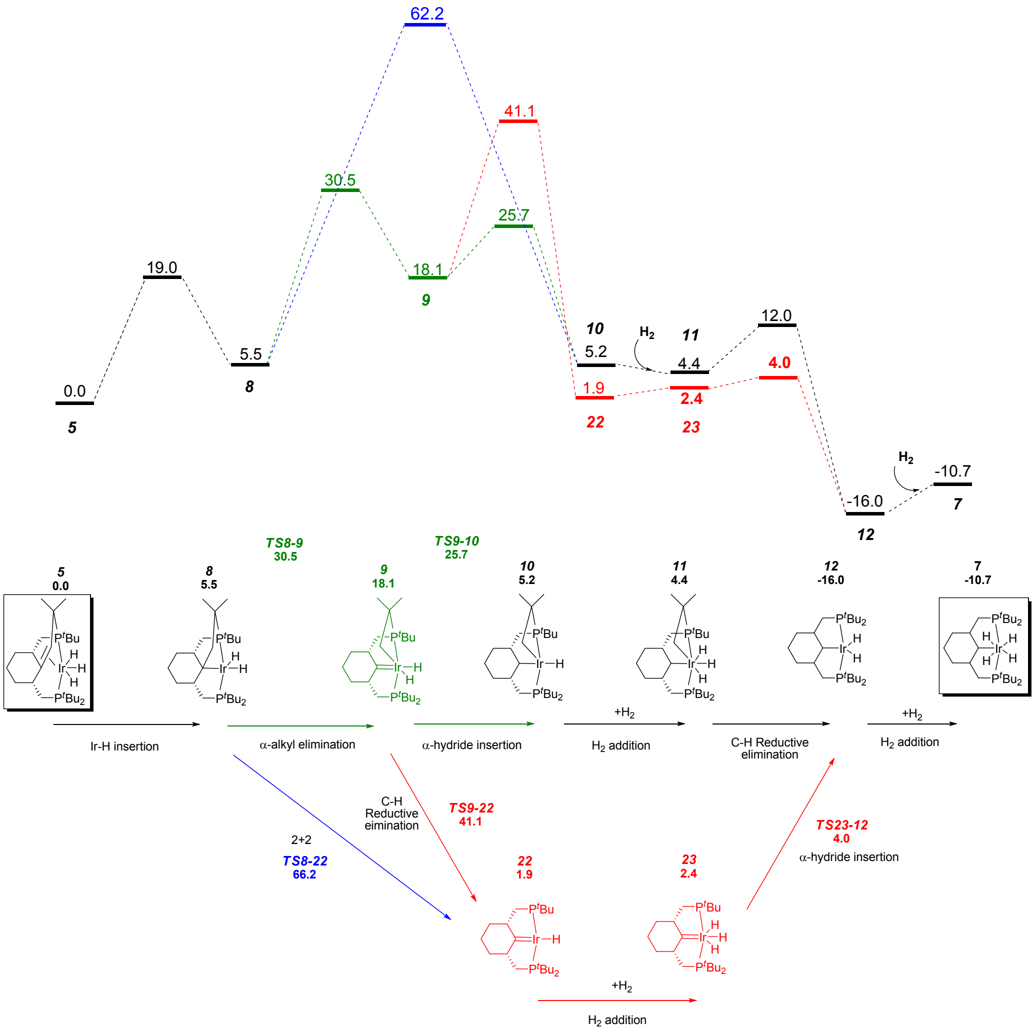

62.2
41.1
30.5
25.7
19.0
18.1
9
10
5.2
H2
11
4.4
12.0
4.0
5.5
8
0.0
5
1.9
2.4
22
23
H2
-10.7
7
-16.0
12
TS8-9
30.5
TS9-10
25.7
5
0.0
8
5.5
9
18.1
10
5.2
11
4.4
12
-16.0
7
-10.7
Ir-H insertion
α-alkyl elimination
α-hydride insertion
+H2
H2 addition
C-H Reductive elimination
+H2
H2 addition
C-H Reductive eimination
TS9-22
41.1
2+2
TS8-22
66.2
22
1.9
23
2.4
TS23-12
4.0
α-hydride insertion
+H2
H2 addition

(b) M06-L

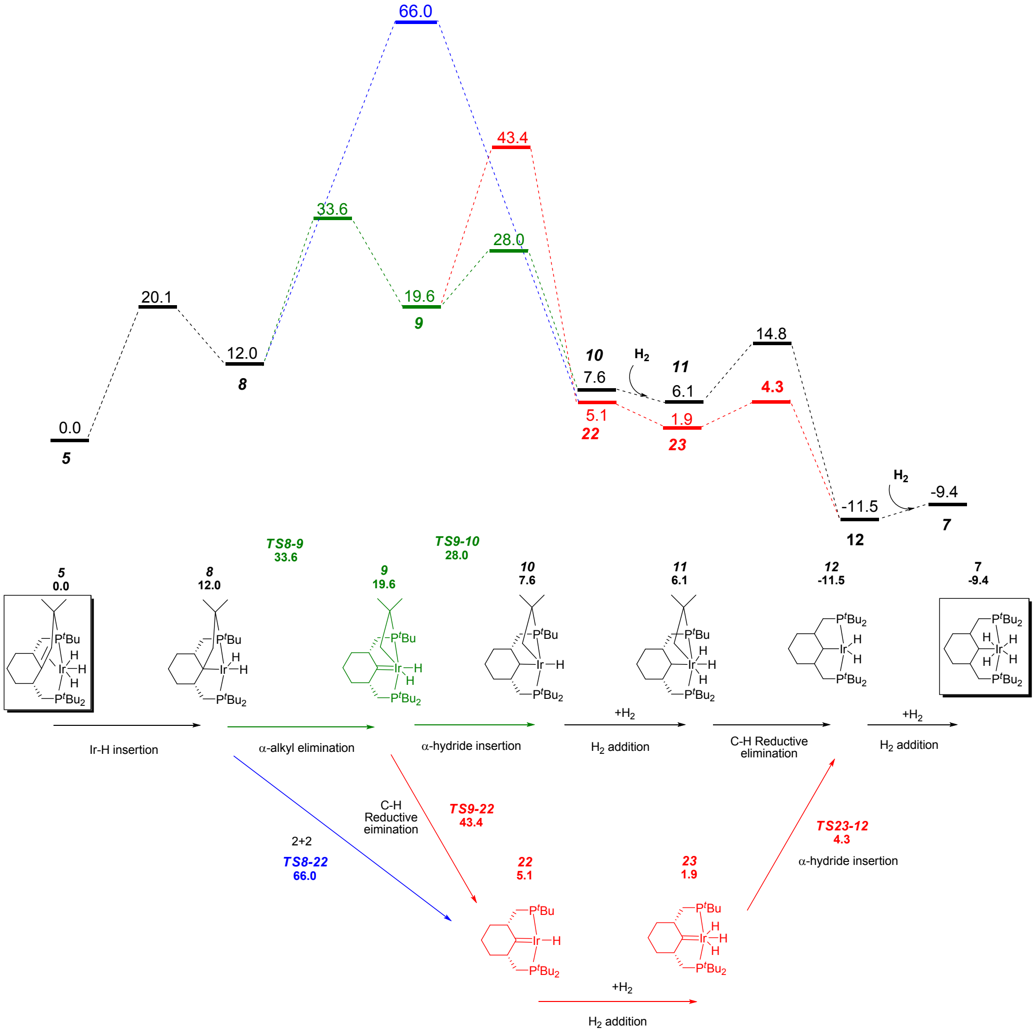

66.0
43.4
33.6
28.0
20.1
19.6
9
12.0
8
10
7.6
H2
11
6.1
14.8
4.3
5.1
22
1.9
23
0.0
5
H2
-11.5
-9.4
7
12
TS8-9
33.6
TS9-10
28.0
5
0.0
8
12.0
9
19.6
10
7.6
11
6.1
12
-11.5
7
-9.4
Ir-H insertion
α-alkyl elimination
α-hydride insertion
+H2
H2 addition
C-H Reductive elimination
+H2
H2 addition
2+2
TS8-22
66.0
C-H Reductive eimination
TS9-22
43.4
22
5.1
23
1.9
TS23-12
4.3
α-hydride insertion
+H2
H2 addition

(c) B3LYP-D3

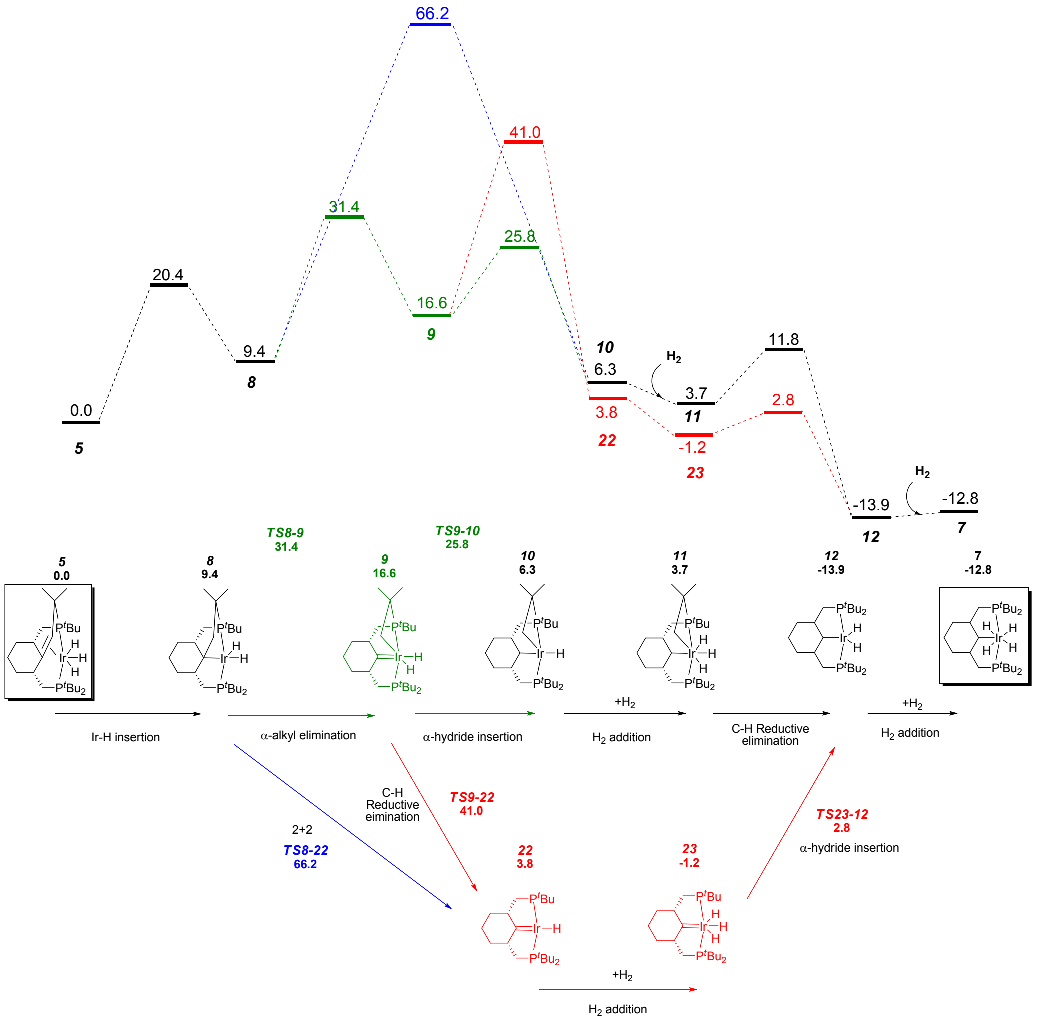

Figure S5. Proposed pathways for the formation of complex **7** from complex **5** through carbene **22**; The relative Gibbs free energies are given in kcal/mol at 140 ºC : (a) M06 functional, (b) M06-L, (c) B3LYP-D3.

(a) M06

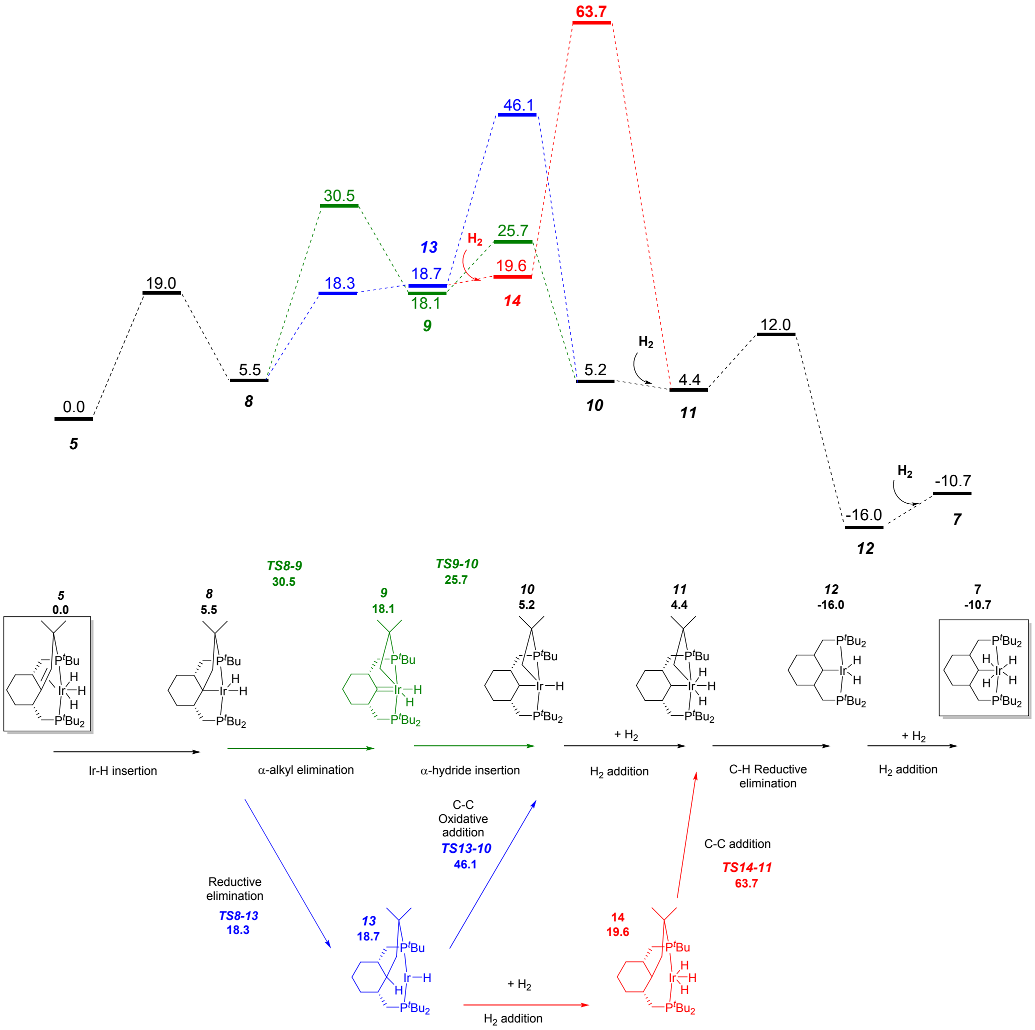

63.7
46.1
30.5
25.7
13
18.7
18.1
9
19.6
14
19.0
5.5
8
0.0
5
18.3
5.2
10
4.4
11
12.0
-16.0
12
-10.7
7
TS8-9 30.5
TS9-10 25.7
Ir-H insertion
α-alkyl elimination
α-hydride insertion
+ H2
H2 addition
C-H Reductive elimination
Reductive elimination
TS8-13 18.3
C-C Oxidative addition
TS13-10 46.1
C-C addition
TS14-11 63.7

(b) M06-L

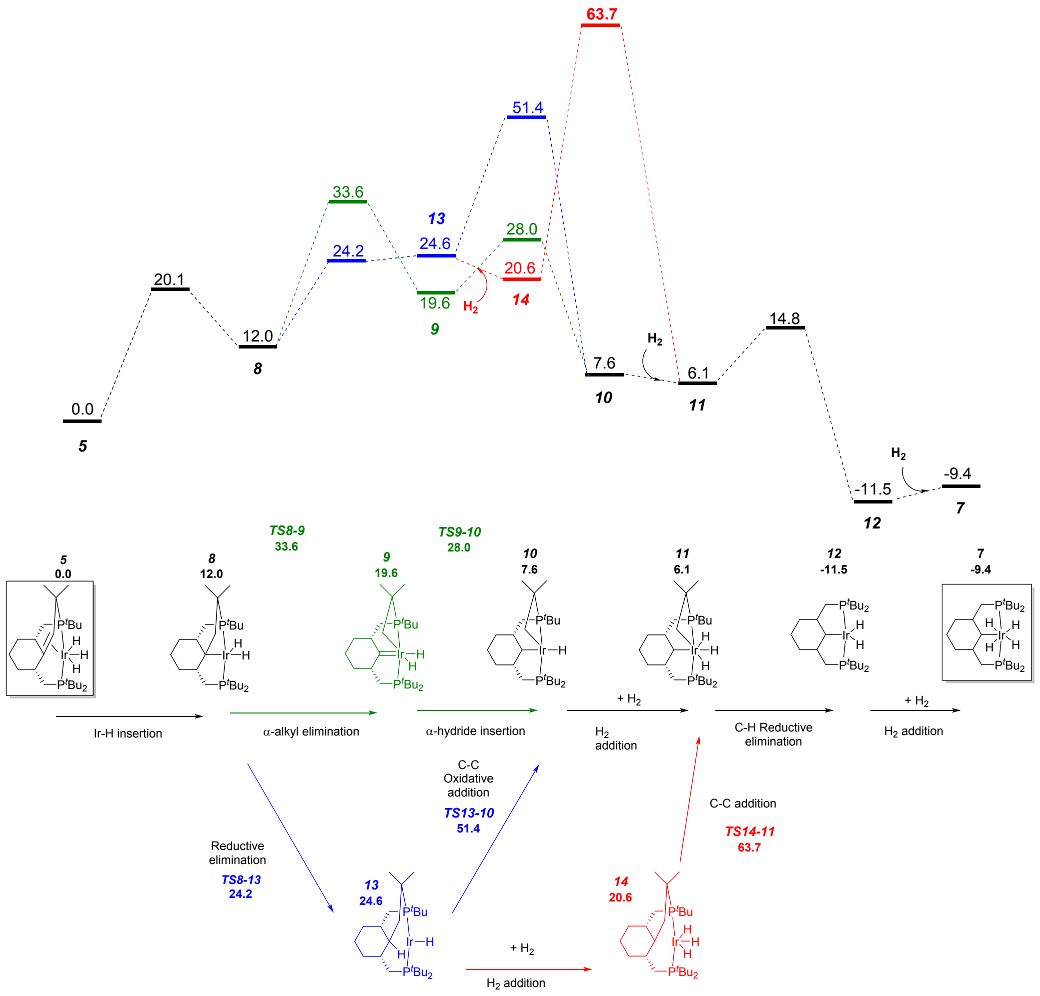

63.7
51.4
33.6
13
24.2
24.6
28.0
20.6
19.6
H_2
14
20.1
12.0
9
8
14.8
H_2
7.6
6.1
10
11
0.0
5
H_2
-11.5
-9.4
12
7
TS8-9
33.6
TS9-10
28.0
5
0.0
8
12.0
9
19.6
10
7.6
11
6.1
12
-11.5
7
-9.4
Ir-H insertion
α-alkyl elimination
α-hydride insertion
+ H_2
H_2 addition
C-H Reductive elimination
+ H_2
H_2 addition
C-C Oxidative addition
TS13-10
51.4
C-C addition
TS14-11
63.7
Reductive elimination
TS8-13
24.2
13
24.6
14
20.6
+ H_2
H_2 addition

(c) B3LYP-D3

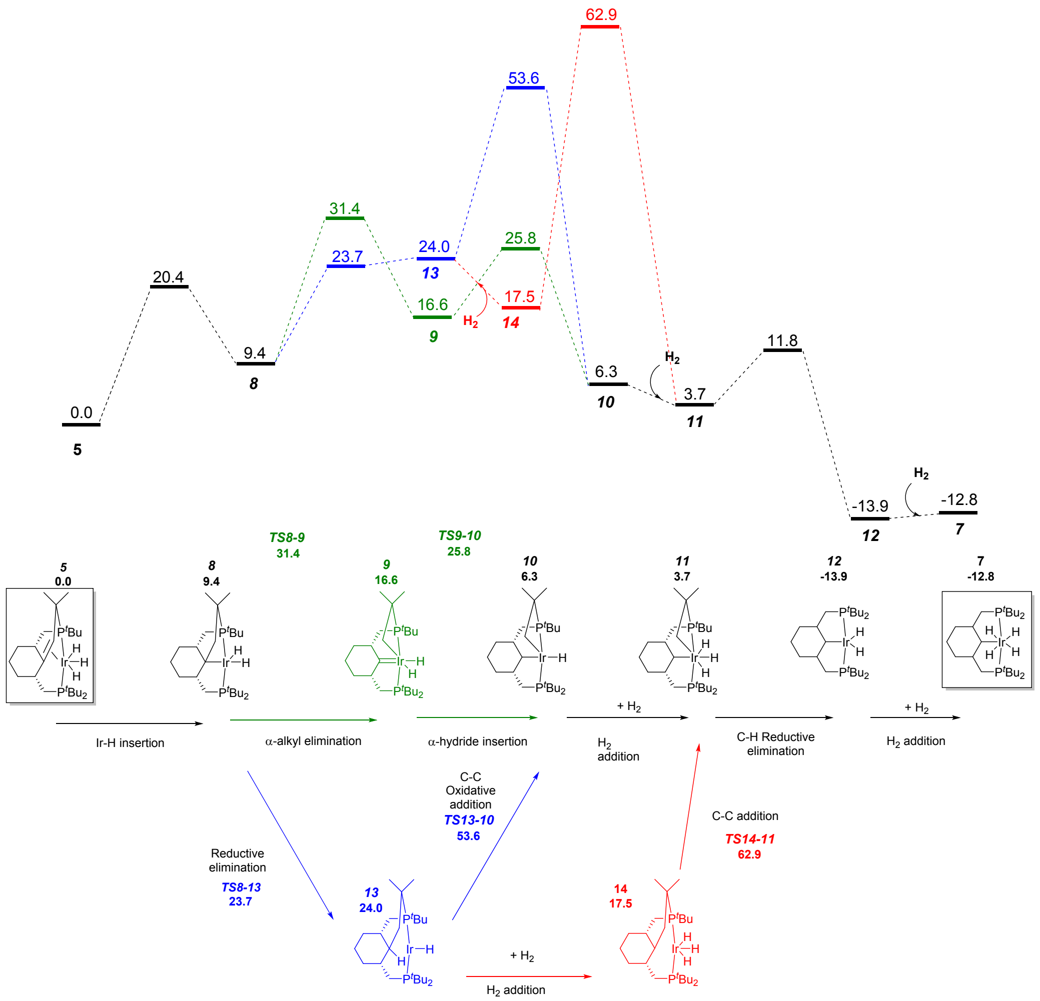

Figure S6. Proposed pathways for the formation of complex **7** from complex **5** through complex **13** at 140 ºC. The relative Gibbs free energies are given in kcal/mol. : (a) M06 functional, (b) M06-L, (c) B3LYP-D3.

(a) M06

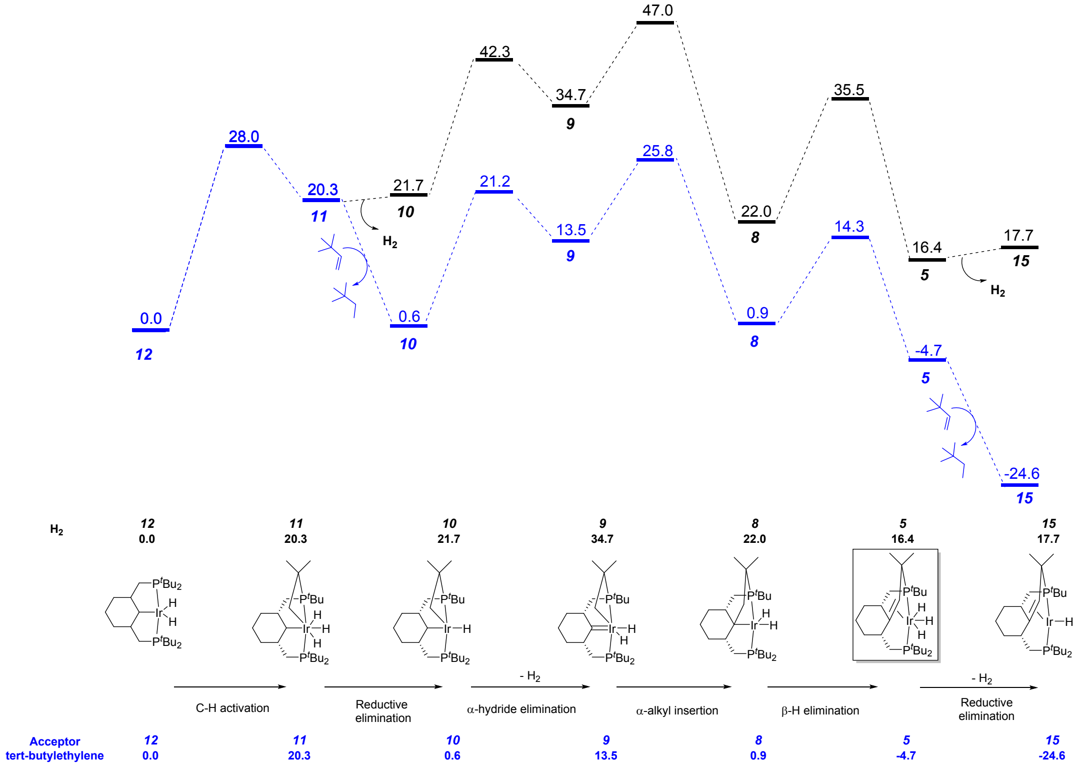
47.0
42.3
34.7
9
35.5
28.0
20.3
11
21.7
10
H2
21.2
25.8
13.5
9
22.0
8
14.3
16.4
5
17.7
15
H2
0.0
12
0.6
10
0.9
8
-4.7
5
-24.6
15
H2
12
0.0
11
20.3
10
21.7
9
34.7
8
22.0
5
16.4
15
17.7
C-H activation
Reductive elimination
- H2
α-hydride elimination
α-alkyl insertion
β-H elimination
- H2
Reductive elimination
Acceptor
tert-butylethylene
12
0.0
11
20.3
10
0.6
9
13.5
8
0.9
5
-4.7
15
-24.6

(b) M06-L

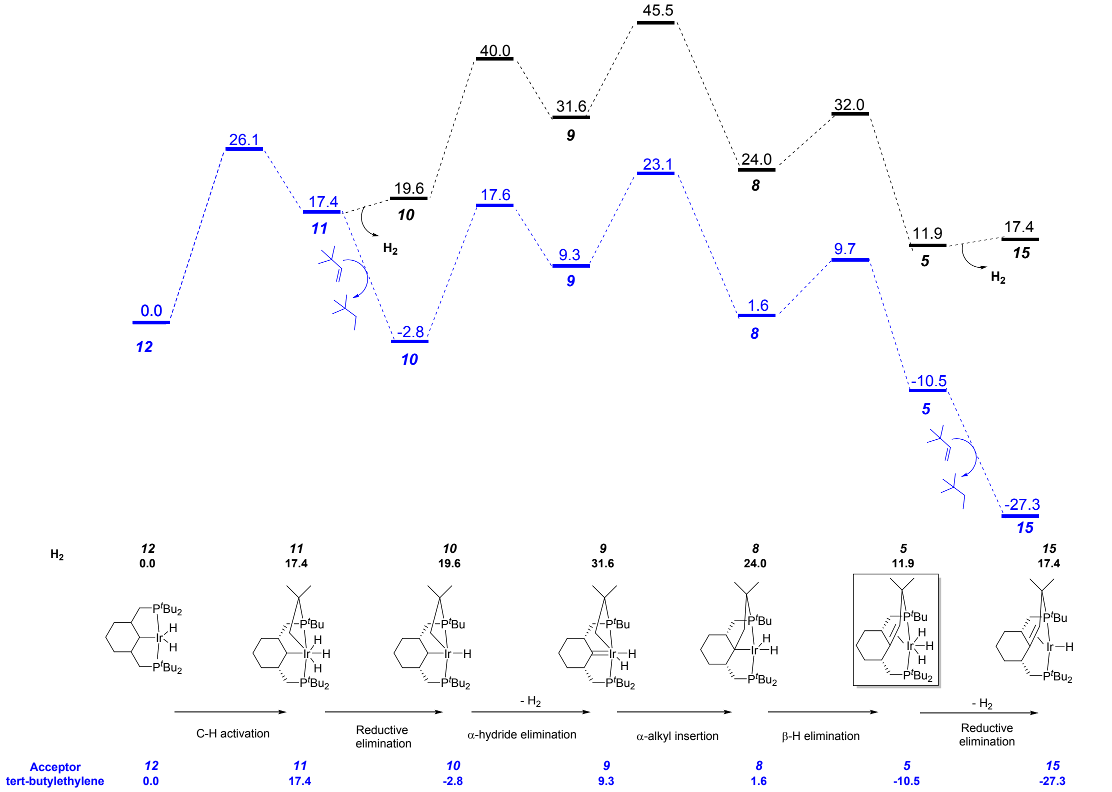
45.5
40.0
31.6
9
32.0
26.1
17.4
11
19.6
10
H2
17.6
23.1
24.0
8
9.3
9
9.7
11.9
5
17.4
15
H2
0.0
12
-2.8
10
1.6
8
-10.5
5
-27.3
15
H2
12
0.0
11
17.4
10
19.6
9
31.6
8
24.0
5
11.9
15
17.4
C-H activation
Reductive elimination
- H2
α-hydride elimination
α-alkyl insertion
β-H elimination
- H2
Reductive elimination
Acceptor
tert-butylethylene
12
0.0
11
17.4
10
-2.8
9
9.3
8
1.6
5
-10.5
15
-27.3

(c) B3LYP-D3

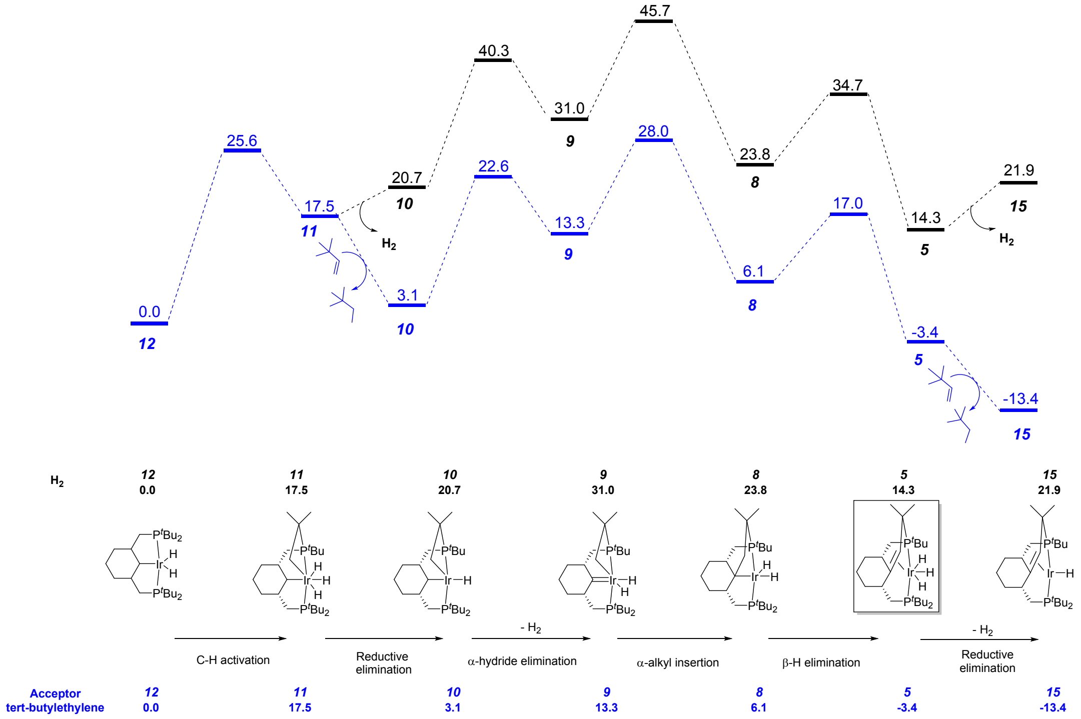

Figure S7. Profile of the calculated relative G (kcal/mol) for the forward reaction from complex **12** at 120 ºC: (a) M06 functional, (b) M06-L, (c) B3LYP-D3.

Reductive elimination of benzene from complex 2. DFT calculations on the reaction of complex **2** with dihydrogen confirmed the experimental data (Figure 8-9). The formation of complex **12** occurs, through reductive elimination of benzene from complex **2** (detailed calculations were done to both isomers: *syn* and *anti* isomers) forming the Ir(I) complex **24.** (**TS2-24** ***syn*** : 15.1 kcal/mol) and (**TS2-24** ***anti*** : 16.3 kcal/mol). After this, H_2– addition leads to the experimentally observed intermediate **12**.

It can clearly be concluded, similarly to the statement above, that the DFT calculations agree very well with the experimental data. (**AG experimental** : 14.0 kcal/mol) *vs* (**AG calculated by DFT**: ***anti***: 16.3 kcal/mol and ***syn*** : 15.1 kcal/mol).

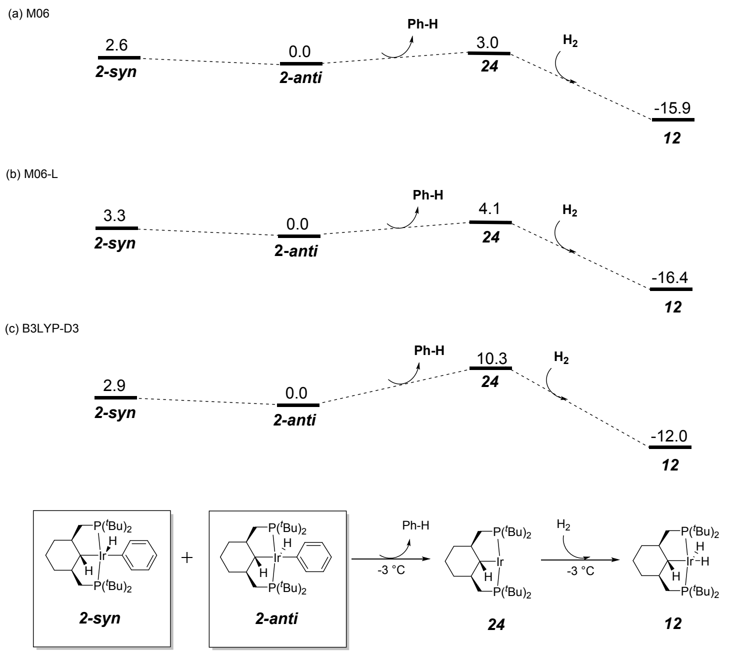

Figure S8. Profile of the calculated relative G (kcal/mol) at -3 °C for the benzene reduction of **2-*syn*** and **2-*anti***. (a) M06 functional, (b) M06-L, (c) B3LYP-D3.

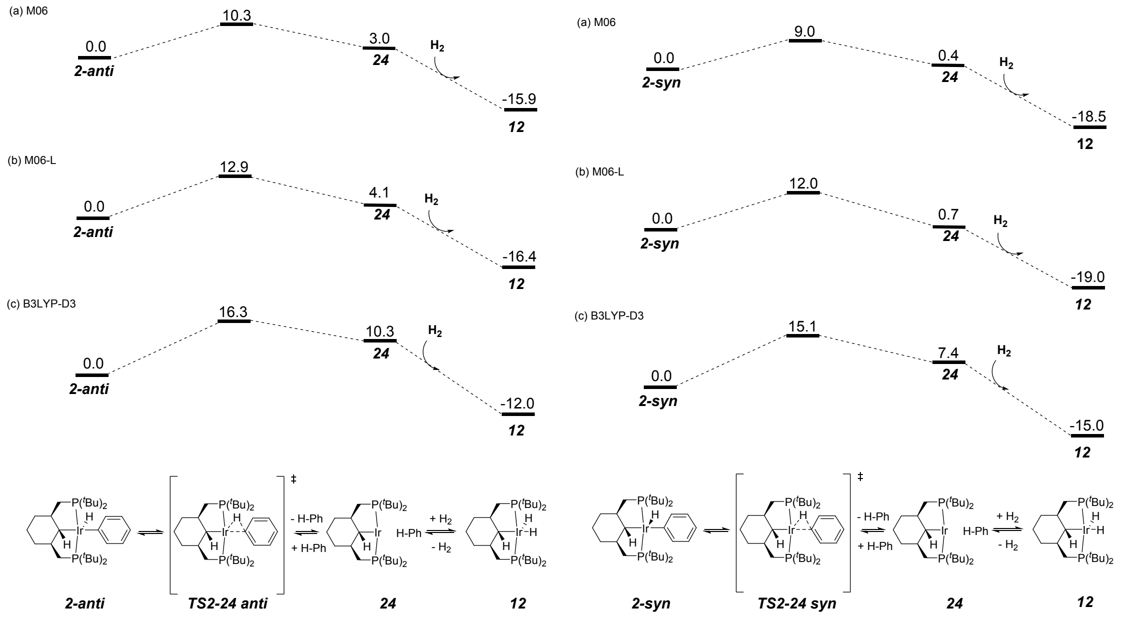

Figure S9. Profiles of the calculated relative G (kcal/mol) at -3 °C for the benzene reduction of **2-*syn*** and **2-*anti***. (a) M06 functional, (b) M06-L, (c) B3LYP-D3.

NBO studies of carbenes: With the aim of evaluating the nature of the iridium carbene complexes, NBO calculations[13] were made. The results are given in Table S1. The NBO analysis data exhibit typical scheme for the Fischer complexes; **16** and **22** have a Ir-$C_{carbene}$ σ and π bond, while **9** has only σ bond. The Ir-$C_{carbene}$ σ bond of complexes **16** and **22** are clearly polarized towards the carbon end (only 40% and 42%, respectively, are at the iridium end) while the π bond is polarized towards the metal moiety (65% and 64%). The NBO bonding pattern suggest that the Ir-C π bond is even more polarized towards the metal moiety because the optimal Lewis structure has a iridium lone-pair d(π) orbital rather than a π bond. The calculated hybridization shows that the σ bond has mainly d character at the iridium end (65%d and 63%d), while the π bond at iridium is purely d(Ir) (97% and 94%). Note that the σ and π electrons in Schrock complexes are more polarized towards the carbon end. In the case of the carbene **9**, the calculated polarization of the Ir-$C_{carbene}$ σ bonds are more equally distributed (57%). The NBO results for the complexes show that the formal charge are between -0.04 and +0.06. This is gratifying, because typically the metal of the Schrock complexes is more positively charged and has a lower 5d population than the Fischer complexes.[14]

As a summary, all the complexes showed very different NBO results from Schrock complexes, and several similarities with Fischer complexes.

Table S1. Results of the NBO of the iridium carbene complexes **16**, **22**, **9** using B3LYP/LACVP**

Carbene	Atom				Occuppational		**Ir-C** bond				Natural Charge			
	**6s**	**5d**	**6d**	**7p**			**% Ir**	**% s**	**% p**	**% d**	**Ir**	**C1**	Pincer	**Cl/H**
**16**	0.67	8.37	0.02	0.01	σ	1.91168	39.80	35.37	0.03	64.60	-0.0465	-0.00591	+0.6372	-0.5907
					π	1.93786	65.20	2.65	0.16	97.19				
**22**	0.69	8.45	0.01	0.02	σ	1.88195	42.12	37.11	0.00	62.80	-0.1522	-0.0479	+0.4569	-0.3047
					π	1.92180	64.25	5.29	0.21	94.50				
**9**	0.57	8.35	0.02	0.01	σ	1.48548	57.09	2.40	30.87	66.73	+0.0600	-0.14317	-0.16	-0.1000

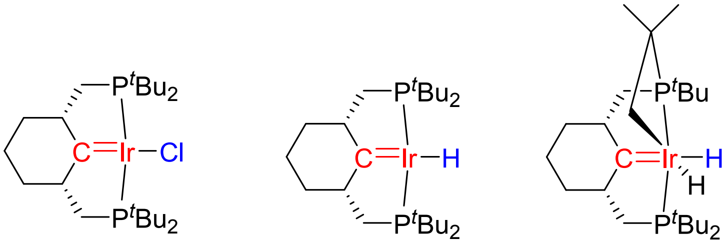

16 **22** **9**

Calculated energies for all the species in this study

Table S2. The calculated energies at B3LYP level with LAVCP** basis set and the single-point energies at M06, M06-L or B3LYP level with LAVC3P**++ basis set, respectively at 298K.

Molecules /complexes	Energy B3LYP LACVP** [hartree]	Energy M06 LACV3P**++ [hartree]	Energy M06-L LACV3P**++ [hartree]	Energy B3LYP-D3 LACV3P**++ [hartree]	Zero-pt E [kcal/mol]	Enthalpy [kcal/mol]	Entropy [cal/mol K]	Solvation E [kcal/mol]
**H_2**	-1.178539	-1.17026	-1.171479	-1.171479	6.384	2.074	31.132	0.000
***tert*-butylethylene$^-$**	-235.860939	-235.712436	-235.873665	-235.93135	103.482	5.405	82.508	-0.699
***tert*-butylethane**	-237.09896	-236.945831	-237.110185	-237.168529	118.244	5.742	84.931	-0.228
**5**	-1731.570932	-1730.990513	-1731.701294	-1731.96998	429.004	21.602	195.83	-2.49
**TS5-8**	-1731.534962	-1730.957358	-1731.666337	-1731.934605	427.881	21.561	196.449	-2.88
**8**	-1731.560583	-1730.982919	-1731.683353	-1731.956145	429.959	22.026	199.858	-1.44
**6**	-1732.759981	-1732.174648	-1732.883648	-1733.16455	441.799	21.862	197.004	-1.626
**TS8-9**	-1731.523772	-1730.943028	-1731.648877	-1731.921074	428.996	21.664	196.747	-1.472
**9**	-1731.544441	-1730.958649	-1731.666998	-1731.940567	428.658	22.143	202.26	-1.894
**TS9-10**	-1731.52687	-1730.944821	-1731.652076	-1731.92418	427.12	22.075	202.415	-1.274
**10**	-1731.56741	-1730.982598	-1731.68956	-1731.960219	430.381	22.076	200.763	-2.081
**11**	-1732.768286	-1732.18266	-1732.891975	-1733.17253	442.495	22.15	199.32	-1.407
**TS11-12**	-1732.753398	-1732.16617	-1732.873798	-1733.155324	441.295	22.202	202.459	-1.721
**12**	-1732.800722	-1732.211781	-1732.916501	-1733.19715	442.902	22.971	207.492	-1.422
**7**	-1733.996059	-1733.399765	-1734.110979	-1734.401325	455.252	22.878	207.959	-1.477
**TS8-22**	-1731.461798	-1730.885207	-1731.589863	-1731.858216	427.563	22.153	202.274	-2.832
**TS9-22**	-1731.504849	-1730.919703	-1731.626778	-1731.899387	427.391	22.16	204.714	-1.083
**22**	-1731.56987	-1730.98283	-1731.688508	-1731.959252	429.747	22.815	208.526	-2.06
**23**	-1732.775011	-1732.179824	-1732.892662	-1733.174388	441.269	22.813	206.281	-1.721
**TS23-12**	-1732.765771	-1732.175854	-1732.887392	-1733.166547	440.307	22.561	204.709	-2.043
**TS8-13**	-1731.536566	-1730.961347	-1731.662704	-1731.932235	429.822	21.66	197.701	-2.613
**13**	-1731.539943	-1730.963086	-1731.664507	-1731.934064	432.065	21.95	200.302	-2.559
**TS13-10**	-1731.491511	-1730.91784	-1731.620176	-1731.885375	430.187	21.912	199.651	-1.908
**14**	-1732.747008	-1732.15687	-1732.867267	-1733.148915	443.138	22.048	198.991	-3.111
**TS14-11**	-1732.673424	-1732.08505	-1732.796933	-1733.075015	441.894	21.943	198.536	-2.942
**15**	-1730.363211	-1729.792836	-1730.495626	-1730.752842	417.617	21.616	197.585	-2.544
**Benzene**	-232.253147	-232.113806	-232.271501	-232.316161	63.142	3.345	69.018	-1.175
**2-*anti***	-1963.840796	-1963.130135	-1963.990466	-1964.314608	494.979	26.095	229.656	-2.054
**TS2-24 *anti***	-1963.817256	-1963.114376	-1963.970572	-1964.28932	494.702	25.79	227.948	-1.537
**2 *syn***	-1963.835815	-1963.125172	-1963.984383	-1964.309175	495.293	26.114	232.496	-2.085
**TS2-24 *syn***	-1963.813798	-1963.111213	-1963.965537	-1964.285431	494.667	25.898	230.488	-1.56
**24**	-1731.580919	-1730.996436	-1731.697381	-1731.966898	432.889	22.477	205.603	-0.892

Table S3. The calculated energies at B3LYP level with LAVCP** basis set and the single-point energies at M06 , M06-L or B3LYP level with LAVC3P**++ basis set, respectively at 270K.

Molecules /complexes	Energy B3LYP LACVP** [hartree]	Energy M06 LACV3P**++ [hartree]	Energy M06-L LACV3P**++ [hartree]	Energy B3LYP-D3 LACV3P**++ [hartree]	Zero-pt E [kcal/mol]	Enthalpy [kcal/mol]	Entropy [cal/mol K]	Solvation E [kcal/mol]
**Benzene**	-232.253147	-232.113806	-232.271501	-232.316161	63.142	2.836	67.225	-1.175
**2 *anti***	-1963.840796	-1963.130135	-1963.990466	-1964.314608	494.979	22.126	215.756	-2.054
**TS2-24 *anti***	-1963.817256	-1963.114376	-1963.970572	-1964.28932	494.702	21.416	212.552	-1.537
**2 *syn***	-1963.835815	-1963.125172	-1963.984383	-1964.309175	495.293	21.704	216.968	-2.085
**TS2-24 *syn***	-1963.813798	-1963.111213	-1963.965537	-1964.285431	494.667	21.521	215.081	-1.56
**24**	-1731.580919	-1730.996436	-1731.697381	-1731.966898	432.889	18.694	192.285	-0.892
**12**	-1732.800722	-1732.211781	-1732.916501	-1733.19715	442.902	19.078	193.784	-1.422

Table S4. The calculated energies at B3LYP level with LAVCP** basis set and the single-point energies at M06, M06-L or B3LYP level with LAVC3P**++ basis set, respectively at 393K.

Molecules /complexes	Energy B3LYP LACVP** [hartree]	Energy M06 LACV3P**++ [hartree]	Energy M06-L LACV3P**++ [hartree]	Energy B3LYP-D3 LACV3P**++ [hartree]	Zero-pt E [kcal/mol]	Enthalpy [kcal/mol]	Entropy [cal/mol K]	Solvation E [kcal/mol]
**H_2**	-1.178539	-1.17026	-1.171479	-1.171479	6.384	2.733	33.053	0.000
***tert*-butylethylene$^-$**	-235.860939	-235.712436	-235.873665	-235.93135	103.482	8.723	92.119	-0.699
***tert*-butylethane**	-237.09896	-236.945831	-237.110185	-237.168529	118.244	9.27	95.149	-0.228
**5**	-1731.570932	-1730.990513	-1731.701294	-1731.96998	429.004	36.639	239.375	-2.49
**TS5-8**	-1731.534962	-1730.957358	-1731.666337	-1731.934605	427.881	36.473	239.63	-2.88
**8**	-1731.560583	-1730.982919	-1731.683353	-1731.956145	429.959	37.109	243.54	-1.44
**6**	-1732.759981	-1732.174648	-1732.883648	-1733.16455	441.799	37.196	241.407	-1.626
**TS8-9**	-1731.523772	-1730.943028	-1731.648877	-1731.921074	428.996	36.63	240.087	-1.472
**9**	-1731.544441	-1730.958649	-1731.666998	-1731.940567	428.658	37.281	246.101	-1.894
**TS9-10**	-1731.52687	-1730.944821	-1731.652076	-1731.92418	427.12	37.12	245.985	-1.274
**10**	-1731.56741	-1730.982598	-1731.68956	-1731.960219	430.381	37.138	244.384	-2.081
**11**	-1732.768286	-1732.18266	-1732.891975	-1733.17253	442.495	37.544	243.9	-1.407
**TS11-12**	-1732.753398	-1732.16617	-1732.873798	-1733.155324	441.295	37.485	246.715	-1.721
**12**	-1732.800722	-1732.211781	-1732.916501	-1733.19715	442.902	38.477	252.404	-1.422
**7**	-1733.996059	-1733.399765	-1734.110979	-1734.401325	455.252	38.602	253.494	-1.477
**TS8-22**	-1731.461798	-1730.885207	-1731.589863	-1731.858216	427.563	37.232	245.945	-2.832
**TS9-22**	-1731.504849	-1730.919703	-1731.626778	-1731.899387	427.391	37.218	248.323	-1.083
**22**	-1731.56987	-1730.98283	-1731.688508	-1731.959252	429.747	38.025	252.582	-2.06
**23**	-1732.775011	-1732.179824	-1732.892662	-1733.174388	441.269	38.325	251.209	-1.721
**TS23-12**	-1732.765771	-1732.175854	-1732.887392	-1733.166547	440.307	37.937	249.24	-2.043
**TS8-13**	-1731.536566	-1730.961347	-1731.662704	-1731.932235	429.822	36.548	240.816	-2.613
**13**	-1731.539943	-1730.963086	-1731.664507	-1731.934064	432.065	36.899	243.597	-2.559
**TS13-10**	-1731.491511	-1730.91784	-1731.620176	-1731.885375	430.187	36.862	242.948	-1.908
**14**	-1732.747008	-1732.15687	-1732.867267	-1733.148915	443.138	37.348	243.297	-3.111
**TS14-11**	-1732.673424	-1732.08505	-1732.796933	-1733.075015	441.894	37.186	242.674	-2.942
**15**	-1730.363211	-1729.792836	-1730.495626	-1730.752842	417.617	36.361	240.289	-2.544

Table S5. The calculated energies at B3LYP level with LAVCP** basis set and the single-point energies at M06, M06-L or B3LYP level with LAVC3P**++ basis set, respectively at 413K.

Molecules /complexes	Energy B3LYP LACVP** [hartree]	Energy M06 LACV3P**++ [hartree]	Energy M06-L LACV3P**++ [hartree]	Energy B3LYP-D3 LACV3P**++ [hartree]	Zero-pt E [kcal/mol]	Enthalpy [kcal/mol]	Entropy [cal/mol K]	Solvation E [kcal/mol]
**H_2**	-1.178539	-1.17026	-1.171479	-1.171479	6.384	2.873	33.399	0.000
**5**	-1731.570932	-1730.990513	-1731.701294	-1731.96998	429.004	40.266	248.375	-2.49
**TS5-8**	-1731.534962	-1730.957358	-1731.666337	-1731.934605	427.881	40.07	248.556	-2.88
**8**	-1731.560583	-1730.982919	-1731.683353	-1731.956145	429.959	40.739	252.548	-1.44
**6**	-1732.759981	-1732.174648	-1732.883648	-1733.16455	441.799	40.899	250.594	-1.626
**TS8-9**	-1731.523772	-1730.943028	-1731.648877	-1731.921074	428.996	40.237	249.036	-1.472
**9**	-1731.544441	-1730.958649	-1731.666998	-1731.940567	428.658	40.926	255.145	-1.894
**TS9-10**	-1731.52687	-1730.944821	-1731.652076	-1731.92418	427.12	40.742	254.974	-1.274
**10**	-1731.56741	-1730.982598	-1731.68956	-1731.960219	430.381	40.764	253.382	-2.081
**11**	-1732.768286	-1732.18266	-1732.891975	-1733.17253	442.495	41.255	253.11	-1.407
**TS11-12**	-1732.753398	-1732.16617	-1732.873798	-1733.155324	441.295	41.17	255.859	-1.721
**12**	-1732.800722	-1732.211781	-1732.916501	-1733.19715	442.902	42.201	261.645	-1.422
**7**	-1733.996059	-1733.399765	-1734.110979	-1734.401325	455.252	42.392	262.899	-1.477
**TS8-22**	-1731.461798	-1730.885207	-1731.589863	-1731.858216	427.563	40.859	254.944	-2.832
**TS9-22**	-1731.504849	-1730.919703	-1731.626778	-1731.899387	427.391	40.841	257.315	-1.083
**22**	-1731.56987	-1730.98283	-1731.688508	-1731.959252	429.747	41.674	261.636	-2.06
**23**	-1732.775011	-1732.179824	-1732.892662	-1733.174388	441.269	42.056	260.466	-1.721
**TS23-12**	-1732.765771	-1732.175854	-1732.887392	-1733.166547	440.307	41.637	258.422	-2.043
**TS8-13**	-1731.536566	-1730.961347	-1731.662704	-1731.932235	429.822	40.137	249.72	-2.613
**13**	-1731.539943	-1730.963086	-1731.664507	-1731.934064	432.065	40.499	252.53	-2.559
**TS13-10**	-1731.491511	-1730.91784	-1731.620176	-1731.885375	430.187	40.46	251.876	-1.908
**14**	-1732.747008	-1732.15687	-1732.867267	-1733.148915	443.138	41.039	252.456	-3.111
**TS14-11**	-1732.673424	-1732.08505	-1732.796933	-1733.075015	441.894	40.865	251.803	-2.942
**15**	-1730.363211	-1729.792836	-1730.495626	-1730.752842	417.617	39.908	249.091	-2.544

Cartesian coordinates for all the species calculated at the B3LYP level in this study

H_2

H1	0.0000000000	0.0000000000	0.3713943308
H2	0.0000000000	0.0000000000	-0.3713943308

***tert*-butylethylene**

C1	0.7651332043	1.4629448585	2.0755962239
C5	2.1720337213	-0.6276716487	2.5465373976
C6	1.8737161275	0.6588328547	2.7312955801
H7	2.9958532568	-1.0938586052	3.0793806169
H8	2.4811506399	1.2336984855	3.4337273358
H9	1.6161863896	-1.2667077283	1.8670633433
C9	-0.1551669063	2.0166033977	3.1882470426
H10	0.4100880199	2.6210979001	3.9065221202
H11	-0.9399259677	2.6518327877	2.7613436302
H12	-0.6370699729	1.2031764366	3.7401934638
C12	1.4082337543	2.6487858239	1.3197992033
H13	2.0100445438	3.2690053963	1.9935980291
H14	2.0622926522	2.2946138276	0.5164009253
H15	0.6374388525	3.2890832478	0.8754297310
C15	-0.0679938108	0.6225522603	1.0944218345
H16	-0.8572566155	1.2347114306	0.6446452018
H17	0.5507657032	0.2267229071	0.2824470125
H18	-0.5480066504	-0.2226054603	1.5989399605

***tert*-butylethane**

C1	0.6637642391	1.4119101159	2.1641072484
H2	3.0839173806	-1.0875510878	2.9099845115
H3	3.1756859794	0.0362835823	1.5546185396
C4	-0.1358700484	2.2936141083	3.1440308030
H5	0.5316807531	2.9055117029	3.7618485735
H6	-0.8084165916	2.9722554059	2.6068570321
H7	-0.7472383913	1.6825669367	3.8182559471
C8	1.4952867036	2.3224530231	1.2392418046
H9	2.2202670671	2.9121923945	1.8125208274
H10	2.0486712223	1.7475969369	0.4896258721
H11	0.8477861987	3.0241783299	0.7009618534
C12	-0.3243733678	0.5845025383	1.3188777655
H13	-1.0202250157	1.2403278738	0.7830140190
H14	0.1904201093	-0.0270670927	0.5707880810
H15	-0.9190998175	-0.0867154509	1.9495838845
C16	1.5865946013	0.4837259243	2.9989015449
H17	2.2221209591	1.1169034980	3.6334019014
H18	0.9551937228	-0.0937441738	3.6883361312
C19	2.4814109738	-0.4879952583	2.2192197056
H20	1.8974267830	-1.1846812233	1.6091118995

5

H1	0.3797773456	-0.2145413436	2.2434129500
Ir2	1.7106691005	0.0138979453	1.3078018992
P3	1.4573878783	2.3378060972	1.2610602464
P4	1.7344798985	-2.1873210583	0.5825566212
C5	2.1230422727	3.7335390671	2.3417822603
C6	1.3460600461	3.6813056165	3.6744700595
H7	1.4051183101	2.6894057820	4.1339569555
H8	0.2899746886	3.9392575767	3.5464809851
H9	1.7801972720	4.4049271067	4.3752479984
C10	1.9392024386	5.1096857074	1.6755908948
H11	2.2942325397	5.8972699252	2.3526534153
H12	0.8891165387	5.3220282268	1.4489564098
H13	2.5115544005	5.1922186895	0.7466181062
C14	3.6157193668	3.4671335710	2.6229891902
H15	4.2232889145	3.5334211759	1.7164894515
H16	3.7657307331	2.4746454031	3.0575701878
H17	3.9908029633	4.2160564631	3.3318113803
C18	1.9451863546	2.3345493274	-0.5650997685
C19	1.2624999322	3.3303526881	-1.5243493322
H20	1.6254012195	4.3520626680	-1.3648575643

H21	0.1751716887	3.3447525595	-1.4427001369
H22	1.5083154993	3.0570532397	-2.5586467331
C23	3.4641911849	2.4460969710	-0.7858567960
H24	3.7030613622	2.1366400748	-1.8117270532
H25	4.0223567936	1.8049926117	-0.1023801898
H26	3.8086008493	3.4804805088	-0.6754259039
C27	1.4874501470	0.8577306194	-0.7308347429
H28	3.3391505819	0.1521649709	1.0966073910
H29	1.9765895822	0.3251186217	-1.5449056801
H30	2.3172604748	-0.3123052272	2.7695269846
C31	0.9710151591	-3.5883079350	1.6989607847
C32	-0.0428195838	-2.9662284881	2.6817238694
H33	-0.4812804595	-3.7733587901	3.2832931663
H34	-0.8590295911	-2.4435985824	2.1821791589
H35	0.4390253447	-2.2496840844	3.3483779406
C36	2.0490875455	-4.2583995792	2.5778227656
H37	2.6161869475	-3.5215313971	3.1536679526
H38	2.7487195129	-4.8739157011	2.0094118425
H39	1.5492244229	-4.9203576441	3.2961285948
C40	0.2470063188	-4.6682885786	0.8676510843
H41	0.8843042187	-5.1244264762	0.1060694295
H42	-0.6438977703	-4.2719556292	0.3723510714
H43	-0.0883399203	-5.4704358433	1.5373744263
C44	3.4264345566	-2.8278862633	-0.1257875188
C45	4.5085108459	-2.7979876705	0.9770321986
H46	5.4863865893	-2.9976589705	0.5206149489
H47	4.3566420587	-3.5473755439	1.7530140068
H48	4.5480029489	-1.8132528553	1.4503135833
C49	3.3306580567	-4.2322597123	-0.7548136030
H50	2.5745096039	-4.2772740145	-1.5464028388
H51	3.1062698596	-5.0130485040	-0.0250017509
H52	4.2940299812	-4.4881642402	-1.2146286176
C53	3.9072158786	-1.8502227732	-1.2232976384
H54	3.9259469597	-0.8225069474	-0.8567723612
H55	3.3017134238	-1.8938252857	-2.1335393313
H56	4.9275432934	-2.1306212267	-1.5120113540
C57	-1.7026853737	-1.3765249836	-0.4161461651
C58	-0.3444825695	-0.8900275752	-0.9592495118
C59	0.1946895097	0.3934477051	-0.2769021635
C60	-0.8859088746	1.4421897729	0.1011120611
C61	-2.1741908679	0.8234864009	0.6663485134
C62	-2.7315433829	-0.2555075771	-0.2626835658
H63	-1.5581763720	-1.8456885457	0.5613902331
H64	-2.0819145360	-2.1591516670	-1.0868281056
H65	-0.5221461632	-0.6230373199	-2.0162798727
H66	-1.1801904732	1.9321387303	-0.8411141800
H67	-1.9568421168	0.3868313903	1.6495982487
H68	-2.9105698094	1.6230864254	0.8227369882
H69	-2.9663519221	0.1810885817	-1.2449998917
H70	-3.6724962202	-0.6579642601	0.1325223399
C71	-0.3677415522	2.5528862233	1.0676503036
H72	-0.6498409804	3.5530301877	0.7187991101
H73	-0.8118746417	2.4079763294	2.0569825111
C74	0.6769139430	-2.0594333659	-0.9805651216
H75	1.3919643116	-1.8992487184	-1.7891198411
H76	0.1672837305	-2.9982876065	-1.2160057648

TS5-8

H1	1.1438547849	-0.3018279151	2.6892548517
Ir2	1.8073991081	0.0194490272	1.2595622754
P3	1.4302117312	2.3309830081	1.2963006994
P4	1.7270306479	-2.1963129027	0.5977811738
C5	2.0675019695	3.7347077799	2.3862830949
C6	1.3479742386	3.5936935817	3.7454544587
H7	1.4845876377	2.5932401675	4.1683998140
H8	0.2741945360	3.7907762956	3.6662100514
H9	1.7661001359	4.3200368015	4.4530871017
C10	1.7801652898	5.1199480043	1.7783907988
H11	2.1150504019	5.9030355598	2.4708244545
H12	0.7120023864	5.2785657512	1.5971360864
H13	2.3119707266	5.2692131187	0.8336119843

C14	3.5822948817	3.5450298207	2.6043081373
H15	4.1523986252	3.6929793848	1.6830899187
H16	3.8046603487	2.5428829671	2.9820976990
H17	3.9386008358	4.2801703895	3.3368821496
C18	1.8420892156	2.4359088539	-0.5559219310
C19	1.0653531817	3.4174208994	-1.4513814404
H20	1.3855153243	4.4488910262	-1.2702920865
H21	-0.0156501514	3.3750655478	-1.3236047331
H22	1.2806756813	3.1930805829	-2.5043916046
C23	3.3447347413	2.6609084459	-0.8179790988
H24	3.5783885107	2.3982497833	-1.8581399802
H25	3.9762609475	2.0535750769	-0.1665928363
H26	3.6142046924	3.7139099620	-0.6851388276
C27	1.4714418721	0.9328664561	-0.7883907209
H28	2.7248598282	0.3644992942	-0.1404283408
H29	1.7949652313	0.5541394384	-1.7661743568
H30	3.1552777190	-0.1167952616	2.1741380243
C31	0.8503323325	-3.5134660317	1.7265316142
C32	-0.1593427410	-2.8025748077	2.6530986192
H33	-0.7193642690	-3.5668705600	3.2076072411
H34	-0.8752843593	-2.1809847417	2.1157591075
H35	0.3507072073	-2.1538662155	3.3665766715
C36	1.8543568073	-4.2185569032	2.6637084091
H37	2.4491663264	-3.4985933253	3.2326914962
H38	2.5302682154	-4.8980087510	2.1408265656
H39	1.2892082042	-4.8207776955	3.3863364636
C40	0.0960389248	-4.5756107362	0.8979579041
H41	0.7354927812	-5.0918214803	0.1778809994
H42	-0.7488762812	-4.1460966076	0.3531737495
H43	-0.3111371661	-5.3351550647	1.5775142515
C44	3.3985409206	-2.9590769440	-0.0434597853
C45	4.4460618935	-2.9542991309	1.0923702732
H46	5.4251568262	-3.2213607821	0.6745761602
H47	4.2278522620	-3.6699533413	1.8843211426
H48	4.5217588974	-1.9594509850	1.5396275314
C49	3.2404745786	-4.3750263620	-0.6328682467
H50	2.5114256254	-4.4011974573	-1.4502089980
H51	2.9431060070	-5.1157030250	0.1124220616
H52	4.2023202878	-4.7041525683	-1.0473834217
C53	3.9759235663	-2.0505475472	-1.1535295108
H54	4.0912775340	-1.0217388911	-0.8063322270
H55	3.3790591866	-2.0509313854	-2.0701221332
H56	4.9695061627	-2.4277005449	-1.4245853511
C57	-1.6817888005	-1.4010910487	-0.5481180070
C58	-0.3029414327	-0.8787479939	-1.0046903543
C59	0.1987252168	0.3766013315	-0.2420474881
C60	-0.9020505858	1.3936591098	0.1506876091
C61	-2.1723737449	0.7135151178	0.6845385367
C62	-2.7174403540	-0.2987051944	-0.3237775262
H63	-1.5710884451	-1.9568709800	0.3873828406
H64	-2.0410276718	-2.1233133890	-1.2936038644
H65	-0.4287167340	-0.5928773404	-2.0667119470
H66	-1.2253383834	1.9313925163	-0.7572158761
H67	-1.9368719693	0.2070515282	1.6302064524
H68	-2.9221858387	1.4842186332	0.9083920554
H69	-2.9449144917	0.2100103925	-1.2724180457
H70	-3.6601566971	-0.7340937169	0.0305355152
C71	-0.4098659408	2.4609730917	1.1774431175
H72	-0.7570540360	3.4673239088	0.9133694601
H73	-0.8079528806	2.2218051250	2.1686539564
C74	0.7271070458	-2.0443981930	-1.0045662099
H75	1.4815092874	-1.8535826268	-1.7707883832
H76	0.2429621351	-2.9834681301	-1.2911709327

8

C1	2.5438256468	-0.8375143114	0.2968702954
Ir2	2.8547682475	-0.8848234107	2.5018166995
P3	3.1013829263	1.3037063946	1.8020757546
P4	2.8683425489	-3.1936506651	2.3049085897
C5	3.3274398883	2.8453210745	2.8824990015
C6	1.9909718590	3.0343123721	3.6340967017

H7	1.6907042436	2.1173615389	4.1512825900
H8	1.1800079890	3.3337691142	2.9626173915
H9	2.1070681980	3.8252608408	4.3852586302
C10	3.6564591137	4.1244329186	2.0918480600
H11	3.6444107938	4.9871513785	2.7706357296
H12	2.9281438954	4.3221771501	1.2984195370
H13	4.6516032668	4.0815817141	1.6401808046
C14	4.4350924377	2.5644228469	3.9185534265
H15	5.4178715410	2.4493308610	3.4527445115
H16	4.2212278615	1.6550502998	4.4871934540
H17	4.4978559569	3.4048163751	4.6214629246
C18	4.4132945380	1.0470915705	0.4452666549
C19	4.4355791094	2.1200476878	-0.6617593177
H20	4.7399088144	3.1004302817	-0.2858153567
H21	3.4698362948	2.2323844971	-1.1616321329
H22	5.1617133341	1.8265888097	-1.4306634151
C23	5.8253784744	0.9333789734	1.0480761570
H24	6.5247869929	0.5724858940	0.2831369676
H25	5.8470785279	0.2312784495	1.8865018824
H26	6.2007341103	1.9014567693	1.3984835198
C27	1.3599010394	-4.2171157140	2.9776270408
C28	0.1676408344	-3.2435829409	3.1181241064
H29	-0.6980038903	-3.7916197011	3.5134088946
H30	-0.1315247919	-2.7933842596	2.1710343719
H31	0.4094731176	-2.4275370424	3.8039254446
C32	1.6249730139	-4.7722790423	4.3923206593
H33	1.9353861048	-3.9879467283	5.0881233462
H34	2.3709013785	-5.5700267836	4.4050912361
H35	0.6904956182	-5.1998455183	4.7776007547
C36	0.9711848397	-5.3867090645	2.0521467191
H37	1.7931098506	-6.0939652472	1.9047710485
H38	0.6386806598	-5.0443941493	1.0691515230
H39	0.1378695174	-5.9424405720	2.5017113407
C40	4.5209694657	-4.0383400152	2.8511088631
C41	4.7488519138	-3.8862319120	4.3721309178
H42	5.7783914465	-4.1844546676	4.6078129230
H43	4.0893168511	-4.5113664469	4.9725307304
H44	4.6189103599	-2.8468807318	4.6857902269
C45	4.5993447839	-5.5221255431	2.4424041276
H46	4.4674200845	-5.6587791632	1.3638477697
H47	3.8561994994	-6.1387589598	2.9550632254
H48	5.5877815413	-5.9233898350	2.7025400464
C49	5.6793442737	-3.2725340029	2.1682827656
H50	5.6145578597	-2.2004484293	2.3735526225
H51	5.7145415873	-3.4166679079	1.0850140974
H52	6.6309618638	-3.6429210310	2.5690203148
C53	0.7652668775	-2.5686015799	-0.5710349166
C54	2.2570243072	-2.2367561210	-0.3472670272
C55	1.5371532176	0.2678280243	-0.1795585704
C56	0.0760885832	-0.1908981209	-0.2834378026
C57	-0.0447578746	-1.4236561423	-1.1789354525
H58	0.3006009101	-2.8373958344	0.3821432664
H59	0.7016761730	-3.4643638810	-1.2038871548
H60	2.7111193895	-2.2369042073	-1.3564086644
H61	-0.3060151103	-0.4266472456	0.7202202828
H62	-0.5354553667	0.6362307195	-0.6695120934
H63	0.3258850318	-1.1865256324	-2.1869815977
H64	-1.0943053317	-1.7229091045	-1.2935673849
C65	1.6274619737	1.5465303268	0.7132381226
H66	1.6603147140	2.4691090526	0.1219905761
H67	0.7484951503	1.6063023286	1.3628940114
C68	2.9354303028	-3.4034127977	0.4207142518
H69	4.0020292079	-3.4124685628	0.1867040120
H70	2.5469816081	-4.3715538489	0.0867678740
H71	2.3458926510	-0.7779777133	4.0084085646
C72	3.9611763911	-0.3499288934	-0.1491948344
H73	3.9914871115	-0.2778528276	-1.2524817202
H74	4.7157072067	-1.0940059019	0.1265597800
H75	3.9261831038	-0.8538104466	3.6771439900
H76	1.8311095458	0.5606747845	-1.2049459077

6

C1	2.6022792844	-0.7986988002	0.2677718995
Ir2	2.8741737926	-0.8699655651	2.5115247079
P3	3.1154691224	1.3162952078	1.7734987011
P4	2.8670605682	-3.2020371527	2.3213612395
C5	3.3442136378	2.8243000065	2.8961032779
C6	2.0361632179	2.9559232036	3.7072212266
H7	1.7793867953	2.0170682835	4.2067046639
H8	1.1917077554	3.2543745026	3.0777649019
H9	2.1650809648	3.7279781679	4.4756065849
C10	3.6063020764	4.1346421524	2.1311105178
H11	3.6019861186	4.9747254132	2.8376128508
H12	2.8389920064	4.3375241488	1.3768482781
H13	4.5817721267	4.1322287881	1.6366998148
C14	4.5051693208	2.5435834110	3.8721820208
H15	5.4666879370	2.4603810109	3.3576299383
H16	4.3378119861	1.6175769660	4.4289845580
H17	4.5830987762	3.3694139490	4.5905265440
C18	4.3735142005	1.1548121282	0.3465713374
C19	4.2742170079	2.2651782476	-0.7230522286
H20	4.5284967275	3.2509208454	-0.3253288887
H21	3.2847625392	2.3290169757	-1.1820353758
H22	4.9864963687	2.0476501166	-1.5286056335
C23	5.8278904614	1.1152069731	0.8494912684
H24	6.4881888214	0.8042449329	0.0300380056
H25	5.9561527318	0.4066811566	1.6711827334
H26	6.1701358357	2.1017061472	1.1827679711
C27	1.3292302381	-4.2225619279	2.9456110670
C28	0.0942993575	-3.2987351521	3.0215957860
H29	-0.7660474973	-3.8970229480	3.3483500782
H30	-0.1654476656	-2.8394594657	2.0684319217
H31	0.2496671189	-2.4907921798	3.7383204421
C32	1.5365417930	-4.7339346899	4.3889453556
H33	1.7901978080	-3.9182472811	5.0723183052
H34	2.2973153191	-5.5108940835	4.4743199437
H35	0.5925758890	-5.1696865190	4.7395307716
C36	1.0050005289	-5.4142567152	2.0203536322
H37	1.8413538687	-6.1086905581	1.9103806800
H38	0.7044365961	-5.0864423570	1.0217887384
H39	0.1639038718	-5.9791064067	2.4426434035
C40	4.4746795669	-4.1325036735	2.9066316191
C41	4.7215252531	-3.8651695303	4.4081100545
H42	5.6808977600	-4.3130326282	4.6967319841
H43	3.9575038972	-4.2912000589	5.0575189066
H44	4.7748360178	-2.7914374273	4.6051366413
C45	4.4291582157	-5.6494374029	2.6237712068
H46	4.2633200803	-5.8611693783	1.5620085163
H47	3.6658085576	-6.1759464872	3.1986958926
H48	5.3967990573	-6.0921235504	2.8927133881
C49	5.7111932008	-3.5790340864	2.1592819032
H50	5.7742490329	-2.4914501684	2.2188317417
H51	5.7414511153	-3.8757753126	1.1070951511
H52	6.6094167203	-3.9965559070	2.6299726739
C53	0.8464421920	-2.5746589184	-0.6032883382
C54	2.3278399496	-2.2146511598	-0.3479732836
C55	1.5272331350	0.2728204584	-0.1608866314
C56	0.0836236247	-0.2287805081	-0.2788292853
C57	0.0121798484	-1.4436540112	-1.2035514675
H58	0.3743290847	-2.8743101936	0.3355869796
H59	0.8197317028	-3.4604836876	-1.2525409635
H60	2.8050056897	-2.2226318120	-1.3447235498
H61	-0.2997596235	-0.4961211089	0.7149216117
H62	-0.5477462951	0.5890430503	-0.6524669542
H63	0.3893343096	-1.1755436767	-2.2015276758
H64	-1.0254642930	-1.7737389384	-1.3394464635
C65	1.5926492494	1.5354014683	0.7541340166
H66	1.5650973476	2.4689128438	0.1811791379
H67	0.7437886019	1.5393559895	1.4432936808
C68	3.0038278820	-3.3645622955	0.4420699597
H69	4.0797201278	-3.3214354047	0.2647296537
H70	2.6689034411	-4.3408740332	0.0739835316

H71	2.1006165992	-0.6922446706	3.9245984707
C72	3.9534159179	-0.2392356865	-0.2735620991
H73	3.8904599888	-0.1157610291	-1.3692104042
H74	4.7514731769	-0.9623878472	-0.0850252429
H75	1.2460054908	-0.7709296693	2.3673486165
H76	3.8227706765	-0.8141755654	3.8268075550
H77	1.8023728758	0.5953277063	-1.1801796545
H78	4.4414428181	-1.0515331286	2.0875277912

TS8-9

H1	1.7463988238	-0.1050314735	2.8130756150
Ir2	1.6024981790	0.1166842012	1.2504859789
P3	0.9614723744	2.3426021284	1.1577507988
P4	1.9545087505	-2.1293148179	0.8022858497
C5	1.2528687521	3.7605677751	2.3765550568
C6	0.3397197162	3.5059022813	3.5958128425
H7	0.4802548292	2.4975478204	3.9987853383
H8	-0.7196949936	3.6372764193	3.3550730794
H9	0.5879485114	4.2229262477	4.3879813075
C10	0.9295301715	5.1344812456	1.7604729044
H11	1.0464784915	5.9172552513	2.5211101993
H12	-0.0998020554	5.1892010761	1.3901859702
H13	1.6023395649	5.3792594451	0.9329986307
C14	2.7203158110	3.7143304586	2.8498227346
H15	3.4227734372	3.9340481807	2.0420588399
H16	2.9725719823	2.7302898301	3.2545140532
H17	2.8714238056	4.4650818202	3.6359649387
C18	1.6620554926	2.5551637624	-0.5886969866
C19	3.1546823515	2.9311526529	-0.5604704852
H20	3.7166490072	2.2768029079	0.1106530289
H21	3.3062454192	3.9708676082	-0.2499006482
H22	3.5721620323	2.8288848200	-1.5699222398
C23	1.5068813992	1.0546098223	-1.0131609286
H24	0.8078607418	0.9290512783	-1.8401892538
H25	3.1858987166	0.4534474425	1.5886183736
H26	2.4578953998	0.6148616961	-1.3080944717
C27	0.9214475857	3.5200520984	-1.5304744969
H28	1.3445806655	3.4364259248	-2.5401841292
H29	1.0304212908	4.5636114033	-1.2155287874
H30	-0.1465593661	3.3008602547	-1.6069519226
C31	1.4567679639	-3.3590149881	2.2002620840
C32	2.2243076887	-3.0111923563	3.4942208711
H33	3.2996833984	-3.1763449448	3.4091784824
H34	1.8560613686	-3.6519248168	4.3056711385
H35	2.0632794434	-1.9690227272	3.7782116550
C36	1.6739133808	-4.8454365554	1.8588253690
H37	2.7322126148	-5.1157507244	1.8245366467
H38	1.2133891542	-5.1296315302	0.9065090747
H39	1.2102730300	-5.4626656691	2.6394226434
C40	-0.0448928946	-3.1450457685	2.4823283358
H41	-0.6693032069	-3.5434231839	1.6780157680
H42	-0.2799676877	-2.0864354530	2.6200873017
H43	-0.3148668016	-3.6843362539	3.3989311241
C44	3.6566712973	-2.6612144286	0.0611789683
C45	4.7163354992	-2.7625588882	1.1761581320
H46	5.7105464537	-2.8562346629	0.7210160492
H47	4.5700421410	-3.6407551054	1.8106665976
H48	4.7189130416	-1.8688476180	1.8072844263
C49	3.6207045897	-3.9809152936	-0.7388258167
H50	2.9036683403	-3.9496304591	-1.5648340116
H51	3.3864616378	-4.8493461338	-0.1226911832
H52	4.6104982553	-4.1529756990	-1.1809679406
C53	4.0996750756	-1.5358269793	-0.8979459856
H54	4.1814841921	-0.5823892125	-0.3716188852
H55	3.4102345971	-1.4097571402	-1.7395821504
H56	5.0809658748	-1.7907911474	-1.3183512762
C57	-1.7245019221	-1.8558672193	-0.4913422993
C58	-0.3039297560	-1.3002171957	-0.7825093929
C59	-0.0242773304	-0.0464864225	0.0655369618
C60	-1.1319676449	1.0151278247	-0.0622988582
C61	-2.5114646094	0.4003384402	0.2663269368

C62	-2.8161549058	-0.7932239592	-0.6413688640
H63	-1.7582801103	-2.2497647757	0.5291956821
H64	-1.9148111894	-2.7013722338	-1.1652032297
H65	-0.3246530303	-1.0038556469	-1.8467859449
H66	-1.2019089018	1.3556614811	-1.1099942165
H67	-2.5168196087	0.0768988898	1.3161220688
H68	-3.2819399814	1.1763354764	0.1668645541
H69	-2.8725014795	-0.4571899648	-1.6869575836
H70	-3.7962022627	-1.2223957389	-0.3981906466
C71	-0.8612110204	2.2468096981	0.8359856865
H72	-1.2853634577	3.1636499257	0.4098716094
H73	-1.3327861502	2.0862796190	1.8109002321
C74	0.7762508266	-2.4128978532	-0.6429049816
H75	1.4041167897	-2.4177819278	-1.5385747326
H76	0.3155922198	-3.4061533005	-0.5951209794

9

H1	1.3181900264	-0.0675611354	2.7807596138
Ir2	1.5241512059	0.1276133594	1.1819966572
P3	1.0040160238	2.3536771569	1.1004420406
P4	1.8697649039	-2.1833397953	0.9160281378
C5	0.8328651303	3.5785688284	2.5419259438
C6	-0.3299641924	3.0950624960	3.4358468970
H7	-0.2004332163	2.0471682793	3.7241081499
H8	-1.3065790828	3.2016308252	2.9546277377
H9	-0.3527671876	3.6998230396	4.3504201158
C10	0.5506459318	5.0022630827	2.0255865776
H11	0.4344786312	5.6900207826	2.8733384022
H12	-0.3720150699	5.0505055769	1.4369150395
H13	1.3692629787	5.3810360715	1.4047466588
C14	2.1182140641	3.5556866482	3.3933926057
H15	2.9744840459	3.9825244095	2.8686969794
H16	2.3744031947	2.5348499924	3.6909370316
H17	1.9568642934	4.1504281982	4.3010863967
C18	2.3353226168	2.6902271969	-0.1987949939
C19	3.6402077203	3.2414110642	0.3954552430
H20	3.9824216766	2.6364789693	1.2383767480
H21	3.5472530287	4.2853589160	0.7156552317
H22	4.4173999630	3.2049322852	-0.3786438109
C23	2.5054666952	1.1723081077	-0.5453099778
H24	1.9940516631	0.9456189598	-1.4859066958
H25	3.0396065066	0.3824004292	1.8572466844
H26	3.5591272866	0.9094448415	-0.6544822042
C27	1.9171298736	3.5554527457	-1.3965348469
H28	2.7169608285	3.5465570617	-2.1487267541
H29	1.7433654320	4.6007947782	-1.1106775582
H30	1.0115228186	3.1814629009	-1.8843940128
C31	1.9650715086	-3.2960944027	2.4894794003
C32	3.3625096340	-3.1733759337	3.1271375132
H33	4.1379812028	-3.6560978343	2.5255035242
H34	3.3599521725	-3.6657266505	4.1078263489
H35	3.6401220783	-2.1254043264	3.2759538613
C36	1.6365482382	-4.7811373378	2.2175279155
H37	2.3605641171	-5.2740469751	1.5701317196
H38	0.6432606177	-4.9101099200	1.7761172608
H39	1.6343805373	-5.3212682603	3.1726733974
C40	0.9276979755	-2.7811378121	3.5115123001
H41	-0.0938151712	-2.7967620716	3.1176552125
H42	1.1429585267	-1.7596864358	3.8259252375
H43	0.9521124336	-3.4358499959	4.3919309190
C44	3.2321263666	-2.7136070882	-0.3392582744
C45	4.5571586007	-2.0203261640	0.0425752073
H46	5.2936212632	-2.1806190542	-0.7556375520
H47	4.9823631612	-2.4212759878	0.9661293814
H48	4.4168806814	-0.9466364451	0.1804494141
C49	3.4726431176	-4.2289579838	-0.4749819341
H50	2.5529290815	-4.7841064367	-0.6868026848
H51	3.9409989400	-4.6597714248	0.4131689323
H52	4.1574523014	-4.4045489581	-1.3149546736
C53	2.7850307507	-2.1868040000	-1.7212796647
H54	2.5536956002	-1.1216173557	-1.6962071123

H55	1.9114152017	-2.7253930333	-2.1023322851
H56	3.5990378855	-2.3391222825	-2.4408727731
C57	-1.9947179355	-1.7507161988	-0.5779908287
C58	-0.4667824381	-1.5033991525	-0.5165216128
C59	-0.1003260807	-0.1608579965	0.1246334982
C60	-0.9066717130	1.0039398932	-0.4710111212
C61	-2.4277890622	0.7231295568	-0.5507934119
C62	-2.7357231144	-0.5979035336	-1.2606566095
H63	-2.3725208392	-1.8696114225	0.4476750466
H64	-2.1904718048	-2.6984458105	-1.0955989675
H65	-0.1217060064	-1.4308271153	-1.5636457401
H66	-0.5489095391	1.0911514738	-1.5140845331
H67	-2.8288064933	0.6867861730	0.4723179407
H68	-2.9290373022	1.5603338378	-1.0533652194
H69	-2.4290442794	-0.5331670689	-2.3146083054
H70	-3.8165575051	-0.7860592409	-1.2615298914
C71	-0.6494827760	2.3560552784	0.2365588871
H72	-0.7710200865	3.2019141135	-0.4486403415
H73	-1.3971358042	2.4807355316	1.0266354152
C74	0.2459171887	-2.7009778968	0.1446441321
H75	0.3559438079	-3.5391809383	-0.5525989330
H76	-0.3776682607	-3.0621312611	0.9695076528

TS9-10

H1	-0.0972134065	-0.2889020446	1.6631569542
Ir2	1.5039326340	0.1435499908	1.1462837818
P3	1.0330393017	2.3526531513	1.0170400423
P4	1.8904007951	-2.1560217956	0.8189461485
C5	0.7412177395	3.5012568064	2.5021915887
C6	-0.4868962797	2.9535516409	3.2628848240
H7	-0.3368259574	1.9089640158	3.5509220513
H8	-1.4117190043	3.0193894942	2.6813172164
H9	-0.6349932632	3.5446494937	4.1751526818
C10	0.4838130625	4.9526263324	2.0545059278
H11	0.2758645513	5.5804430600	2.9304540564
H12	-0.3806305797	5.0304232986	1.3859875483
H13	1.3496603428	5.3802727160	1.5405998877
C14	1.9496638336	3.4317012298	3.4565520587
H15	2.8371983973	3.9140059185	3.0417450396
H16	2.2004422348	2.3942101494	3.6953017273
H17	1.7016473848	3.9529760235	4.3895907631
C18	2.6476010222	2.6031946867	0.0237320424
C19	3.5253025334	3.8335678578	0.2900845636
H20	3.8345869049	3.9113211242	1.3341739756
H21	3.0332351731	4.7708401855	0.0023462246
H22	4.4411103993	3.7478374049	-0.3095827471
C23	3.2752621661	1.2472782102	0.5048603721
H24	3.8079150414	0.7719312271	-0.3268442536
H25	2.2365430555	0.1942125192	2.6283044168
H26	3.9920884683	1.4260482931	1.3103597347
C27	2.3252270288	2.5505966219	-1.4795504753
H28	3.2657267743	2.4900050308	-2.0412391388
H29	1.7950022615	3.4469634367	-1.8231552759
H30	1.7283554153	1.6698646297	-1.7320787018
C31	2.0280427403	-3.2705231557	2.3834271244
C32	3.4396737555	-3.1508772947	2.9906465084
H33	4.1935408996	-3.6723984384	2.3941964305
H34	3.4446093743	-3.6078192699	3.9883071132
H35	3.7395507609	-2.1041015951	3.0987335502
C36	1.6910539376	-4.7570283962	2.1436663583
H37	2.4096893969	-5.2588782371	1.4943739796
H38	0.6938687776	-4.8956151509	1.7146650547
H39	1.7009349234	-5.2825031875	3.1072395577
C40	1.0227753502	-2.7101083881	3.4154391149
H41	-0.0135284045	-2.7752426399	3.0678922689
H42	1.2315833402	-1.6634178458	3.6431711124
H43	1.0976491810	-3.3011844773	4.3374256129
C44	3.1867036427	-2.6641067754	-0.5050276339
C45	4.5821434197	-2.1477208173	-0.0948935633
H46	5.2734127180	-2.2650377616	-0.9394690662
H47	4.9994443429	-2.7062862233	0.7458475977

H48	4.5567576622	-1.0925197896	0.1808265684
C49	3.2728293610	-4.1753155782	-0.7897696898
H50	2.2964380808	-4.6243797032	-0.9995166852
H51	3.7323592089	-4.7241220586	0.0361436436
H52	3.9024797349	-4.3396367247	-1.6741401067
C53	2.7626147318	-1.9430086575	-1.8052731848
H54	2.6378973519	-0.8696334987	-1.6461081778
H55	1.8239094947	-2.3391356208	-2.2067262147
H56	3.5353650634	-2.0953400321	-2.5694887565
C57	-2.0888260391	-1.7142643464	-0.2025822090
C58	-0.5786675412	-1.4991366091	-0.4772432898
C59	-0.0726239720	-0.1282165255	-0.0125699528
C60	-0.9903209749	1.0246801978	-0.4604828738
C61	-2.4875190495	0.7845180063	-0.1573539276
C62	-2.9582160366	-0.5623322387	-0.7202596175
H63	-2.2255142869	-1.8074326923	0.8846168775
H64	-2.4146748916	-2.6659520433	-0.6427797391
H65	-0.4473547371	-1.4808242649	-1.5723440658
H66	-0.8915756730	0.9823941402	-1.5588601950
H67	-2.6359457760	0.8060929049	0.9317682525
H68	-3.0900889137	1.6014146901	-0.5758434557
H69	-2.9132511591	-0.5314817669	-1.8188485453
H70	-4.0092218704	-0.7375611860	-0.4590241323
C71	-0.5277228547	2.4474202454	-0.0216316937
H72	-0.3757317697	3.0946867486	-0.8904180403
H73	-1.3056625920	2.9259643092	0.5810099213
C74	0.2392004788	-2.6845599612	0.0909229331
H75	0.3524657354	-3.4797455186	-0.6535313033
H76	-0.3218980080	-3.1243736666	0.9214769198

10

H1	-1.0270142880	-0.4989682729	1.2443059048
Ir2	1.6131629961	0.1003509475	1.2345206329
P3	0.9071648819	2.2814998674	1.2755437400
P4	1.9113087788	-2.1640060882	0.7383999905
C5	0.8950052761	3.5914136587	2.6580644016
C6	-0.2192564528	3.2030508150	3.6559418621
H7	-0.0955832152	2.1768942268	4.0183231130
H8	-1.2205570347	3.2973889104	3.2264908494
H9	-0.1726695733	3.8710915286	4.5249604508
C10	0.6183876554	4.9996969667	2.0976492903
H11	0.5781533930	5.7266230634	2.9195056690
H12	-0.3396236889	5.0496933007	1.5694251833
H13	1.4030634907	5.3267871260	1.4086566606
C14	2.2378849051	3.5642212723	3.4161646687
H15	3.0643860020	3.9443280583	2.8138009618
H16	2.4942110345	2.5490412946	3.7326726371
H17	2.1605193438	4.1978211063	4.3088534284
C18	2.1010132979	2.6130050229	-0.1774108923
C19	3.4362583169	3.2266604404	0.2755451772
H20	3.8787839144	2.6503813845	1.0912332274
H21	3.3345622531	4.2722269065	0.5849354818
H22	4.1359511157	3.2062162517	-0.5698476768
C23	2.3000054161	1.0957031015	-0.4786976143
H24	1.7049589091	0.7872733546	-1.3451618478
H25	3.1041849272	0.3388390127	1.9572868078
H26	3.3529530452	0.8611277046	-0.6607307300
C27	1.5419718568	3.4245354263	-1.3558034163
H28	2.2617388542	3.4133026149	-2.1845281109
H29	1.3771072195	4.4727145117	-1.0800973157
H30	0.5996157988	3.0215297278	-1.7352761162
C31	1.9897757528	-3.0793289423	2.4206580084
C32	3.4141091366	-3.0206380716	3.0053627235
H33	4.1046470122	-3.6814673817	2.4731051664
H34	3.3897539384	-3.3544739186	4.0507099984
H35	3.8123394041	-2.0024268985	2.9818623378
C36	1.4945661729	-4.5378578954	2.4097032105
H37	2.1354367126	-5.1916115021	1.8137632790
H38	0.4725948149	-4.6310556915	2.0309364545
H39	1.4957740895	-4.9269247289	3.4363961574
C40	1.0625444608	-2.2347012049	3.3289750229

H41	0.0219866087	-2.2434692316	2.9913381066
H42	1.4088218677	-1.1878363501	3.3810448469
H43	1.0842213273	-2.6273637827	4.3541846327
C44	3.2357607484	-2.8034385674	-0.4823945915
C45	4.6005426883	-2.1776577260	-0.1265050815
H46	5.3254200047	-2.4157296321	-0.9157423896
H47	5.0008900925	-2.5614505449	0.8142320422
H48	4.5278118687	-1.0912185199	-0.0352998175
C49	3.3540870729	-4.3384737991	-0.5163910868
H50	2.3947513526	-4.8253736730	-0.7215164630
H51	3.7505276375	-4.7425071684	0.4188633445
H52	4.0466768428	-4.6323961766	-1.3160160541
C53	2.8311643797	-2.3089301402	-1.8894107471
H54	2.7242880189	-1.2222954186	-1.9246366396
H55	1.8958000654	-2.7576889426	-2.2366101413
H56	3.6145581405	-2.5910753828	-2.6041102341
C57	-1.8910813040	-1.7433599032	-1.0271715635
C58	-0.4387956693	-1.4474997442	-0.5927306844
C59	-0.3762113112	-0.2484449514	0.3798125707
C60	-0.9959717152	0.9947046336	-0.2974324349
C61	-2.4610653244	0.7375689045	-0.7097651706
C62	-2.5623147159	-0.4943197341	-1.6292687120
H63	-2.4615292680	-2.0768817379	-0.1469223861
H64	-1.9283230318	-2.5673581589	-1.7542386652
H65	0.1197565863	-1.1662515978	-1.4998993634
H66	-0.4394941687	1.1701950020	-1.2309245423
H67	-3.0634760192	0.5735467266	0.1964065745
H68	-2.8904941174	1.6124133059	-1.2186274474
H69	-2.0784271295	-0.2593197719	-2.5887980159
H70	-3.6136131994	-0.7070440907	-1.8616259855
C71	-0.8336648504	2.2521489963	0.5790581264
H72	-1.0911323491	3.1735677098	0.0428913245
H73	-1.5119341208	2.1782879122	1.4377227634
C74	0.2529962043	-2.6811175284	0.0164174177
H75	0.3449201699	-3.5095378382	-0.6958927849
H76	-0.3577427524	-3.0535047071	0.8477606707

11

H1	3.0237368521	0.0578255658	2.0686242680
Ir2	1.4455372304	-0.1300110495	1.8203238467
P3	1.3066473581	-2.4206626838	1.5113566557
P4	0.9388330570	2.1793957894	1.9072028581
C5	2.7156065273	-3.6751711857	1.2908354096
C6	3.3668101195	-3.4166625186	-0.0851648855
H7	3.6522770284	-2.3660759032	-0.2030298893
H8	2.7100967328	-3.6908298787	-0.9157923563
H9	4.2758400491	-4.0242058670	-0.1735637025
C10	2.1849639618	-5.1202930600	1.3575673934
H11	3.0091016248	-5.8266418699	1.1935310428
H12	1.4288494801	-5.3162937562	0.5900599432
H13	1.7452995022	-5.3492582314	2.3333320457
C14	3.7865624213	-3.4466033838	2.3760897222
H15	3.4215796428	-3.6780097428	3.3785283644
H16	4.1363977189	-2.4101873046	2.3733110409
H17	4.6459829926	-4.0992406891	2.1777165429
C18	0.2040501738	-2.6056383282	3.0343971051
C19	-0.9902627907	-3.5644454242	2.9202695705
H20	-0.6730027706	-4.6138152953	2.8769754921
H21	-1.6104889141	-3.3630097843	2.0429684156
H22	-1.6301432663	-3.4503769584	3.8049220701
C23	1.0014391109	-2.9607347804	4.3001725615
H24	0.3431142435	-2.8412356482	5.1701242001
H25	1.8579816364	-2.2964094237	4.4402696302
H26	1.3532163597	-3.9984221646	4.2985337453
C27	-0.1778684360	-1.0825244569	3.0634209490
H28	-0.2117964255	-0.7320862283	4.0984275122
H29	-1.1680645013	-0.9268943941	2.6302892148
H30	2.0221235425	-0.2117416403	3.3387095606
C31	2.3312694382	3.4136652731	1.3834163917
C32	3.1069771091	2.7979263845	0.1976860860
H33	3.8451996220	3.5301924220	-0.1533255722

H34	2.4564624285	2.5550607346	-0.6485617568
H35	3.6315586032	1.8869476212	0.4853127789
C36	3.3269269002	3.6121024799	2.5434276130
H37	3.6858908725	2.6532375484	2.9303408339
H38	2.8975993726	4.1799834072	3.3732212526
H39	4.1978195320	4.1743679032	2.1832014312
C40	1.7943347327	4.7831487229	0.9094960861
H41	1.2859644997	5.3447830228	1.6917011011
H42	1.1090259983	4.6850069827	0.0623130301
H43	2.6399700061	5.3937957966	0.5686583654
C44	0.0821524723	2.8394007933	3.5073940748
C45	0.8865605253	2.3727056177	4.7390405361
H46	0.3497455007	2.6639842662	5.6510909637
H47	1.8813307141	2.8232666016	4.7852196939
H48	1.0095971972	1.2876701584	4.7425028542
C49	-0.1079481706	4.3674540276	3.5744586122
H50	-0.6435702640	4.7607570734	2.7044934348
H51	0.8353620740	4.9088107645	3.6758627485
H52	-0.7114069846	4.6069438472	4.4597001790
C53	-1.3234611631	2.2031128882	3.5808491050
H54	-1.2877513387	1.1161319862	3.5200751802
H55	-1.9857627012	2.5753684707	2.7932768113
H56	-1.7800019623	2.4714346381	4.5420027392
C57	-1.9468164718	1.1130031779	-0.9819657213
C58	-1.0588601298	1.0468748826	0.2828359901
C59	-0.0509681904	-0.1099313496	0.1674473369
C60	-0.7876238598	-1.4488626259	-0.0339897620
C61	-1.6283480686	-1.4076939636	-1.3271728178
C62	-2.6283205079	-0.2367580591	-1.2767153780
H63	-1.3170055161	1.3963300775	-1.8387288219
H64	-2.7098050138	1.8978613824	-0.8828428628
H65	-1.7176094657	0.8438256541	1.1390513430
H66	0.5241936059	0.0638783075	-0.7516759370
H67	-1.5044526707	-1.5771977354	0.7870856657
H68	-0.9596715131	-1.2901899588	-2.1929055780
H69	-2.1714179222	-2.3518464500	-1.4737164440
H70	-3.3706644789	-0.4409889103	-0.4913223588
H71	-3.1882069375	-0.1755374723	-2.2185418394
C72	0.1996951481	-2.6280648975	0.0197290823
H73	-0.3073708341	-3.6004373441	0.0107858619
H74	0.8524191967	-2.5906413809	-0.8601407562
C75	-0.3321532955	2.3786461845	0.5413040625
H76	-1.0270012415	3.2020832211	0.7444678178
H77	0.2257076137	2.6550282103	-0.3617623314
H78	2.3203588551	0.1577580513	0.4911847774

TS11-12

H1	0.4969643371	0.0068664513	2.2873392534
Ir2	1.7968847787	0.3534490254	1.4091914398
P3	1.9427177918	-1.9730391640	1.0377306472
P4	1.3679885112	2.6188111692	1.2215236006
C5	3.6498034693	-2.7290304188	0.5450276089
C6	4.2031364871	-1.8584571360	-0.6041265104
H7	4.2944720453	-0.8123753903	-0.3166848485
H8	3.5772520692	-1.9135140163	-1.5005799427
H9	5.2001935337	-2.2260848577	-0.8781302660
C10	3.6150937523	-4.1831961262	0.0280989640
H11	4.6035712136	-4.4324525942	-0.3786367767
H12	2.8930988498	-4.3242037381	-0.7823049590
H13	3.3957443426	-4.9117582910	0.8072587941
C14	4.6166583493	-2.6316251958	1.7414022982
H15	4.3599260749	-3.3388407976	2.5355141674
H16	4.6254823527	-1.6249669347	2.1700669154
H17	5.6353801075	-2.8706965522	1.4106074256
C18	1.1200926433	-3.0478213216	2.4253505960
C19	1.2726278884	-4.5709518572	2.2446025644
H20	2.2883438796	-4.9143891155	2.4540101209
H21	0.9899404446	-4.9088552602	1.2423705476
H22	0.6117374687	-5.0792919527	2.9584795213
C23	1.7063827134	-2.6395659602	3.7937810864
H24	1.2027007305	-3.2124638744	4.5833137357

H25	1.5569340417	-1.5750880890	3.9843009655
H26	2.7767112884	-2.8477915163	3.8704521521
C27	-0.3951462191	-2.7376206498	2.4499677109
H28	-0.8201770566	-3.1578531631	3.3696515996
H29	-0.9283068611	-3.1984289909	1.6129388706
H30	-0.5968267541	-1.6643327718	2.4450399807
C31	3.0623522454	2.9628257807	0.4356165117
C32	3.3667243209	1.4837690378	0.0404865444
H33	4.4487239323	1.3083545791	0.0524901022
H34	3.0120521463	1.2279431075	-0.9564992798
H35	3.3921024756	0.6498022485	1.4683006405
C36	4.1004637785	3.4241956513	1.4737105479
H37	4.1291142282	2.7618283684	2.3428283342
H38	3.9192546838	4.4474088061	1.8175845091
H39	5.0945420164	3.4088374256	1.0084083895
C40	3.0897427109	3.9257564063	-0.7607121297
H41	2.8590780116	4.9543939716	-0.4587247533
H42	2.3866309665	3.6372997877	-1.5462870370
H43	4.0937494622	3.9305133416	-1.2043240155
C44	0.8025101632	3.8357907227	2.5685968589
C45	1.6729590141	3.6606273709	3.8295413990
H46	1.2461527378	4.2559251874	4.6463584105
H47	2.6999866680	3.9985448463	3.6799543733
H48	1.7024405513	2.6137176929	4.1445847542
C49	0.8557544327	5.2937955995	2.0730297623
H50	0.2334676702	5.4490645180	1.1853726885
H51	1.8759220032	5.6084283171	1.8316811124
H52	0.4822971652	5.9658285756	2.8566083080
C53	-0.6495268311	3.4631353352	2.9432015771
H54	-0.7392074472	2.4006824474	3.1921901999
H55	-1.3603651982	3.6924789118	2.1442831371
H56	-0.9519056787	4.0421706900	3.8242663450
C57	-0.3853440766	-1.0465068974	-2.3523030415
C58	0.7031389135	-0.9263958350	-1.2601429765
C59	0.4002844905	0.2440274112	-0.3062888937
C60	0.2774179515	1.5600279762	-1.1030953465
C61	-0.8426541944	1.4645418366	-2.1600693180
C62	-0.5868726688	0.2791262008	-3.1089164390
H63	-1.3318115795	-1.3370233895	-1.8718704216
H64	-0.1395034068	-1.8458151885	-3.0657398125
H65	1.6555611699	-0.7081459506	-1.7683109492
H66	-0.5888770673	0.0533845948	0.1388836511
H67	1.2102967490	1.7114366294	-1.6656764847
H68	-1.8086878069	1.3309781183	-1.6506492189
H69	-0.9181624684	2.3947614368	-2.7409014336
H70	0.3127716327	0.4912711651	-3.7053337759
H71	-1.4127135859	0.1813500153	-3.8250154108
C72	0.1060705242	2.7572641472	-0.1503349957
H73	0.1540513691	3.7222436125	-0.6689739723
H74	-0.8786818523	2.6925720717	0.3264753358
C75	0.8714019069	-2.2458612030	-0.4838478637
H76	1.2679281299	-3.0439201079	-1.1205928394
H77	-0.1113179045	-2.5822857938	-0.1398558131
H78	2.3952407578	0.3495112644	2.9406995024

12

H1	1.6588523795	0.0022043654	3.1614052002
Ir2	1.5810009707	0.0022043610	1.5928526751
P3	1.3632349687	-2.3158003726	1.4140410198
P4	1.3661977502	2.3202000272	1.4136379405
C5	2.9152770200	-3.2639749276	0.7938686564
C6	3.5690100160	-2.3414854914	-0.2606659219
H7	3.8301448233	-1.3724265554	0.1740573563
H8	2.9138325406	-2.1602375395	-1.1190632922
H9	4.4835951587	-2.8159994567	-0.6394214653
C10	2.6106966446	-4.6234335945	0.1319546090
H11	3.5436917485	-5.0436618843	-0.2654719238
H12	1.9163300842	-4.5311523648	-0.7083358224
H13	2.1984928850	-5.3530523074	0.8311379253
C14	3.9238830911	-3.4624920922	1.9415519258
H15	3.5905544267	-4.2165530804	2.6604743774

H16	4.1114830649	-2.5273810196	2.4775785162
H17	4.8786190335	-3.8110635253	1.5276412285
C18	0.5694326379	-3.2028136676	2.9199924181
C19	0.4295090251	-4.7275874914	2.7553885790
H20	1.3969570290	-5.2360766146	2.7827293104
H21	-0.0791498483	-5.0054018216	1.8264892142
H22	-0.1680143341	-5.1268063680	3.5854215815
C23	1.3765983713	-2.8980084490	4.1992361578
H24	0.8518163175	-3.3262177471	5.0629166744
H25	1.4756591083	-1.8217588756	4.3570986877
H26	2.3796484036	-3.3292581308	4.1784055194
C27	-0.8387779623	-2.5912863231	3.0944302514
H28	-1.2791615977	-2.9670830516	4.0265709831
H29	-1.5140447132	-2.8668860874	2.2790090446
H30	-0.7925858504	-1.4996192649	3.1527318796
C31	2.9222251327	3.2675686444	0.8029888034
C32	3.5827039933	2.3431865451	-0.2457682439
H33	4.4992754937	2.8172895144	-0.6201703300
H34	2.9326191100	2.1597722452	-1.1075980400
H35	3.8415998393	1.3751312862	0.1925282063
C36	3.9234675272	3.4674826804	1.9568012492
H37	4.1077615230	2.5329810598	2.4950799486
H38	3.5854277606	4.2223269422	2.6726756925
H39	4.8808161243	3.8155769127	1.5485823921
C40	2.6215682438	4.6260970941	0.1372877348
H41	2.2027461333	5.3558849140	0.8324214033
H42	1.9342032122	4.5321402302	-0.7085118339
H43	3.5572767259	5.0469264277	-0.2530692476
C44	0.5620494468	3.2052123323	2.9157978418
C45	1.3634153319	2.9031959771	4.1992992561
H46	0.8327030373	3.3296048772	5.0602488344
H47	2.3651644149	3.3375869572	4.1837304240
H48	1.4649609200	1.8272419360	4.3576888783
C49	0.4176204704	4.7294016688	2.7498078924
H50	-0.0882458433	5.0047224403	1.8186544406
H51	1.3831096638	5.2413968199	2.7807533713
H52	-0.1849165952	5.1270215351	3.5770024449
C53	-0.8450286155	2.5893808136	3.0844125827
H54	-0.7960490342	1.4977234022	3.1409210705
H55	-1.5184233789	2.8646725893	2.2674524128
H56	-1.2895444172	2.9622777251	4.0157795537
C57	-1.5156142206	1.2833324744	-1.4819673598
C58	-0.7103491844	1.2771751613	-0.1642361725
C59	0.1554281622	0.0033151530	-0.0387632241
C60	-0.7175436781	-1.2661281526	-0.1556179583
C61	-1.5268322131	-1.2720144171	-1.4706048343
C62	-2.3667647811	0.0087243637	-1.6193323305
H63	-0.8137724427	1.3475904183	-2.3276724726
H64	-2.1628178232	2.1697527134	-1.5434895469
H65	-1.4415486660	1.2568067691	0.6565008716
H66	0.8543211824	-0.0013989110	-0.9050632791
H67	-1.4449665210	-1.2397449632	0.6684186949
H68	-0.8273817515	-1.3497679673	-2.3172193139
H69	-2.1823695909	-2.1528537528	-1.5248676058
H70	-3.1479583457	0.0157461202	-0.8449590198
H71	-2.8911820327	0.0059970163	-2.5834703185
C72	0.1365282066	-2.5367603586	0.0046651009
H73	-0.4748040604	-3.4398367309	0.1183831621
H74	0.7317330614	-2.6778375253	-0.9058550626
C75	0.1493972577	2.5432770724	-0.0036464757
H76	-0.4576578490	3.4500458467	0.1030381134
H77	0.7508549869	2.6774535352	-0.9111022225
H78	3.0589364078	0.0008006672	2.2593812893

7

H1	2.6514796000	-0.0065106337	0.4770764011
Ir2	1.5316526273	-0.0012617650	1.6723703374
P3	1.3681233426	-2.3399664660	1.4477549205
P4	1.3763068428	2.3368898644	1.4382446267
C5	2.9794070016	-3.2304877592	0.8504052218
C6	3.3238165197	-2.7168202163	-0.5672115656

H7	3.2882965493	-1.6269047034	-0.6279675168
H8	2.6637096079	-3.1328156933	-1.3342046056
H9	4.3429986137	-3.0373256223	-0.8149685187
C10	2.8680568065	-4.7659019088	0.7714254074
H11	3.7658809063	-5.1611136398	0.2789206369
H12	2.0050057304	-5.0931445822	0.1823822040
H13	2.8139041438	-5.2332871190	1.7576809921
C14	4.1523491318	-2.8499665884	1.7791997684
H15	4.0060184101	-3.1875050379	2.8074088445
H16	4.3034673755	-1.7690418547	1.7966494151
H17	5.0697140766	-3.3220726428	1.4048894147
C18	0.5816392745	-3.3229170958	2.9141836149
C19	0.0439076914	-4.7189783642	2.5308305372
H20	0.8103649151	-5.3944981097	2.1535791942
H21	-0.7566506106	-4.6628724941	1.7874617783
H22	-0.3872406783	-5.1834931631	3.4265653695
C23	1.6084117348	-3.4616166052	4.0561640920
H24	1.1015886375	-3.8353502199	4.9547621544
H25	2.0644430582	-2.4986874362	4.3053147717
H26	2.4042511067	-4.1722875127	3.8163905770
C27	-0.6177246992	-2.5095389208	3.4486052237
H28	-1.0870311712	-3.0759555182	4.2633341968
H29	-1.3797362089	-2.3429943026	2.6809422044
H30	-0.3093107309	-1.5371394171	3.8321413756
C31	2.9886037292	3.2173037256	0.8289295712
C32	3.3238882534	2.6946290990	-0.5875453343
H33	4.3434065519	3.0089279197	-0.8417926528
H34	2.6621929265	3.1098575773	-1.3535702119
H35	3.2829182225	1.6046048690	-0.6425302358
C36	4.1640542570	2.8367916407	1.7545355128
H37	4.3114252453	1.7554379422	1.7764886719
H38	4.0230860675	3.1795833028	2.7817786388
H39	5.0815666737	3.3039096696	1.3743420201
C40	2.8837658269	4.7528028993	0.7423197273
H41	2.8400263911	5.2256590781	1.7264947443
H42	2.0175291329	5.0811808034	0.1585774440
H43	3.7794683681	5.1410158182	0.2404910479
C44	0.6007810097	3.3284140931	2.9043812305
C45	1.6301305805	3.4577197459	4.0450486512
H46	1.1280277519	3.8358603286	4.9444575605
H47	2.4318314532	4.1613512688	3.8040343097
H48	2.0780192034	2.4907591636	4.2932752920
C49	0.0757273725	4.7294363439	2.5217260784
H50	-0.7216079219	4.6817995568	1.7743023848
H51	0.8494305856	5.3998074093	2.1502442510
H52	-0.3562091761	5.1950495900	3.4165171543
C53	-0.6058241991	2.5260617812	3.4392563473
H54	-0.3064300824	1.5504362576	3.8216902666
H55	-1.3699831175	2.3675039147	2.6719377761
H56	-1.0692074632	3.0961832797	4.2547870466
C57	-1.5440215288	1.2778037825	-1.4179333160
C58	-0.6886551447	1.2600044110	-0.1322760884
C59	0.1928608620	-0.0018925500	-0.0842678601
C60	-0.6899172648	-1.2629587526	-0.1303087148
C61	-1.5450041975	-1.2821230575	-1.4160947156
C62	-2.3920967523	-0.0019234408	-1.5392988294
H63	-0.8776007754	1.3616870698	-2.2896178342
H64	-2.1991390424	2.1599661834	-1.4388680023
H65	-1.3809378257	1.2090766076	0.7213130110
H66	0.8169628668	-0.0030249097	-0.9891655865
H67	-1.3824802597	-1.2099685895	0.7228988876
H68	-0.8785321448	-1.3677445517	-2.2875696160
H69	-2.2008559888	-2.1637642295	-1.4359487874
H70	-3.1526275860	-0.0011499154	-0.7449082790
H71	-2.9410847203	-0.0023660178	-2.4895432078
C72	0.1557140207	-2.5391369471	0.0248405268
H73	-0.4678993694	-3.4314342232	0.1373659090
H74	0.7459747251	-2.6838278837	-0.8852123769
C75	0.1575151557	2.5362221242	0.0205075708
H76	-0.4663331153	3.4278366398	0.1374359781
H77	0.7430803600	2.6830543847	-0.8922110598

H78	3.0293684774	-0.0016633510	2.3019947247
H79	1.5914720949	0.0007330644	3.2955570212
H80	0.0211480086	0.0031037177	2.2870695543

TS8-22

H1	2.4695408247	-0.1061976041	2.6930027529
Ir2	1.5481936944	0.0317753445	1.3274793715
P3	1.3925573904	2.3361247955	1.0600965553
P4	1.6061719766	-2.1733170119	0.7367754412
C5	1.9953185322	3.6543801785	2.2947686729
C6	1.1359468781	3.4404550158	3.5620651760
H7	1.1384466274	2.3908332515	3.8729200123
H8	0.0992163955	3.7604748142	3.4167727209
H9	1.5531302476	4.0392635360	4.3810630316
C10	1.8626309172	5.1165323856	1.8342535271
H11	2.0883239700	5.7835117815	2.6765721122
H12	0.8515468218	5.3567469777	1.4890964221
H13	2.5670600644	5.3613025590	1.0343549521
C14	3.4636755565	3.3421210690	2.6540587067
H15	4.1415813935	3.5071581912	1.8119408377
H16	3.5727047547	2.3044311598	2.9816132735
H17	3.7846326016	4.0026092496	3.4697645853
C18	1.9378014277	2.6724135172	-0.7996771803
C19	1.4103819433	4.0042889853	-1.3745013226
H20	1.7951234105	4.8739143335	-0.8379335065
H21	0.3176581163	4.0559643468	-1.3656550212
H22	1.7293173701	4.0976398646	-2.4199550802
C23	3.4777608251	2.6577357081	-0.8697269702
H24	3.8036520891	2.7072570858	-1.9160494539
H25	3.8853962580	1.7448029563	-0.4245873534
H26	3.9170197652	3.5156537686	-0.3504042059
C27	1.3583763918	1.5112860868	-1.6293780495
H28	2.1109114271	0.9138201604	-2.1496627987
H29	0.5353460333	1.7750746185	-2.2906109073
H30	1.3117724977	0.4827127871	-0.4784081222
C31	0.7434811669	-3.5002308613	1.8618264558
C32	-0.3061107061	-2.7454115888	2.7079655616
H33	-0.7900750417	-3.4507154551	3.3969892035
H34	-1.0896516368	-2.2873131091	2.1016707040
H35	0.1634280730	-1.9468939036	3.2889537907
C36	1.7384205881	-4.1224609756	2.8627456361
H37	2.2779947610	-3.3554656101	3.4254029421
H38	2.4647338616	-4.7847417843	2.3859279041
H39	1.1765619228	-4.7277019211	3.5858707811
C40	0.0522017181	-4.6274381504	1.0710219717
H41	0.7442804742	-5.1710328890	0.4209903076
H42	-0.7700278875	-4.2545451336	0.4535853070
H43	-0.3774578699	-5.3544248391	1.7727786819
C44	3.3519332748	-2.8127504117	0.1990196756
C45	4.3297237794	-2.7390542927	1.3930557418
H46	5.3495532882	-2.9213598006	1.0305423742
H47	4.1241327821	-3.4810644041	2.1641209571
H48	4.2976115605	-1.7480313429	1.8543148804
C49	3.3307711467	-4.2304235809	-0.4022438153
H50	2.6502579130	-4.3042084340	-1.2578562862
H51	3.0414125497	-4.9919268120	0.3268198035
H52	4.3343430117	-4.4924631681	-0.7628656184
C53	3.9013694474	-1.8298741231	-0.8622531336
H54	3.8363389487	-0.7978437536	-0.5044652523
H55	3.3847152764	-1.8983502192	-1.8244104673
H56	4.9556871183	-2.0670834403	-1.0509491523
C57	-1.7518419165	-1.4403494193	-0.6137911138
C58	-0.3334906498	-0.9800789241	-1.0372991872
C59	0.1225943500	0.2559005394	-0.2360791338
C60	-0.9279750335	1.3798901935	-0.0741522667
C61	-2.2524963326	0.7794465858	0.4409949870
C62	-2.7690046460	-0.3015029189	-0.5118229528
H63	-1.6919681937	-1.9374461020	0.3588101460
H64	-2.0904221616	-2.2000264682	-1.3298247800
H65	-0.4109455829	-0.7058978341	-2.1016026472
H66	-1.1692234234	1.8085234387	-1.0581384300

H67	-2.0916607071	0.3546217202	1.4405524480
H68	-2.9854747443	1.5897268228	0.5467012977
H69	-2.9472592404	0.1380315970	-1.5041211958
H70	-3.7336894747	-0.6932180100	-0.1665435245
C71	-0.4451149242	2.5305250496	0.8567688150
H72	-0.7601602971	3.5098124902	0.4792426825
H73	-0.8882116607	2.4041806289	1.8487901870
C74	0.6772365937	-2.1508517667	-0.9238634282
H75	1.4552191456	-2.0188480496	-1.6788086065
H76	0.1902559677	-3.1031911466	-1.1547502047

TS9-22

Ir1	1.4774729792	0.8396269345	0.8719705464
P2	1.1717079659	3.1473005855	0.6085437363
P3	1.3611612835	-1.4283060374	1.0082651931
C4	0.4263806360	4.0685140507	2.1312762205
C5	-0.9333518687	3.3888817505	2.4168971027
H6	-0.8360611000	2.3025730703	2.4815377872
H7	-1.6764683880	3.6130101674	1.6449598779
H8	-1.3291311962	3.7622896325	3.3699281064
C9	0.1781594768	5.5764455416	1.9389776761
H10	-0.4248378673	5.9522195414	2.7762305849
H11	-0.3695867441	5.8010609067	1.0180337014
H12	1.1083523924	6.1499124262	1.9336275563
C13	1.3349879277	3.8620202867	3.3602717215
H14	2.2956384394	4.3725591143	3.2580956530
H15	1.5342639332	2.8037147991	3.5378405210
H16	0.8401228535	4.2695827368	4.2512805276
C17	2.6037338482	4.1758384011	-0.1746794515
C18	2.1815023346	5.5588149846	-0.7136071027
H19	1.8984421144	6.2565125959	0.0747677908
H20	1.3516397408	5.4957205584	-1.4243566067
H21	3.0297122413	6.0034920810	-1.2503362569
C22	3.7436766839	4.3461360376	0.8469117250
H23	4.6274080602	4.7585183562	0.3438047649
H24	4.0251314627	3.3866657984	1.2911089514
H25	3.4777888868	5.0393246099	1.6501018656
C26	3.1444987528	3.3521194186	-1.3647444677
H27	3.9852977429	3.8948788916	-1.8163351716
H28	2.3910388327	3.2057283031	-2.1453873441
H29	3.4876532783	2.3668352320	-1.0455222431
C30	1.3366272334	-1.3893126263	2.9335065598
C31	1.3195617115	0.1633169016	3.1196709309
C32	2.5988668021	-1.9648974055	3.5964010567
H33	3.5053765362	-1.4684186920	3.2422679453
H34	2.7051697095	-3.0425006235	3.4398700258
H35	2.5339259860	-1.7968929458	4.6792909071
C36	0.0965237060	-2.0315804952	3.5728138493
H37	0.0553240663	-3.1115681764	3.3868040104
H38	-0.8338157785	-1.5857023431	3.2085364562
H39	0.1230705921	-1.8825177899	4.6597344049
C40	2.5332380185	-2.7191350446	0.2287354150
C41	3.9995583924	-2.4265551511	0.6032379683
H42	4.6589696323	-3.0278565785	-0.0349589673
H43	4.2281278317	-2.6848002466	1.6383110201
H44	4.2421464237	-1.3711877880	0.4468986699
C45	2.1468805546	-4.1553947982	0.6323937260
H46	1.1185966797	-4.4023746724	0.3476738709
H47	2.2485451617	-4.3244044947	1.7086948880
H48	2.8054225790	-4.8721320802	0.1249063748
C49	2.4038998309	-2.5560103694	-1.3013413354
H50	2.6199536214	-1.5282018569	-1.6083157492
H51	1.4106111339	-2.8240340564	-1.6705817092
H52	3.1256082038	-3.2195292389	-1.7935896069
C53	-0.1720490666	3.2528659222	-0.6928171746
H54	0.3037763636	3.6103061548	-1.6111737872
H55	-0.9193038136	4.0091700196	-0.4300461206
C56	-0.3389956050	-1.8096094488	0.3137719795
H57	-0.3330635726	-2.7411626687	-0.2612195589
H58	-1.0143452411	-1.9776149569	1.1564047223
H59	2.4442626102	0.7912534846	2.2809530853

H60	1.9395537416	0.4550602219	3.9754732058
H61	0.3174274317	0.5435146141	3.3129151855
H62	3.0742632597	0.8879334748	0.4842892844
C63	-0.1247619318	0.7166834885	-0.2319680618
C64	-0.9094064111	1.6008381029	-2.4779740931
C65	-0.8230276363	1.8739703816	-0.9509870544
C66	-0.8459799665	-0.6102878572	-0.5360264474
C67	-0.8843568398	-0.8983807710	-2.0571888551
C68	-1.5693301838	0.2550832617	-2.8022671533
H69	0.1132090779	1.6103091749	-2.8808169901
H70	-1.4541173182	2.4178297308	-2.9704669762
H71	-1.8635503950	1.9092872598	-0.5799686731
H72	-1.9002882658	-0.4520553880	-0.2452983283
H73	0.1401562860	-1.0194632123	-2.4302910545
H74	-1.4141989830	-1.8408966379	-2.2530503940
H75	-2.6304903258	0.2901191305	-2.5146004449
H76	-1.5482551360	0.0763844861	-3.8848685849

22

H1	3.1805990990	0.0014301603	1.6074823695
Ir2	1.5456763707	0.0007133316	1.1208971889
P3	1.4418004880	2.3042488901	0.9599011448
P4	1.4419508257	-2.3031231007	0.9622379986
C5	1.3583903197	3.2969406147	2.5993512140
C6	0.5034492767	2.4425564512	3.5626467541
H7	0.9376100552	1.4488431374	3.7028896791
H8	-0.5206082216	2.3066011136	3.2009329600
H9	0.4477025431	2.9443026531	4.5376121668
C10	0.7059187944	4.6887226175	2.4733491537
H11	0.6294673211	5.1393679918	3.4713425188
H12	-0.3085904168	4.6368996132	2.0664483532
H13	1.2839879903	5.3739355850	1.8510443430
C14	2.7672732788	3.4379528312	3.2079314346
H15	3.3911196858	4.1411748572	2.6487715352
H16	3.2848638366	2.4750692566	3.2511479949
H17	2.6824894030	3.8236473588	4.2318035153
C18	2.6793403138	3.1022320880	-0.2734242944
C19	2.5702845036	4.6340544049	-0.3846766010
H20	2.9189309107	5.1394285279	0.5199485662
H21	1.5485291445	4.9689695520	-0.5933643291
H22	3.2020543985	4.9829743030	-1.2121151804
C23	4.1234711688	2.7086247510	0.1006664813
H24	4.8066143702	3.0643323226	-0.6818394899
H25	4.2221701679	1.6241322399	0.1882807572
H26	4.4480537294	3.1511077826	1.0451843089
C27	2.3692822086	2.4812526903	-1.6542000851
H28	3.1325720158	2.8049064128	-2.3728628634
H29	1.3974687090	2.7988211862	-2.0454833331
H30	2.3848495794	1.3882952987	-1.6063155363
C31	1.3732211590	-3.2988573542	2.6013645337
C32	0.5308124885	-2.4422299462	3.5740890025
H33	0.4816566197	-2.9448785958	4.5489518381
H34	-0.4958660750	-2.3009553004	3.2218798857
H35	0.9708376974	-1.4506967086	3.7117884601
C36	2.7877249856	-3.4444410242	3.1958476462
H37	3.3075574127	-2.4826030992	3.2363400079
H38	3.4048625646	-4.1476989694	2.6293734971
H39	2.7118196957	-3.8319299235	4.2197567540
C40	0.7144560557	-4.6878759195	2.4801934167
H41	1.2828305317	-5.3740326697	1.8500212946
H42	-0.3044399787	-4.6311954454	2.0850090010
H43	0.6476829516	-5.1398591083	3.4782677887
C44	2.6714580999	-3.0992110144	-0.2801885107
C45	4.1169647825	-2.6999689896	0.0822414737
H46	4.7956519532	-3.0560441954	-0.7039202798
H47	4.4496376171	-3.1381269278	1.0259409485
H48	4.2126043926	-1.6148501836	0.1656421087
C49	2.5659967856	-4.6315173316	-0.3870645604
H50	1.5437557124	-4.9693875031	-0.5886326550
H51	2.9214204643	-5.1338308676	0.5166222790
H52	3.1935024091	-4.9807503045	-1.2175909784

C53	2.3491842480	-2.4826480040	-1.6601155336
H54	2.3619041710	-1.3895234647	-1.6151594206
H55	1.3754558639	-2.8042064693	-2.0432179656
H56	3.1080502554	-2.8058133436	-2.3836529781
C57	-2.2748079169	-1.2489847481	-0.5455216308
C58	-0.7267424738	-1.2745623308	-0.4949945137
C59	-0.1085944245	0.0003089995	0.1067329157
C60	-0.7225483298	1.2734549297	-0.5030466853
C61	-2.2706511547	1.2526344830	-0.5567103355
C62	-2.8105365869	-0.0003554181	-1.2475533271
H63	-2.6601624163	-1.2780627846	0.4840368996
H64	-2.6360684743	-2.1603739176	-1.0390124230
H65	-0.3813523152	-1.3004066186	-1.5438569171
H66	-0.3744522455	1.2924096044	-1.5512202924
H67	-2.6576932027	1.2929489716	0.4718325517
H68	-2.6275471734	2.1608174370	-1.0592690805
H69	-2.5041538184	-0.0056314662	-2.3035503969
H70	-3.9077089172	0.0017198715	-1.2404427995
C71	-0.2411570019	2.5650087171	0.1925913017
H72	-0.2792489122	3.4279188119	-0.4818913572
H73	-0.9238046429	2.7866155763	1.0203359767
C74	-0.2466736702	-2.5632446587	0.2069477785
H75	-0.2927188117	-3.4308578500	-0.4610184837
H76	-0.9247923008	-2.7755668488	1.0409389866

23

H1	1.0514030040	0.0006851737	2.7049614234
Ir2	1.5942459235	0.0003878121	1.1772682273
P3	1.4847166776	2.3272137891	1.0144765086
P4	1.4855540657	-2.3263585405	1.0150586106
C5	1.4016850566	3.3997781760	2.6120223398
C6	0.5525469473	2.6358883304	3.6509529421
H7	1.0242774602	1.6961378715	3.9395038898
H8	-0.4516831938	2.4025596813	3.2833194958
H9	0.4422528759	3.2647255133	4.5435960668
C10	0.7394079460	4.7780980035	2.3927594825
H11	0.6859652855	5.2999495019	3.3566093253
H12	-0.2858045570	4.6890651599	2.0206277972
H13	1.2929487693	5.4198693104	1.7076465640
C14	2.8148138109	3.5847925313	3.1991162112
H15	3.4320676148	4.2561265254	2.5956046026
H16	3.3346415038	2.6269124227	3.2971407669
H17	2.7360596051	4.0292436024	4.1992950090
C18	2.7164529035	3.1150171178	-0.2425440214
C19	2.6135516842	4.6473835120	-0.3709718334
H20	2.9765150473	5.1676342662	0.5188036107
H21	1.5930463331	4.9859294242	-0.5787935637
H22	3.2396949764	4.9747562970	-1.2110507717
C23	4.1659841517	2.7331641144	0.1245881407
H24	4.8384814924	3.0778969609	-0.6714945175
H25	4.2767698342	1.6515954654	0.2263073410
H26	4.4976222408	3.1957835311	1.0570964356
C27	2.3934841062	2.5039907636	-1.6251940768
H28	3.1600085255	2.8232073626	-2.3421304587
H29	1.4274798154	2.8460622163	-2.0100330106
H30	2.3914163022	1.4123445194	-1.5905790637
C31	1.4078345623	-3.3987850557	2.6129142350
C32	0.5630381519	-2.6344821944	3.6549810409
H33	0.4559458060	-3.2629869158	4.5483206507
H34	-0.4424990121	-2.4009313758	3.2912612068
H35	1.0363573347	-1.6948916352	3.9413238364
C36	2.8229971118	-3.5843209184	3.1947854721
H37	3.3436001814	-2.6266778640	3.2906154139
H38	3.4377281469	-4.2561011866	2.5893118180
H39	2.7476988074	-4.0283948625	4.1954125324
C40	0.7439836879	-4.7766696854	2.3961621174
H41	1.2933586031	-5.4179295111	1.7072615832
H42	-0.2832227328	-4.6867103963	2.0299027446
H43	0.6957272207	-5.2993831002	3.3598313685
C44	2.7143736959	-3.1138144631	-0.2450073117
C45	4.1647345970	-2.7316077786	0.1183559898

H46	4.8351981941	-3.0763339689	-0.6794562736
H47	4.4990400783	-3.1938529655	1.0500995128
H48	4.2755124353	-1.6499603547	0.2194507295
C49	2.6112994486	-4.6461962439	-0.3731757103
H50	1.5903336954	-4.9847326374	-0.5786942311
H51	2.9762654469	-5.1664781849	0.5157411773
H52	3.2355041305	-4.9735099291	-1.2146893271
C53	2.3881071206	-2.5028934455	-1.6269011782
H54	2.3855238886	-1.4112393275	-1.5922401687
H55	1.4214786269	-2.8455052037	-2.0096677777
H56	3.1532865043	-2.8216840568	-2.3454509181
C57	-2.2295849549	-1.2499536877	-0.4998381144
C58	-0.6816698734	-1.2809288856	-0.4680661553
C59	-0.0258202098	0.0000294100	0.0736137855
C60	-0.6807461701	1.2803875532	-0.4707019330
C61	-2.2286854022	1.2504675578	-0.5032112081
C62	-2.7692570831	-0.0004817525	-1.1979221462
H63	-2.6037870422	-1.2740381474	0.5340219441
H64	-2.6013362146	-2.1605424895	-0.9874674439
H65	-0.3511754866	-1.3390916317	-1.5198962381
H66	-0.3495006568	1.3363521526	-1.5224300630
H67	-2.6033308818	1.2777995483	0.5303929592
H68	-2.5994755622	2.1600111862	-0.9935267367
H69	-2.4661087491	-0.0020497923	-2.2551477478
H70	-3.8663466406	-0.0000163453	-1.1876791483
C71	-0.2046651295	2.5572644739	0.2548705021
H72	-0.2615658893	3.4378461368	-0.3947787376
H73	-0.8823280512	2.7456459287	1.0947825389
C74	-0.2057219162	-2.5566299755	0.2597171233
H75	-0.2653074460	-3.4387919236	-0.3875624071
H76	-0.8816408231	-2.7417765059	1.1017982071
H77	2.9381007192	0.0005891272	2.1887169230
H78	2.9186955486	0.0004615793	0.2296600268

TS23-12

H1	0.0000000000	0.0000000000	1.8135980000
Ir2	1.5910500000	0.0000000000	1.1890500000
P3	1.5066290000	2.3163420000	0.9926340000
P4	1.5069330000	-2.3165390000	0.9927870000
C5	1.4260000000	3.3679580000	2.5994380000
C6	0.5504930000	2.5842000000	3.6027260000
H7	0.9645430000	1.5949080000	3.8029620000
H8	-0.4753790000	2.4493820000	3.2442500000
H9	0.4989690000	3.1492890000	4.5422450000
C10	0.7995910000	4.7658690000	2.4097950000
H11	0.7169500000	5.2524350000	3.3900030000
H12	-0.2107120000	4.7152050000	1.9922580000
H13	1.3958030000	5.4202720000	1.7729320000
C14	2.8360860000	3.5062030000	3.2058460000
H15	3.4764670000	4.1715600000	2.6196170000
H16	3.3292640000	2.5334500000	3.2943930000
H17	2.7571660000	3.9367940000	4.2120000000
C18	2.7342750000	3.0873230000	-0.2717880000
C19	2.6188020000	4.6168650000	-0.4163600000
H20	2.9909190000	5.1454740000	0.4650100000
H21	1.5927380000	4.9471090000	-0.6093640000
H22	3.2299210000	4.9423280000	-1.2681820000
C23	4.1861290000	2.7196770000	0.0977560000
H24	4.8578200000	3.0817870000	-0.6911990000
H25	4.3078990000	1.6379770000	0.1857490000
H26	4.5088620000	3.1737880000	1.0374880000
C27	2.4139760000	2.4417120000	-1.6392350000
H28	3.1791680000	2.7438670000	-2.3652300000
H29	1.4455840000	2.7675180000	-2.0326060000
H30	2.4121260000	1.3508340000	-1.5714300000
C31	1.4257560000	-3.3697160000	2.5991800000
C32	0.5491100000	-2.5862830000	3.6018190000
H33	0.4979310000	-3.1507150000	4.5417540000
H34	-0.4768410000	-2.4525910000	3.2431440000
H35	0.9621830000	-1.5964670000	3.8015840000
C36	2.8355190000	-3.5060820000	3.2067030000

H37	3.3270570000	-2.5325340000	3.2958260000
H38	3.4775570000	-4.1703340000	2.6210290000
H39	2.7563180000	-3.9367840000	4.2127850000
C40	0.8004850000	-4.7678350000	2.4088400000
H41	1.3979470000	-5.4220610000	1.7730130000
H42	-0.2092660000	-4.7176840000	1.9898970000
H43	0.7167420000	-5.2541300000	3.3890730000
C44	2.7358680000	-3.0861760000	-0.2708550000
C45	4.1869630000	-2.7155160000	0.0988910000
H46	4.8598990000	-3.0787990000	-0.6884740000
H47	4.5095130000	-3.1664500000	1.0402000000
H48	4.3071080000	-1.6334310000	0.1840750000
C49	2.6229760000	-4.6159890000	-0.4139650000
H50	1.5975440000	-4.9480980000	-0.6071390000
H51	2.9955430000	-5.1429650000	0.4681740000
H52	3.2350860000	-4.9413490000	-1.2651060000
C53	2.4146270000	-2.4421830000	-1.6387300000
H54	2.4111210000	-1.3512490000	-1.5717450000
H55	1.4466630000	-2.7697200000	-2.0316900000
H56	3.1801840000	-2.7437360000	-2.3645810000
C57	-2.2477080000	-1.2523380000	-0.4159520000
C58	-0.7012200000	-1.2862240000	-0.4636840000
C59	0.0000000000	0.0000000000	0.0000000000
C60	-0.7016640000	1.2862860000	-0.4631680000
C61	-2.2480800000	1.2522360000	-0.4154500000
C62	-2.8206420000	0.0000240000	-1.0831540000
H63	-2.5666420000	-1.2769860000	0.6363780000
H64	-2.6481140000	-2.1602350000	-0.8860910000
H65	-0.4231180000	-1.3511580000	-1.5299780000
H66	-0.4236000000	1.3517100000	-1.5294610000
H67	-2.5671660000	1.2765810000	0.6368520000
H68	-2.6485170000	2.1602460000	-0.8854180000
H69	-2.5701050000	0.0002870000	-2.1542630000
H70	-3.9158350000	-0.0000990000	-1.0198100000
C71	-0.1878130000	2.5610240000	0.2427660000
H72	-0.2357300000	3.4321690000	-0.4195490000
H73	-0.8532370000	2.7770630000	1.0853070000
C74	-0.1870480000	-2.5611950000	0.2417600000
H75	-0.2343970000	-3.4319870000	-0.4211010000
H76	-0.8527940000	-2.7778730000	1.0839310000
H77	2.2879540000	-0.0000740000	2.6840340000
H78	3.1938120000	0.0002820000	0.9508320000

TS8-13

H1	-0.0653051292	-0.2425850509	0.9128956184
Ir2	1.4753918064	0.0831071640	1.3723016837
P3	0.8437065424	2.2811196035	1.0224014528
P4	1.8556811010	-2.0923112099	0.7611568771
C5	1.2156060968	3.8150802710	2.0786324565
C6	0.4364420597	3.6089752291	3.3968449740
H7	0.6551082045	2.6321796077	3.8405065001
H8	-0.6459575687	3.6887289169	3.2527480415
H9	0.7314913961	4.3826550862	4.1164416432
C10	0.8064493657	5.1549205121	1.4425185973
H11	0.9400647442	5.9628578087	2.1737844904
H12	-0.2433329694	5.1670808738	1.1312061488
H13	1.4239875479	5.4005273611	0.5739054892
C14	2.7246010447	3.8214711461	2.4000487382
H15	3.3353254785	3.9992402361	1.5099337445
H16	3.0368296871	2.8699732804	2.8399620251
H17	2.9413854710	4.6243325819	3.1163210059
C18	1.2989973502	2.4316726015	-0.8211013223
C19	2.8139367583	2.6402964621	-1.0056551433
H20	3.3884186629	1.9364327472	-0.3972690826
H21	3.1177514205	3.6583637978	-0.7377253900
H22	3.0868914983	2.4875554173	-2.0579509142
C23	0.8907165108	0.9951689931	-1.3407849327
H24	0.3863125855	1.1170098864	-2.3171749728
H25	2.6993485330	0.2188862334	2.4334335796
H26	1.8004729868	0.4223209910	-1.5273988142
C27	0.5583256587	3.5253883800	-1.6182987044

H28	0.8336072079	3.4483845482	-2.6782597608
H29	0.8299944321	4.5308707397	-1.2871425922
H30	-0.5296275433	3.4357751953	-1.5627678493
C31	1.1906623811	-3.5316268188	1.8895091881
C32	2.2723355328	-4.0165529874	2.8763499452
H33	3.0701611613	-4.5812674811	2.3883405532
H34	1.8046033785	-4.6879325837	3.6079768251
H35	2.7178674654	-3.1860817395	3.4313603299
C36	0.6506613862	-4.7415732242	1.1027293428
H37	1.4003715481	-5.1842117964	0.4404247198
H38	-0.2247919222	-4.4841797963	0.4999925953
H39	0.3362979323	-5.5220886126	1.8081932610
C40	0.0542992309	-2.9345772482	2.7486436656
H41	-0.7982688947	-2.6058760274	2.1511808603
H42	0.4059258871	-2.0686941426	3.3160321149
H43	-0.3088024029	-3.6996928978	3.4479818176
C44	3.6574393317	-2.5075229816	0.1884844199
C45	4.6421274365	-2.3458853231	1.3687810881
H46	5.6686341052	-2.3876855169	0.9825586856
H47	4.5491299536	-3.1309821671	2.1184110971
H48	4.5005537637	-1.3802033479	1.8619994790
C49	3.7973558932	-3.9056624061	-0.4424907871
H50	3.1201820646	-4.0450806793	-1.2922237121
H51	3.6107568539	-4.7091590027	0.2749756795
H52	4.8206483016	-4.0389548666	-0.8178252834
C53	4.0699528717	-1.4436047532	-0.8544242276
H54	3.9219595097	-0.4347141559	-0.4595455605
H55	3.5285520695	-1.5293528768	-1.8011444523
H56	5.1348238388	-1.5702680066	-1.0855104721
C57	-1.6270184171	-1.9092347304	-0.5950445464
C58	-0.3160632709	-1.2420376570	-1.0601098306
C59	-0.0678559158	0.1622632080	-0.4305766846
C60	-1.3435238834	1.0413288254	-0.1987975805
C61	-2.5698594244	0.2448190864	0.2730919658
C62	-2.8320578377	-0.9639180742	-0.6303196592
H63	-1.5126500623	-2.2822097857	0.4286506335
H64	-1.8093938389	-2.7907586222	-1.2233811666
H65	-0.4263310343	-1.0849112202	-2.1463382128
H66	-1.6084389353	1.4834911610	-1.1713674682
H67	-2.4060695634	-0.1034315530	1.3031320081
H68	-3.4416158633	0.9112056241	0.3048101371
H69	-3.0175209083	-0.6264220390	-1.6606592579
H70	-3.7360269473	-1.4951534236	-0.3083277855
C71	-1.0120873932	2.2011190366	0.7954201350
H72	-1.4402117400	3.1523923771	0.4605979703
H73	-1.4445965291	1.9770858697	1.7750385507
C74	0.8985911134	-2.2047807488	-0.8876274611
H75	1.6440336899	-1.9586088362	-1.6460241378
H76	0.5858409858	-3.2314788999	-1.1031367024

13

H1	0.0300547187	-0.1545588286	0.6129050773
Ir2	1.5830506315	0.0958119599	1.4165010135
P3	0.8520066745	2.2665866189	1.0443997045
P4	1.8688327777	-2.0804321264	0.7647236432
C5	1.1612367315	3.8326972175	2.0801494629
C6	0.4024382300	3.6036161931	3.4063536384
H7	0.6604872914	2.6371694898	3.8513509210
H8	-0.6833288171	3.6429129473	3.2714893005
H9	0.6750062548	4.3906618738	4.1202506244
C10	0.6947531261	5.1525927793	1.4425838682
H11	0.7936681791	5.9659648958	2.1733842579
H12	-0.3544513866	5.1211284494	1.1310660864
H13	1.3014455136	5.4241354279	0.5742947682
C14	2.6716247835	3.9010024554	2.3871565180
H15	3.2665073407	4.0908205255	1.4886555254
H16	3.0243719313	2.9673389418	2.8347716061
H17	2.8648276323	4.7198289059	3.0920483244
C18	1.2561199660	2.4315243186	-0.8123003149
C19	2.7645424115	2.6508359137	-1.0337174783
H20	3.3525083018	1.9415137421	-0.4445774613

H21	3.0686632488	3.6673360430	-0.7605964688
H22	3.0127470478	2.5123044906	-2.0942398671
C23	0.8576056335	0.9952198465	-1.3408337637
H24	0.3954962092	1.1187277719	-2.3358690569
H25	2.7183183961	0.2049743520	2.5204352787
H26	1.7712066543	0.4175855543	-1.4857516183
C27	0.4890329610	3.5243763720	-1.5861572584
H28	0.7187023525	3.4378400815	-2.6562482661
H29	0.7845228588	4.5290356075	-1.2747929300
H30	-0.5962925289	3.4484425058	-1.4836323789
C31	1.1574328568	-3.5018944640	1.8869747123
C32	2.2196096173	-3.9952548564	2.8903364323
H33	3.0082361521	-4.5851185502	2.4167925066
H34	1.7326805434	-4.6439361503	3.6299981193
H35	2.6797853594	-3.1649280806	3.4338802844
C36	0.6011634376	-4.7096403491	1.1084129840
H37	1.3467341312	-5.1746103486	0.4573464729
H38	-0.2643748300	-4.4425119169	0.4956853215
H39	0.2651862694	-5.4759982065	1.8193339711
C40	0.0225598084	-2.8715301113	2.7241560877
H41	-0.8061000889	-2.5141180937	2.1090397466
H42	0.3889713137	-2.0170718429	3.2994631395
H43	-0.3798401700	-3.6260071225	3.4136680250
C44	3.6412249808	-2.5706665743	0.1543080234
C45	4.6432846691	-2.4129821699	1.3202354588
H46	5.6634035724	-2.5153118149	0.9291819382
H47	4.5197276642	-3.1644928404	2.0990958983
H48	4.5480574326	-1.4250058038	1.7785367454
C49	3.7367671001	-3.9837968398	-0.4496772495
H50	3.0390038559	-4.1259229021	-1.2821504497
H51	3.5484946279	-4.7677961864	0.2886835411
H52	4.7486972038	-4.1476638676	-0.8436520842
C53	4.0670350386	-1.5365074878	-0.9131259270
H54	3.9197636640	-0.5172179386	-0.5438429159
H55	3.5316773229	-1.6466331337	-1.8609804268
H56	5.1326791053	-1.6737812992	-1.1342318290
C57	-1.6723833818	-1.9172998395	-0.6590672504
C58	-0.3584464268	-1.2513199422	-1.1150632992
C59	-0.1520580720	0.1620340663	-0.4992531058
C60	-1.4025796543	1.0482038888	-0.2188673813
C61	-2.6246709127	0.2315217732	0.2306889923
C62	-2.8719702615	-0.9630675131	-0.6980745315
H63	-1.5647492178	-2.2918523642	0.3648209716
H64	-1.8562633579	-2.7967753826	-1.2892456848
H65	-0.4400242900	-1.1195019844	-2.2059617225
H66	-1.6711384866	1.5449868319	-1.1619762238
H67	-2.4597178861	-0.1386792400	1.2525673363
H68	-3.5040084734	0.8865055071	0.2750244635
H69	-3.0404381169	-0.6092504534	-1.7259735617
H70	-3.7829221786	-1.4954599844	-0.3992124724
C71	-1.0215552366	2.1412923608	0.8376262848
H72	-1.4651747367	3.1089394259	0.5779726109
H73	-1.4241209091	1.8535683307	1.8131002869
C74	0.8807696091	-2.1800674881	-0.8878326215
H75	1.6273836487	-1.9329244319	-1.6441690995
H76	0.5898579341	-3.2155676059	-1.0910812310

TS13-10

H1	-0.2590786806	-0.3734837151	1.0801188175
Ir2	1.7012812341	0.0974619455	1.3779259550
P3	0.9515788494	2.2878845246	1.2873664667
P4	1.9219630156	-2.1342988303	0.8619454891
C5	1.2008150849	3.8159969955	2.3848313505
C6	0.2848178795	3.6383961693	3.6153873689
H7	0.4540267328	2.6732816189	4.1042770426
H8	-0.7759530788	3.7131152977	3.3560297641
H9	0.5013882976	4.4278452399	4.3458832264
C10	0.8647825193	5.1378028389	1.6715220575
H11	0.9571927699	5.9731220990	2.3780071865
H12	-0.1586608250	5.1521375304	1.2819165945
H13	1.5479781471	5.3368882372	0.8405700676

C14	2.6670531613	3.8246149556	2.8625487181
H15	3.3675858339	3.9884892691	2.0390723806
H16	2.9300154224	2.8770772405	3.3415175412
H17	2.8106117560	4.6353684330	3.5883220146
C18	1.5780713173	2.4254553635	-0.4981284607
C19	3.0934335788	2.7145529945	-0.5291149361
H20	3.6327756618	2.0491040702	0.1497913067
H21	3.3104308463	3.7530151347	-0.2540861397
H22	3.4779572515	2.5606682300	-1.5457838704
C23	1.3299196690	0.9174201229	-0.8547044995
H24	0.7200536331	0.8346322224	-1.7600368687
H25	2.6853644364	0.0664511175	2.6424272740
H26	2.2675157892	0.4006569234	-1.0628771065
C27	0.8751282607	3.4040173833	-1.4562325988
H28	1.2020944250	3.2053870622	-2.4855133632
H29	1.1371251867	4.4418892063	-1.2283012506
H30	-0.2138674025	3.3252662287	-1.4388198036
C31	1.3597496470	-3.3343567464	2.2672533241
C32	2.1693204177	-3.0314549330	3.5462686641
H33	3.2155910102	-3.3291068713	3.4622893353
H34	1.7368979080	-3.5934147603	4.3843848834
H35	2.1354907620	-1.9666633857	3.7841211345
C36	1.4665252706	-4.8324473637	1.9292222296
H37	2.5053260072	-5.1701838786	1.8788040387
H38	0.9748980383	-5.0907037049	0.9841845325
H39	0.9771940911	-5.4172615400	2.7195755160
C40	-0.1156130419	-3.0032432081	2.5752898834
H41	-0.7830818864	-3.2495757609	1.7445526642
H42	-0.2382937936	-1.9446695110	2.8205698767
H43	-0.4404719727	-3.5946375391	3.4405936041
C44	3.5684423302	-2.7774082609	0.0824132430
C45	4.6445494540	-2.8963909062	1.1799852236
H46	5.6278702991	-3.0284266690	0.7105994715
H47	4.4792129120	-3.7610946739	1.8286675868
H48	4.6839425117	-1.9961920692	1.8008862341
C49	3.4693412649	-4.1094704803	-0.6888654892
H50	2.7285291769	-4.0770136981	-1.4935292425
H51	3.2268841289	-4.9558710447	-0.0456469421
H52	4.4398139976	-4.3226513648	-1.1553365371
C53	4.0280799389	-1.6826629138	-0.9044163314
H54	4.1636448218	-0.7271261353	-0.3911361518
H55	3.3194097224	-1.5376138956	-1.7270721904
H56	4.9863667794	-1.9775795274	-1.3511009233
C57	-1.8328407867	-1.7828165788	-0.7233276617
C58	-0.3704823625	-1.2863997231	-0.8494156759
C59	-0.1769511887	-0.0360091510	0.0152844528
C60	-1.2220348072	1.0811819148	-0.0848805873
C61	-2.6413117306	0.4976524771	0.0636942691
C62	-2.8577735694	-0.6558471521	-0.9290354458
H63	-1.9825697081	-2.2198883389	0.2727031080
H64	-2.0078126865	-2.5891032860	-1.4477328776
H65	-0.2342083288	-1.0007142458	-1.9049208740
H66	-1.1805097403	1.5324735648	-1.0864019873
H67	-2.7817734713	0.1298968952	1.0900408030
H68	-3.3889519956	1.2861424814	-0.0952529756
H69	-2.7780702907	-0.2700673989	-1.9561807741
H70	-3.8742079498	-1.0557505031	-0.8288291776
C71	-0.9034491461	2.1765999535	0.9705104253
H72	-1.3276298753	3.1435760902	0.6749992363
H73	-1.3620113390	1.8997170630	1.9258312567
C74	0.6629495395	-2.4278871148	-0.5456882202
H75	1.2597999738	-2.6034450130	-1.4445910217
H76	0.1362107971	-3.3705546646	-0.3605736941

14

H1	3.3693647349	0.0624042676	2.1908152676
Ir2	1.9403973807	-0.0360935678	1.5657992692
P3	1.5051516040	-2.3067310127	1.2115997867
P4	1.2005224264	2.1654368351	1.7003063121
C5	2.7671234314	-3.7121363473	1.3877420994
C6	3.8208640137	-3.4648094509	0.2843575282

H7	4.1928602872	-2.4362798122	0.3104018306
H8	3.4206094044	-3.6611583092	-0.7157560596
H9	4.6691057566	-4.1427770247	0.4400537378
C10	2.1914288149	-5.1315631233	1.2425573789
H11	3.0156495527	-5.8564092443	1.2282972414
H12	1.6264084238	-5.2627487553	0.3139547617
H13	1.5417753998	-5.3979758094	2.0809177959
C14	3.4457101124	-3.5575126532	2.7650182097
H15	2.7492993146	-3.7361488757	3.5897947856
H16	3.8635827291	-2.5544223102	2.8877972028
H17	4.2603335327	-4.2876183359	2.8524458740
C18	-0.1691427567	-2.5863684500	2.0879765695
C19	-0.9661259283	-3.8171882493	1.5975472306
H20	-0.4733472310	-4.7533868777	1.8698834548
H21	-1.1370382142	-3.8318424781	0.5192203792
H22	-1.9514893984	-3.8210729791	2.0806452940
C23	-0.0217464577	-2.7085822993	3.6150036158
H24	-1.0106221277	-2.6454423627	4.0876035826
H25	0.6037246367	-1.9083524249	4.0164098057
H26	0.4105837158	-3.6745519283	3.8993302808
C27	-0.9404303953	-1.2485296200	1.7394451697
H28	-0.8949151574	-0.5886393134	2.6050885507
H29	-1.9997258854	-1.5082676938	1.5805690500
H30	1.4145387095	-0.2032017835	3.1344822734
C31	1.8970083661	3.5098115579	0.4781483314
C32	2.3237194525	2.8181090098	-0.8332489445
H33	2.7215710795	3.5808473539	-1.5154196142
H34	1.4958153107	2.3220485153	-1.3436956530
H35	3.0870702300	2.0606634100	-0.6530697186
C36	3.1839942635	4.1317652894	1.0636368393
H37	3.9028509345	3.3585163294	1.3519182866
H38	3.0021185254	4.7772876786	1.9249054865
H39	3.6597370309	4.7499652519	0.2917558900
C40	0.8839421772	4.6193856004	0.1290875346
H41	0.5056721573	5.1535349025	1.0022892175
H42	0.0267485079	4.2257732386	-0.4254616796
H43	1.3714915179	5.3582084245	-0.5199081171
C44	1.1206991962	2.9548344707	3.4813782136
C45	2.5264894617	2.8207267047	4.1085084694
H46	2.4942385353	3.1869585283	5.1426562203
H47	3.2883643147	3.3966179233	3.5811553238
H48	2.8377468144	1.7730726238	4.1203851731
C49	0.6484336579	4.4216204101	3.5226842271
H50	-0.3430911779	4.5470769634	3.0743269392
H51	1.3366579339	5.1120220944	3.0318471888
H52	0.5686263539	4.7383910454	4.5707028290
C53	0.1552302961	2.1423135450	4.3741315775
H54	0.3292410626	1.0681798582	4.2786143297
H55	-0.8967950222	2.3610108602	4.1649108245
H56	0.3324555375	2.4230728001	5.4194838695
C57	-1.2673020347	1.4035239467	-1.1369403869
C58	-1.2293203203	0.8905829889	0.3181943076
C59	-0.4875022033	-0.4680252146	0.4743193370
C60	-0.4307379546	-1.4638369930	-0.7227763762
C61	-0.4296842421	-0.7631724817	-2.0900088862
C62	-1.5341295258	0.2983835985	-2.1661085220
H63	-0.3126759475	1.8764075928	-1.3876288316
H64	-2.0270468194	2.1923712055	-1.2081276785
H65	-2.2762240985	0.7241326759	0.6176931376
H66	0.5830163857	-0.0853101695	0.3842441300
H67	-1.3387963622	-2.0808995898	-0.6768381912
H68	0.5438543184	-0.2813102811	-2.2537052157
H69	-0.5460469304	-1.5130113173	-2.8828517909
H70	-2.5146457675	-0.1657044199	-1.9830839682
H71	-1.5778448151	0.7317041202	-3.1724136913
C72	0.8233660832	-2.3911264904	-0.5448108955
H73	0.5925018101	-3.4204265894	-0.8406859483
H74	1.6274320062	-2.0316127169	-1.1917311035
C75	-0.6832194462	1.9737008505	1.3108703396
H76	-1.0980786134	1.7503557682	2.2938453790
H77	-1.0930986260	2.9497912712	1.0282887557

H78	2.7407070279	0.0652316781	0.1036320636

TS14-11

H1	3.3540931332	0.2015598092	1.9948975175
Ir2	1.8510006982	-0.0283349537	1.5935269388
P3	1.6281898414	-2.2990874542	1.1350727739
P4	1.1377080793	2.1926994767	1.6730893402
C5	2.9198190810	-3.6852184951	1.1666401705
C6	3.8380462119	-3.4729617014	-0.0561844053
H7	4.2273766176	-2.4505277216	-0.0920803272
H8	3.3223078266	-3.6785297324	-0.9995226289
H9	4.6916583208	-4.1588518997	0.0093401312
C10	2.2980125839	-5.0926454540	1.1171127794
H11	3.0955682825	-5.8451986066	1.0654393491
H12	1.6584187999	-5.2353249299	0.2397064883
H13	1.7041725109	-5.3080666859	2.0103607550
C14	3.7594601912	-3.5193713925	2.4498562210
H15	3.1673485223	-3.6841193074	3.3542743086
H16	4.1918911859	-2.5166625867	2.5111544890
H17	4.5768858365	-4.2516550127	2.4477638931
C18	0.1218414099	-2.5365721513	2.2397770867
C19	-0.8799310259	-3.6470612179	1.8618810105
H20	-0.4857627656	-4.6347627849	2.1198237457
H21	-1.1474616255	-3.6635107651	0.8040343653
H22	-1.8074113165	-3.5113796315	2.4328218181
C23	0.4817876179	-2.7274903922	3.7255304786
H24	-0.4288612269	-2.6406686872	4.3316227093
H25	1.1952659696	-1.9729535375	4.0614110576
H26	0.8984887321	-3.7241512172	3.9083215163
C27	-0.4457024230	-1.0933064904	2.0019858775
H28	-0.4796325272	-0.4983720324	2.9059627752
H29	-1.4534460390	-1.1472248827	1.5886355506
H30	1.7198373077	-0.2245450050	3.2322722269
C31	2.1347958837	3.4191542626	0.5548253435
C32	1.9167969063	2.9996864200	-0.9147242366
H33	2.5938083936	3.5831188447	-1.5511524450
H34	0.8980356295	3.2018444765	-1.2596956176
H35	2.1460275311	1.9412971276	-1.0625802851
C36	3.6363325220	3.2498064072	0.8714732209
H37	3.9437014228	2.2098266750	0.7419052235
H38	3.8873702210	3.5557126935	1.8903269033
H39	4.2204185215	3.8768995225	0.1853817673
C40	1.7476769769	4.9038594182	0.6862427721
H41	2.0582898586	5.3302873182	1.6430640347
H42	0.6732127005	5.0755502373	0.5619806664
H43	2.2594296613	5.4753355183	-0.0989351881
C44	0.8173231079	2.9972221923	3.4062846190
C45	2.1750460649	3.2422676675	4.0932544721
H46	2.0090813516	3.5247539493	5.1405831106
H47	2.7351411722	4.0560693891	3.6237949778
H48	2.7927784205	2.3392763752	4.0800068444
C49	-0.0039275025	4.3047815823	3.3908768273
H50	-0.9660937602	4.1917776740	2.8814701473
H51	0.5235140627	5.1400867288	2.9327847480
H52	-0.2247259839	4.5908018623	4.4270926137
C53	0.0331840760	1.9719359917	4.2548102377
H54	0.5765109018	1.0285113402	4.3333760327
H55	-0.9638712949	1.7683648324	3.8478301332
H56	-0.1087069572	2.3835718105	5.2621347720
C57	-1.5851017762	1.2496013547	-1.2836661682
C58	-1.1652797388	0.9365223212	0.1785477229
C59	-0.1413583468	-0.1846308732	0.1259015167
C60	-0.4306137408	-1.4534759891	-0.6621513453
C61	-0.8151367400	-1.0544099335	-2.1030508858
C62	-1.9574464861	-0.0189454879	-2.0759485575
H63	-0.7544965715	1.7548596732	-1.7938801787
H64	-2.4279466391	1.9531526670	-1.2834817662
H65	-2.0720867825	0.5965728958	0.7029025000
H66	0.7109445466	0.2459809922	-0.3873363838
H67	-1.3043830360	-1.9719465553	-0.2461423858
H68	0.0586089829	-0.6274080038	-2.6153051598

H69	-1.1183733159	-1.9400391219	-2.6768663945
H70	-2.8490581868	-0.4804257813	-1.6264342770
H71	-2.2379806385	0.2571785260	-3.0996282091
C72	0.8089548793	-2.3905021189	-0.5675363433
H73	0.5471204147	-3.4198782568	-0.8409141660
H74	1.5715743450	-2.0435657640	-1.2725320904
C75	-0.6330055144	2.2068596738	0.9331025091
H76	-1.2923079608	2.4031312704	1.7814633152
H77	-0.7176875389	3.0883940466	0.2884727080
H78	2.5516667071	0.1903039062	0.0789790922

15

H1	0.1754217850	-2.9962618007	-1.2486170802
Ir2	1.7328693709	0.0057352871	1.2783683692
P3	1.4566433436	2.3172609939	1.3168601881
P4	1.7488882918	-2.1587470395	0.5426037774
C5	2.0909166449	3.7524069250	2.3756489807
C6	1.2827804558	3.7380669173	3.6906457852
H7	1.3241101617	2.7571505179	4.1760615470
H8	0.2318862377	4.0002205389	3.5320414627
H9	1.7051105193	4.4744743289	4.3857114593
C10	1.9299393326	5.1132439602	1.6741935464
H11	2.2580182577	5.9175316669	2.3455529188
H12	0.8888310416	5.3180814253	1.4031382802
H13	2.5364023787	5.1770365055	0.7656862852
C14	3.5749200964	3.4903025335	2.7034107224
H15	4.2058625022	3.5223559943	1.8106914183
H16	3.7084506644	2.5121844746	3.1753116987
H17	3.9385729925	4.2602307207	3.3956936332
C18	1.9428424682	2.3271433297	-0.5165694786
C19	1.2954465746	3.3497208666	-1.4682259371
H20	1.6816393026	4.3604554774	-1.2957348278
H21	0.2073515662	3.3876513654	-1.3878450992
H22	1.5369404144	3.0819124615	-2.5050783339
C23	3.4707368525	2.3778804347	-0.7020497734
H24	3.7232147383	2.1091478397	-1.7362453993
H25	3.9762092971	1.6745853132	-0.0349373702
H26	3.8679986357	3.3831486827	-0.5236802799
C27	1.4666527495	0.8674311158	-0.7072332673
H28	1.9788994924	0.3381822708	-1.5116693086
H29	2.5074227312	-0.3483170734	2.6955409588
C30	0.9657080994	-3.5038860688	1.7025335014
C31	0.0153639736	-2.7708350682	2.6757115646
H32	-0.4341449199	-3.5049847379	3.3579058875
H33	-0.7977144461	-2.2509581118	2.1674799161
H34	0.5588876543	-2.0272815854	3.2638261026
C35	2.0304531148	-4.1888066782	2.5838759234
H36	2.6350444465	-3.4582655467	3.1282306683
H37	2.6948589219	-4.8471081800	2.0195664077
H38	1.5179400949	-4.8110724364	3.3286657916
C39	0.1804517058	-4.5839789455	0.9327943212
H40	0.7964728977	-5.1097316288	0.1971913499
H41	-0.6889301792	-4.1714032564	0.4137913874
H42	-0.1939041498	-5.3336598137	1.6419729393
C43	3.4345653077	-2.8116622690	-0.1477349642
C44	4.5049844783	-2.8002527824	0.9670390764
H45	5.4902401502	-2.9768690726	0.5167250403
H46	4.3524000393	-3.5719138984	1.7205138583
H47	4.5266710953	-1.8296401240	1.4711279214
C48	3.3326197763	-4.2067210448	-0.7932902383
H49	2.5917289452	-4.2321509603	-1.6000775851
H50	3.0746801181	-4.9867040051	-0.0725204205
H51	4.3007844402	-4.4799791170	-1.2335140726
C52	3.9290211262	-1.8091835719	-1.2157953213
H53	3.9682676101	-0.7943987662	-0.8121958716
H54	3.3154716219	-1.8018415598	-2.1213907975
H55	4.9424610195	-2.0958471895	-1.5225597266
C56	-1.7027645738	-1.3977626038	-0.4395239314
C57	-0.3472088871	-0.8888083326	-0.9734713715
C58	0.1884567160	0.3856287747	-0.2654267095
C59	-0.8954043784	1.4133144594	0.1518235428

C60	-2.1555936662	0.7580689749	0.7397058885
C61	-2.7312453643	-0.2887498407	-0.2147861209
H62	-1.5517058366	-1.9191963820	0.5100208749
H63	-2.0903690214	-2.1465189359	-1.1430106782
H64	-0.5300844964	-0.6005387511	-2.0236205572
H65	-1.2222634442	1.9107987468	-0.7765812538
H66	-1.9011916288	0.2858040593	1.6985715828
H67	-2.8966526746	1.5393929676	0.9537295019
H68	-2.9923803684	0.1876404088	-1.1715022002
H69	-3.6601600320	-0.7123341090	0.1867842353
C70	-0.3736421091	2.5228003569	1.1188203430
H71	-0.6521558938	3.5229214207	0.7668082075
H72	-0.8258419143	2.3894483139	2.1061984103
C73	0.6809395790	-2.0542192723	-1.0158787940
H74	1.3857356050	-1.8866725587	-1.8326781984

Benzene

C1	1.8266007311	0.0255336404	6.4277308727
C2	2.9961751504	0.0115887594	5.6650073879
C3	2.9200182716	-0.0144397918	4.2709649078
C4	1.6743899590	-0.0248749549	3.6397095011
C5	0.5049354489	-0.0120592008	4.4023546736
C6	0.5811815209	0.0139238684	5.7964360399
H7	1.8854789915	0.0442229022	7.5125708034
H8	3.9653513127	0.0196803475	6.1562687693
H9	3.8299552035	-0.0234512288	3.6772130006
H10	-0.4643023435	-0.0193062859	3.9112532138
H11	-0.3288469149	0.0234458121	6.3901306432
H12	1.6168689427	-0.0447659531	2.5548614588

2-*anti*

H1	0.6073392239	0.0016213786	2.6446371347
Ir2	1.6628995569	0.0018326614	1.5297897577
P3	1.4411407687	-2.3453969213	1.2791364422
P4	1.4427501583	2.3499005468	1.2814550563
C5	2.9548070204	-3.1667316534	0.4219681288
C6	3.4851708552	-2.0975381332	-0.5601896547
H7	3.8246398707	-1.2033570737	-0.0279874066
H8	2.7338302925	-1.7947563919	-1.2967997094
H9	4.3462688961	-2.4982405048	-1.1105506542
C10	2.6197397744	-4.4394128744	-0.3835069975
H11	3.5331547959	-4.7937420617	-0.8786279806
H12	1.8783079374	-4.2606274796	-1.1670257590
H13	2.2558701432	-5.2531878048	0.2458455389
C14	4.0668946812	-3.4895118263	1.4388077846
H15	3.8060321882	-4.3425809459	2.0713650281
H16	4.2993011562	-2.6360056952	2.0807236128
H17	4.9815144228	-3.7603984048	0.8955063581
C18	0.8235003643	-3.4168373538	2.7501213772
C19	0.6913262571	-4.9138531759	2.4060229902
H20	1.6645150624	-5.3922170360	2.2691545981
H21	0.0883834479	-5.0945627579	1.5105007137
H22	0.1956535095	-5.4272926590	3.2400962943
C23	1.7483615839	-3.2612563076	3.9732936841
H24	1.3012792726	-3.7917272220	4.8243224451
H25	1.8821069843	-2.2163532699	4.2535102748
H26	2.7400466276	-3.6870786417	3.8085658731
C27	-0.5738061769	-2.8816020696	3.1372968996
H28	-0.9198475922	-3.4119307323	4.0331841310
H29	-1.3176371074	-3.0462217606	2.3523639301
H30	-0.5488691385	-1.8132470335	3.3693071284
C31	2.9574961678	3.1697492742	0.4246687234
C32	3.4865985426	2.1000235925	-0.5576043711
H33	4.3483333732	2.4996490887	-1.1077614248
H34	2.7349963332	1.7983271413	-1.2943964846
H35	3.8248877039	1.2054080341	-0.0253568533
C36	4.0697622600	3.4904387144	1.4419330376
H37	4.3007058112	2.6362209381	2.0834403946
H38	3.8100169893	4.3436081015	2.0748155571
H39	4.9849178539	3.7601866011	0.8989946688
C40	2.6244142864	4.4432221565	-0.3804180104

H41	2.2615725551	5.2573100911	0.2491441838
H42	1.8828781369	4.2657628551	-1.1641507404
H43	3.5384390868	4.7963812185	-0.8752506982
C44	0.8265964097	3.4212842300	2.7531859897
C45	1.7516230654	3.2640688166	3.9759624593
H46	1.3049950453	3.7940372146	4.8275455123
H47	2.7435214252	3.6894894658	3.8114177630
H48	1.8848516120	2.2188421209	4.2552309382
C49	0.6961651527	4.9185691579	2.4097560838
H50	0.0939346178	5.1003315689	1.5139677072
H51	1.6699269149	5.3959981995	2.2737130397
H52	0.2005352306	5.4320732167	3.2437991729
C53	-0.5711312072	2.8871977919	3.1403481789
H54	-0.5471504407	1.8186401293	3.3716085439
H55	-1.3149675009	3.0530297047	2.3556704646
H56	-0.9164345268	3.4172436935	4.0366753076
C57	-1.7837560757	1.2775631523	-1.1906238077
C58	-0.7813715200	1.2644459685	-0.0163400729
C59	0.1091487015	0.0033527914	-0.0237990193
C60	-0.7820613071	-1.2572913172	-0.0176935950
C61	-1.7844788948	-1.2686781786	-1.1919492115
C62	-2.6458188394	0.0046962209	-1.2048562263
H63	-1.2237197827	1.3498219567	-2.1356851212
H64	-2.4303262249	2.1654084403	-1.1419044213
H65	-1.3704773028	1.2454600980	0.9129370460
H66	0.6463796165	0.0037231139	-0.9975452864
H67	-1.3711859503	-1.2389759312	0.9115759169
H68	-1.2245940469	-1.3403646505	-2.1371228770
H69	-2.4315625993	-2.1561837242	-1.1440273743
H70	-3.2997267534	0.0044299061	-0.3203519006
H71	-3.3105569362	0.0053519701	-2.0781542732
C72	0.0919315764	-2.5169691762	-0.0096513670
H73	-0.4881161621	-3.4374364012	0.1226611181
H74	0.5875301873	-2.5993676753	-0.9842016214
C75	0.0933607421	2.5236607973	-0.0069741536
H76	-0.4861058177	3.4443386765	0.1264420287
H77	0.5889351872	2.6068425754	-0.9814842655
C78	5.3880393363	-0.0023258443	4.9283908311
C79	4.0520669120	-0.0007677470	5.3341936898
C80	3.0229227916	0.0004382372	4.3880441830
C81	3.2468198336	0.0002869288	2.9898328596
C82	4.6162159917	-0.0013299748	2.6286225624
C83	5.6618866332	-0.0026209211	3.5606974742
H84	6.1933695372	-0.0032792969	5.6584128982
H85	3.8070278878	-0.0005468480	6.3952899964
H86	1.9982491937	0.0013801735	4.7591866380
H87	4.8974903350	-0.0017097160	1.5743265058
H88	6.6934579247	-0.0039327625	3.2118756777

TS2-24 *anti*

H1	2.1933883981	-0.2916673698	3.0285122073
Ir2	1.6731196574	-0.0323927853	1.5180412778
P3	1.3496210952	-2.3668004532	1.3420291806
P4	1.3748822552	2.3116364637	1.4303759415
C5	2.7289681609	-3.5065147620	0.6117962872
C6	3.3175817937	-2.7394202569	-0.5959328344
H7	3.6159430329	-1.7224197821	-0.3358414368
H8	2.6034914629	-2.6675628105	-1.4224863650
H9	4.1975982779	-3.2765867014	-0.9716697023
C10	2.2268282798	-4.8696940664	0.0876011176
H11	3.0562430591	-5.3745885492	-0.4249895075
H12	1.4151725566	-4.7693650396	-0.6384815785
H13	1.8889189523	-5.5333882178	0.8839784602
C14	3.8430434113	-3.7540178064	1.6475421349
H15	3.5082424804	-4.4125149292	2.4543398029
H16	4.2138481429	-2.8274544071	2.0913223467
H17	4.6902209763	-4.2519766153	1.1581779420
C18	0.5967057755	-3.1622354694	2.9278694297
C19	0.3134034743	-4.6724767447	2.8179820698
H20	1.2314461293	-5.2659775940	2.8247354418
H21	-0.2586409075	-4.9322127867	1.9216902395

H22	-0.2800076407	-4.9893591008	3.6856407727
C23	1.5147931528	-2.9155019809	4.1433564798
H24	1.0523238217	-3.3573148081	5.0356256627
H25	1.6504820837	-1.8483448320	4.3325234049
H26	2.5034811292	-3.3659108771	4.0298858838
C27	-0.7379527453	-2.4278555610	3.1876690603
H28	-1.1315325539	-2.7339076885	4.1654147827
H29	-1.4967725050	-2.6721988938	2.4379534976
H30	-0.5937543195	-1.3430424924	3.1957282805
C31	2.8719677091	3.4283071878	0.9404927327
C32	3.6884075777	2.6112284529	-0.0866889015
H33	4.5467259156	3.2095945659	-0.4196617712
H34	3.1015509149	2.3566103852	-0.9755462273
H35	4.0628903422	1.6830485031	0.3478909027
C36	3.7764009607	3.7266303631	2.1516932311
H37	4.0792513106	2.8159328231	2.6728823907
H38	3.2963226087	4.3961140616	2.8717264370
H39	4.6875070613	4.2302821124	1.8035117546
C40	2.4662439869	4.7603038608	0.2723664116
H41	1.9196477099	5.4257940221	0.9417244113
H42	1.8604198657	4.6105515724	-0.6255772764
H43	3.3770012597	5.2885164692	-0.0386616864
C44	0.4137424003	3.1086512569	2.9023924020
C45	1.1102158755	2.7614927376	4.2319152089
H46	0.5549283146	3.2205510284	5.0605717412
H47	2.1404579229	3.1196354108	4.2846006045
H48	1.1144603370	1.6798680645	4.3860409275
C49	0.2408976716	4.6357409512	2.7985803004
H50	-0.2066861727	4.9434347013	1.8481800680
H51	1.1836739392	5.1734311079	2.9261593786
H52	-0.4332675806	4.9723275822	3.5972643664
C53	-0.9910871671	2.4682495816	2.9457828332
H54	-0.9307391239	1.3760394093	2.9293994746
H55	-1.6233671436	2.7974134145	2.1164914876
H56	-1.4898706125	2.7702092251	3.8757433940
C57	-1.4210308881	1.3058081891	-1.5275105997
C58	-0.6290762457	1.2832836952	-0.2029467474
C59	0.2224616524	-0.0024250313	-0.0630464030
C60	-0.7351125796	-1.2189344147	-0.1300350907
C61	-1.5601810253	-1.2289367172	-1.4372730320
C62	-2.3389352422	0.0791663740	-1.6401311807
H63	-0.7123037862	1.3169687868	-2.3696335478
H64	-2.0163772669	2.2263761991	-1.6066197791
H65	-1.3727002200	1.2680764864	0.6043201102
H66	0.8804510252	-0.0519320134	-0.9574507600
H67	-1.4438680283	-1.1188916902	0.7048180830
H68	-0.8713531917	-1.3766632471	-2.2831778839
H69	-2.2532452668	-2.0821069946	-1.4471537595
H70	-3.1274187718	0.1512261805	-0.8767452372
H71	-2.8505754037	0.0696647103	-2.6111226759
C72	0.0202699855	-2.5407133630	0.0415443660
H73	-0.6521121754	-3.3814174091	0.2480307880
H74	0.5316750181	-2.7775537084	-0.8981936788
C75	0.2340873416	2.5356597241	-0.0323202137
H76	-0.3635821930	3.4520004671	0.0411155746
H77	0.8752443414	2.6472446770	-0.9154500349
C78	6.2258682759	0.3189180749	3.8091803621
C79	5.1321643406	0.6382439402	4.6108814002
C80	3.8297436395	0.5060844472	4.1179578190
C81	3.5595374093	0.0513837098	2.8106089698
C82	4.6869352848	-0.2950810511	2.0338632087
C83	5.9899929890	-0.1492850604	2.5143471056
H84	7.2399328737	0.4241201292	4.1848913505
H85	5.2839036833	0.9937602905	5.6276446327
H86	3.0050518592	0.7601807514	4.7770459373
H87	4.5508388410	-0.6590565114	1.0230842336
H88	6.8261471342	-0.4062871034	1.8675285378

2-syn

H1	2.6531774036	0.0095712521	0.8745802826
Ir2	1.3724115770	0.0051240834	1.7157595069

P3	1.1730984097	-2.3327699182	1.4476834033
P4	1.1655160720	2.3442734271	1.4494772283
C5	2.7406665574	-3.4313101431	1.2486436561
C6	3.6057764379	-2.7846922206	0.1414224552
H7	3.8954908016	-1.7650893382	0.4075880809
H8	3.1110817897	-2.7556224484	-0.8332445496
H9	4.5219692336	-3.3758265857	0.0231563317
C10	2.3898299751	-4.8736920319	0.8325016475
H11	3.3158838476	-5.4285450013	0.6334518358
H12	1.7835172752	-4.9128912867	-0.0780600550
H13	1.8575127602	-5.4105651546	1.6227944214
C14	3.5981703098	-3.4570743650	2.5311656564
H15	3.1464431010	-4.0462608438	3.3295066257
H16	3.7897196584	-2.4533719757	2.9153533931
H17	4.5650864173	-3.9205803638	2.2964187099
C18	-0.0668806019	-3.1074009608	2.7117842557
C19	-0.8984613965	-4.2727236388	2.1405201257
H20	-0.2805484570	-5.0939083304	1.7685973522
H21	-1.5605646951	-3.9511759595	1.3314534004
H22	-1.5360229096	-4.6779118028	2.9369466999
C23	0.6500071140	-3.5760642204	3.9937524928
H24	-0.1041924915	-3.7904934366	4.7622016939
H25	1.3227213405	-2.8123980174	4.3933489817
H26	1.2157171650	-4.4978441768	3.8355660588
C27	-1.0342008247	-1.9698505921	3.1097659746
H28	-1.7683085272	-2.3496632016	3.8327045658
H29	-1.5877174718	-1.5705337869	2.2559607975
H30	-0.4970762348	-1.1489004218	3.5940304129
C31	2.7333945993	3.4418353190	1.2387436025
C32	3.5810383214	2.8085470551	0.1105532016
H33	4.5037200802	3.3905393107	-0.0023850097
H34	3.0779182927	2.8085099569	-0.8602120008
H35	3.8600061425	1.7795305266	0.3507701371
C36	3.6090343465	3.4470237096	2.5092127118
H37	3.8093394790	2.4369805261	2.8714951421
H38	3.1665594876	4.0181570034	3.3255797625
H39	4.5706740551	3.9189519198	2.2697100725
C40	2.3830433323	4.8910769664	0.8465990532
H41	1.8718661291	5.4227492708	1.6540684517
H42	1.7576397357	4.9444321150	-0.0502992034
H43	3.3083853810	5.4420348632	0.6337028998
C44	-0.0622571731	3.1262797810	2.7207440776
C45	0.6659383577	3.5908284800	3.9979548047
H46	-0.0819674853	3.8100691010	4.7711954609
H47	1.2360928867	4.5092361614	3.8362478727
H48	1.3368694393	2.8232443033	4.3931548208
C49	-0.8897603859	4.2971103973	2.1542614988
H50	-1.5594583485	3.9796836190	1.3498259472
H51	-0.2693536951	5.1142066383	1.7779823435
H52	-1.5193683671	4.7066172547	2.9547939954
C53	-1.0354010994	1.9961193485	3.1249341356
H54	-0.5022185260	1.1720671048	3.6079892475
H55	-1.5953987020	1.5993855260	2.2740634729
H56	-1.7637619752	2.3827458057	3.8500641553
C57	-1.1455256495	1.2744973583	-1.8983613803
C58	-0.5474199658	1.2672801713	-0.4750835767
C59	0.2986066293	0.0036661481	-0.2150519679
C60	-0.5352380068	-1.2667952657	-0.4837682792
C61	-1.1281275068	-1.2746253540	-1.9094757675
C62	-1.9532297122	-0.0044505177	-2.1755293552
H63	-0.3237460505	1.3513005097	-2.6264881750
H64	-1.7821272772	2.1577133282	-2.0507966240
H65	-1.3990506294	1.2656213518	0.2246981182
H66	1.0945535242	0.0097075123	-0.9837957320
H67	-1.3903197000	-1.2747537142	0.2117183899
H68	-0.3035000917	-1.3338918830	-2.6359193731
H69	-1.7520280889	-2.1655046508	-2.0692663506
H70	-2.8441088279	-0.0135363569	-1.5299933441
H71	-2.3236814663	-0.0017029131	-3.2085219322
C72	0.3200139883	-2.5123748718	-0.2117425659
H73	-0.2472042177	-3.4441306427	-0.2933192760

H74	1.1107852408	-2.5588719797	-0.9688323549
C75	0.2992532189	2.5197665135	-0.2041395505
H76	-0.2756944306	3.4472462203	-0.2806134801
H77	1.0849662401	2.5741399055	-0.9658299677
C78	4.0826425243	0.0037278565	5.9657892901
C79	4.6951031483	-0.0000401208	4.7109285604
C80	3.9234679887	0.0004544035	3.5444031288
C81	2.5059581239	0.0048251446	3.5457269155
C82	1.9326642982	0.0087819293	4.8403683176
C83	2.6888621243	0.0081746838	6.0194554597
H84	4.6760654795	0.0032610609	6.8764608169
H85	5.7816054285	-0.0035135024	4.6369405817
H86	4.4505887160	-0.0027007319	2.5907063816
H87	0.8475207202	0.0125990574	4.9525901624
H88	2.1817580601	0.0113374126	6.9831847308

TS2-24 *syn*

H1	2.7894061261	-0.3606342648	2.3589179466
Ir2	1.3611406920	-0.0379083643	1.7072195258
P3	1.1789717554	-2.3632382194	1.3846819619
P4	1.2499576402	2.3317813357	1.4451405113
C5	2.8540275852	-3.2615345377	1.0449849322
C6	3.5389528899	-2.4877738823	-0.1073033190
H7	3.5703135115	-1.4137605832	0.1000572110
H8	3.0392506204	-2.6347684604	-1.0691882154
H9	4.5678484540	-2.8526517472	-0.2179350556
C10	2.6936300314	-4.7359079061	0.6276417109
H11	3.6628797459	-5.1248094449	0.2887291930
H12	1.9852180828	-4.8619319537	-0.1975298791
H13	2.3691549639	-5.3674415831	1.4593423543
C14	3.7934089556	-3.1802874703	2.2679249972
H15	3.4257127303	-3.7426652059	3.1271845739
H16	3.9616655684	-2.1498101834	2.5892906806
H17	4.7677802543	-3.6048204608	1.9933245611
C18	0.0904177102	-3.4069385226	2.6050968884
C19	-0.4908363957	-4.7012952988	1.9980212834
H20	0.2758312498	-5.3867524772	1.6324048320
H21	-1.1890722254	-4.5020310819	1.1807217441
H22	-1.0558636067	-5.2297982203	2.7770515784
C23	0.8891386717	-3.7835044054	3.8704223257
H24	0.1955413998	-4.1644083711	4.6312345704
H25	1.4215739147	-2.9342745649	4.3036193412
H26	1.6122032372	-4.5794840073	3.6720032972
C27	-1.1016747285	-2.5108603996	3.0134807891
H28	-1.6754475781	-3.0094302640	3.8055197172
H29	-1.7823553319	-2.3383589868	2.1750003790
H30	-0.7771395372	-1.5337533386	3.3777933394
C31	2.8513607027	3.2855115714	0.9364864349
C32	3.6936103314	2.2905017194	0.1076930612
H33	4.6142339663	2.7859131771	-0.2283044827
H34	3.1596657745	1.9414070222	-0.7824914729
H35	3.9607755249	1.4067453505	0.6924171522
C36	3.6743579100	3.7267397583	2.1628570320
H37	3.8272634369	2.9166036043	2.8776036072
H38	3.2085457121	4.5638712657	2.6901590916
H39	4.6618910288	4.0682649558	1.8264702940
C40	2.5817182909	4.5315878396	0.0642638473
H41	1.9723805255	5.2814455235	0.5720740856
H42	2.0960021841	4.2860143859	-0.8833931450
H43	3.5427526756	5.0025090012	-0.1802609519
C44	0.2938685006	3.3316333379	2.7904565021
C45	0.9643028125	3.1921427537	4.1721153708
H46	0.3494779089	3.7173756367	4.9149508273
H47	1.9619917480	3.6326257791	4.2080013001
H48	1.0448817001	2.1491760406	4.4788337331
C49	0.1295168601	4.8290425411	2.4652040154
H50	-0.3053707419	5.0053164570	1.4764023762
H51	1.0763201517	5.3723123626	2.5264798506
H52	-0.5478859086	5.2811442455	3.2016438928
C53	-1.1090029427	2.6905370189	2.8869234657
H54	-1.0419889532	1.6085295428	3.0402195480

H55	-1.7145481347	2.8774872089	1.9953979063
H56	-1.6427360454	3.1235873786	3.7424743592
C57	-1.3878072339	1.3087067551	-1.6571907796
C58	-0.6901299182	1.2752894965	-0.2795170534
C59	0.1749818767	0.0094152196	-0.0930110917
C60	-0.6497469942	-1.2659380251	-0.3813281215
C61	-1.3309437448	-1.2227348568	-1.7645420224
C62	-2.2017973797	0.0329259847	-1.9166928494
H63	-0.6202232660	1.4116595687	-2.4396484124
H64	-2.0356418771	2.1930484149	-1.7365109769
H65	-1.4807619575	1.2667624776	0.4843322191
H66	0.9597566104	0.0530214170	-0.8742124280
H67	-1.4541412577	-1.3299411196	0.3665570708
H68	-0.5544936887	-1.2251654633	-2.5448780867
H69	-1.9378040057	-2.1252569365	-1.9231324408
H70	-3.0362995827	-0.0172852849	-1.2016695639
H71	-2.6536559487	0.0664406309	-2.9162223752
C72	0.2541674554	-2.4973629902	-0.2348754625
H73	-0.2928087775	-3.4389764650	-0.3333342621
H74	0.9980563494	-2.4785043935	-1.0385558448
C75	0.1821962643	2.5200362176	-0.0802880046
H76	-0.3988242291	3.4488423697	-0.0684443246
H77	0.8671085952	2.5904200521	-0.9332028282
C78	3.3380510786	0.3010606622	6.3986826123
C79	4.1590399159	0.6918254260	5.3433786162
C80	3.7187420037	0.5580612923	4.0234009627
C81	2.4468042552	0.0406556148	3.6982568707
C82	1.6516290463	-0.3759705388	4.7879383824
C83	2.0806803067	-0.2347918302	6.1094618286
H84	3.6723110693	0.4025141373	7.4277910529
H85	5.1486762797	1.0979664428	5.5410872414
H86	4.3935073352	0.8485832751	3.2211900581
H87	0.6642934802	-0.7834078856	4.6072501533
H88	1.4242932365	-0.5459787584	6.9195410891

24

H1	-0.8906358847	-0.0049204421	1.3736044635
Ir2	1.6727678617	0.0000925251	1.0616322014
P3	1.4849355451	2.3091805478	0.9838761064
P4	1.4865763307	-2.3087685372	0.9811630736
C5	1.4665835158	3.2619621620	2.6573966646
C6	0.6535142513	2.3778084960	3.6305378164
H7	1.0755889328	1.3706280548	3.6931190722
H8	-0.3929031016	2.2779905735	3.3236546729
H9	0.6609272645	2.8350541091	4.6288142485
C10	0.8042727281	4.6535103051	2.5994874149
H11	0.7408277472	5.0640249271	3.6158101175
H12	-0.2163175151	4.6090160591	2.2072655558
H13	1.3662076039	5.3677995861	1.9957183013
C14	2.8969284673	3.3913241534	3.2160422830
H15	3.5068246281	4.0955582482	2.6427711011
H16	3.4112176614	2.4243417089	3.2347919100
H17	2.8547547884	3.7642815475	4.2475757288
C18	2.6360888261	3.1897820185	-0.2784329869
C19	2.5404695997	4.7250357932	-0.3074707850
H20	2.9430550479	5.1836250294	0.5998223369
H21	1.5115223229	5.0754992162	-0.4416713371
H22	3.1280180577	5.1108265598	-1.1512352388
C23	4.0909906694	2.7592968960	0.0048746625
H24	4.7512924928	3.1679240537	-0.7712037401
H25	4.1779972540	1.6678529485	-0.0125693220
H26	4.4590475260	3.1163774247	0.9698155686
C27	2.2551048609	2.6417733779	-1.6711857458
H28	2.9898503078	2.9901788533	-2.4082924503
H29	1.2713591569	2.9912227225	-1.9999228113
H30	2.2564591142	1.5469750088	-1.6740914625
C31	1.4570556210	-3.2583164324	2.6555624003
C32	0.6338571380	-2.3757485345	3.6212332965
H33	0.6360108732	-2.8314634968	4.6202320076
H34	-0.4107726754	-2.2807686763	3.3067685030
H35	1.0515941763	-1.3668453634	3.6850180403

C36	2.8836225273	-3.3834250683	3.2247668752
H37	3.3955874504	-2.4152737334	3.2458061354
H38	3.4990588674	-4.0870584807	2.6566729435
H39	2.8351470309	-3.7552865020	4.2564079188
C40	0.8002040121	-4.6523516252	2.5938386075
H41	1.3704741080	-5.3654221441	1.9963743311
H42	-0.2168307130	-4.6125609977	2.1919879773
H43	0.7288810416	-5.0617577944	3.6100851133
C44	2.6458118711	-3.1899041947	-0.2739019273
C45	4.0992488458	-2.7609168557	0.0190007490
H46	4.7640024051	-3.1682947727	-0.7539652299
H47	4.4612581700	-3.1203687585	0.9853525290
H48	4.1872171004	-1.6694985705	0.0044001247
C49	2.5495610774	-4.7251747955	-0.3047623190
H50	1.5211780599	-5.0751861455	-0.4443916013
H51	2.9474489416	-5.1846642685	0.6041699434
H52	3.1412434288	-5.1105519118	-1.1458685014
C53	2.2736410089	-2.6402530875	-1.6685208733
H54	2.2747851621	-1.5454337314	-1.6701830055
H55	1.2920527678	-2.9893828673	-2.0039962488
H56	3.0131239362	-2.9877294252	-2.4013330126
C57	-2.2064242849	-1.2713473341	-0.6943165576
C58	-0.6941156644	-1.2611156977	-0.3808294111
C59	-0.3024244470	-0.0013115532	0.4338882832
C60	-0.6983675887	1.2616031385	-0.3736254312
C61	-2.2110447732	1.2688575178	-0.6852964098
C62	-2.6379374952	0.0005982228	-1.4398025865
H63	-2.7658291662	-1.3509720238	0.2499952590
H64	-2.4646496447	-2.1607896396	-1.2856488516
H65	-0.1627039462	-1.2035089144	-1.3408243501
H66	-0.1684619398	1.2101633501	-1.3347390324
H67	-2.7699348086	1.3393676806	0.2600427489
H68	-2.4732771884	2.1614791376	-1.2700112520
H69	-2.1790414970	0.0050419499	-2.4390275671
H70	-3.7237912621	-0.0009105705	-1.5990502305
C71	-0.2557131981	2.5485143966	0.3430724105
H72	-0.3525023162	3.4313205875	-0.2995388036
H73	-0.9035022736	2.7175638305	1.2114454998
C74	-0.2498381399	-2.5506743794	0.3300662024
H75	-0.3397609752	-3.4298604554	-0.3184604570
H76	-0.9015771238	-2.7279049006	1.1937854212

16

C1	-0.0322269795	0.0005399502	0.0699854009
Ir2	1.5330792108	0.0006397439	1.1322819207
P3	1.4463208724	2.3378263495	0.9815311774
P4	1.4484121051	-2.3366806079	0.9800112843
C5	1.3753898921	3.3668889261	2.5993615039
C6	0.5939545057	2.5031477137	3.6159645856
H7	1.1108151532	1.5615276023	3.8144605466
H8	-0.4190486144	2.2670975407	3.2727741560
H9	0.5027302962	3.0575033740	4.5590668867
C10	0.6477512307	4.7183224447	2.4383374005
H11	0.6098186509	5.2160103694	3.4156842241
H12	-0.3857914428	4.6013216237	2.0986514231
H13	1.1557670864	5.3952701426	1.7489619392
C14	2.7899991422	3.6072085306	3.1642508920
H15	3.3631108040	4.3163420297	2.5599274341
H16	3.3538993293	2.6744927637	3.2468174388
H17	2.6999940922	4.0397483242	4.1689110882
C18	2.6758363032	3.1049416097	-0.2756798951
C19	2.5723382729	4.6360961039	-0.4040936908
H20	2.9100768640	5.1519487044	0.4980789950
H21	1.5542198992	4.9702186660	-0.6315826992
H22	3.2154020619	4.9716584600	-1.2281463340
C23	4.1184674134	2.7071818819	0.1009968665
H24	4.8007850595	3.0505475007	-0.6875267177
H25	4.2207365056	1.6241430859	0.2015878858
H26	4.4466513384	3.1555599628	1.0404218070
C27	2.3556328301	2.4746105966	-1.6492465148
H28	3.1191872960	2.7881915759	-2.3717755262

H29	1.3856380016	2.7988020432	-2.0400971266
H30	2.3635399701	1.3820777506	-1.5962222940
C31	1.3724849876	-3.3655314553	2.5980293915
C32	0.5880462526	-2.5014081420	3.6119857440
H33	0.4945199130	-3.0551605839	4.5552126354
H34	-0.4240853561	-2.2659202460	3.2658772112
H35	1.1040481571	-1.5594438075	3.8111676099
C36	2.7856074779	-3.6054422377	3.1669164447
H37	3.3494331856	-2.6726778276	3.2498860415
H38	3.3601338752	-4.3153093970	2.5647396633
H39	2.6929480060	-4.0368779740	4.1718018878
C40	0.6456426950	-4.7172244346	2.4355647363
H41	1.1556768899	-5.3942511911	1.7477350260
H42	-0.3870542492	-4.6006529625	2.0932001362
H43	0.6053962274	-5.2144995586	3.4130226745
C44	2.6820793838	-3.1043058047	-0.2729798385
C45	4.1237310317	-2.7075818411	0.1083700662
H46	4.8082829741	-3.0510328134	-0.6781576842
H47	4.4486496122	-3.1567506696	1.0485969597
H48	4.2263971561	-1.6246836978	0.2099486496
C49	2.5781832558	-4.6354600156	-0.4013537674
H50	1.5607004323	-4.9692386141	-0.6321766499
H51	2.9125824109	-5.1511421471	0.5022185793
H52	3.2239467912	-4.9716193114	-1.2230286535
C53	2.3664139130	-2.4736997419	-1.6474650346
H54	2.3751952268	-1.3811773172	-1.5943437940
H55	1.3972096424	-2.7968425097	-2.0411026092
H56	3.1317162574	-2.7879898384	-2.3678224883
C57	-2.2358966901	-1.2468040150	-0.5500069377
C58	-0.6894312993	-1.2789334287	-0.4892293451
C59	-0.6929791456	1.2802341954	-0.4845551085
C60	-2.2393663626	1.2443237677	-0.5434521685
C61	-2.7673896659	-0.0001237486	-1.2547171150
H62	-2.6290251859	-1.2753049793	0.4766005854
H63	-2.5919862026	-2.1592351785	-1.0449993990
H64	-0.3368839519	-1.3220978799	-1.5343093588
H65	-0.3424287605	1.3271949186	-1.5301397360
H66	-2.6315043443	1.2661613613	0.4836955166
H67	-2.5985380437	2.1583459751	-1.0332649976
H68	-2.4457084184	0.0031950686	-2.3062314772
H69	-3.8642133721	-0.0017574361	-1.2624540593
C70	-0.2412245349	2.5642718042	0.2372471681
H71	-0.3118885684	3.4410924100	-0.4155318097
H72	-0.9202600908	2.7439268436	1.0774509994
C73	-0.2363639074	-2.5639115432	0.2299976212
H74	-0.3035231889	-3.4392468114	-0.4250804730
H75	-0.9171523385	-2.7468078721	1.0678115814
Cl76	3.5178435597	0.0006502446	2.6068652588

3. NMR Spectra of 3, 5, 6, 7, and 21

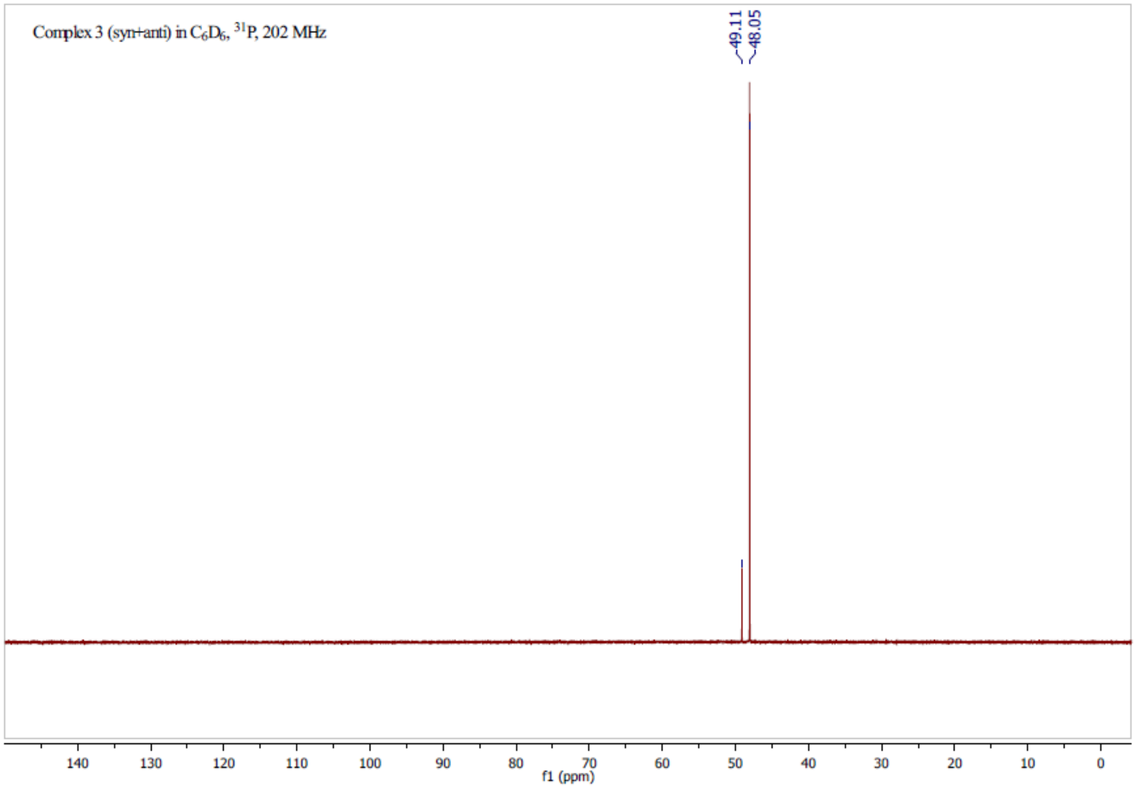

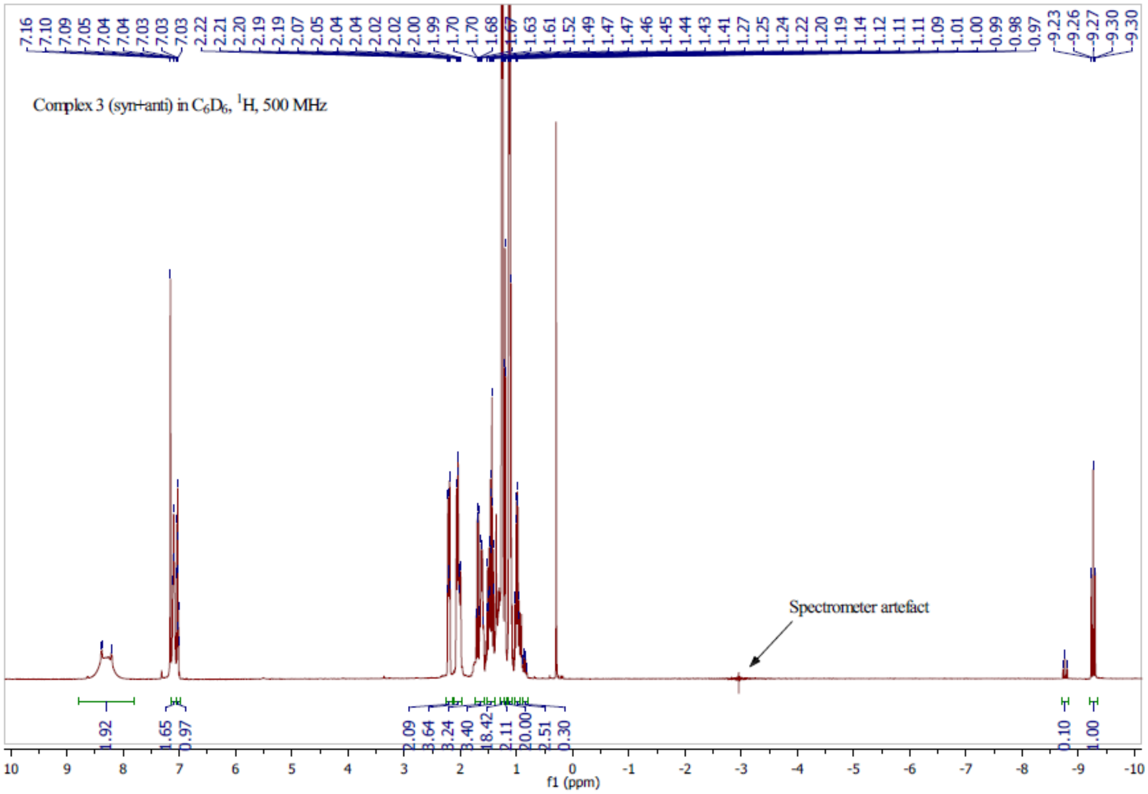

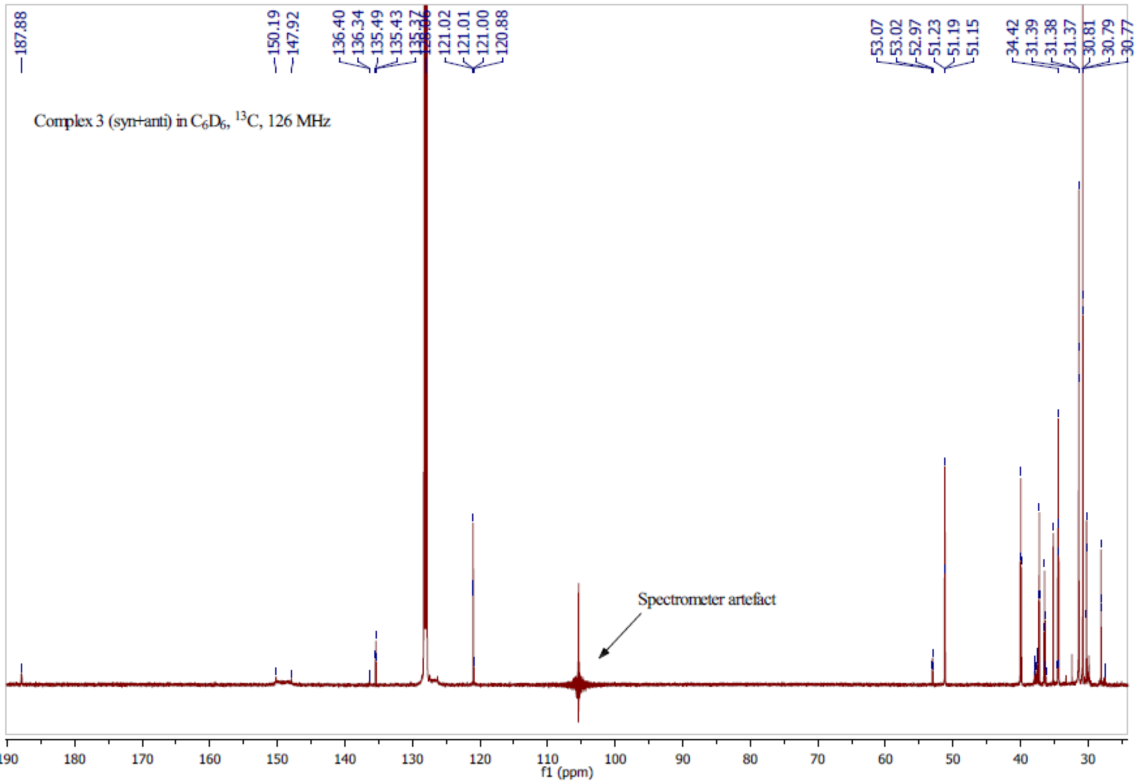
187.88
150.19
147.92
136.40
136.34
135.49
135.43
135.37
121.02
121.01
121.00
120.88
53.07
53.02
52.97
51.23
51.19
51.15
34.42
31.39
31.38
31.37
30.81
30.79
30.77
Complex 3 (syn+anti) in C_6D_6, ^{13}C, 126 MHz
Spectrometer artefact
190 180 170 160 150 140 130 120 110 100 90 80 70 60 50 40 30
f1 (ppm)

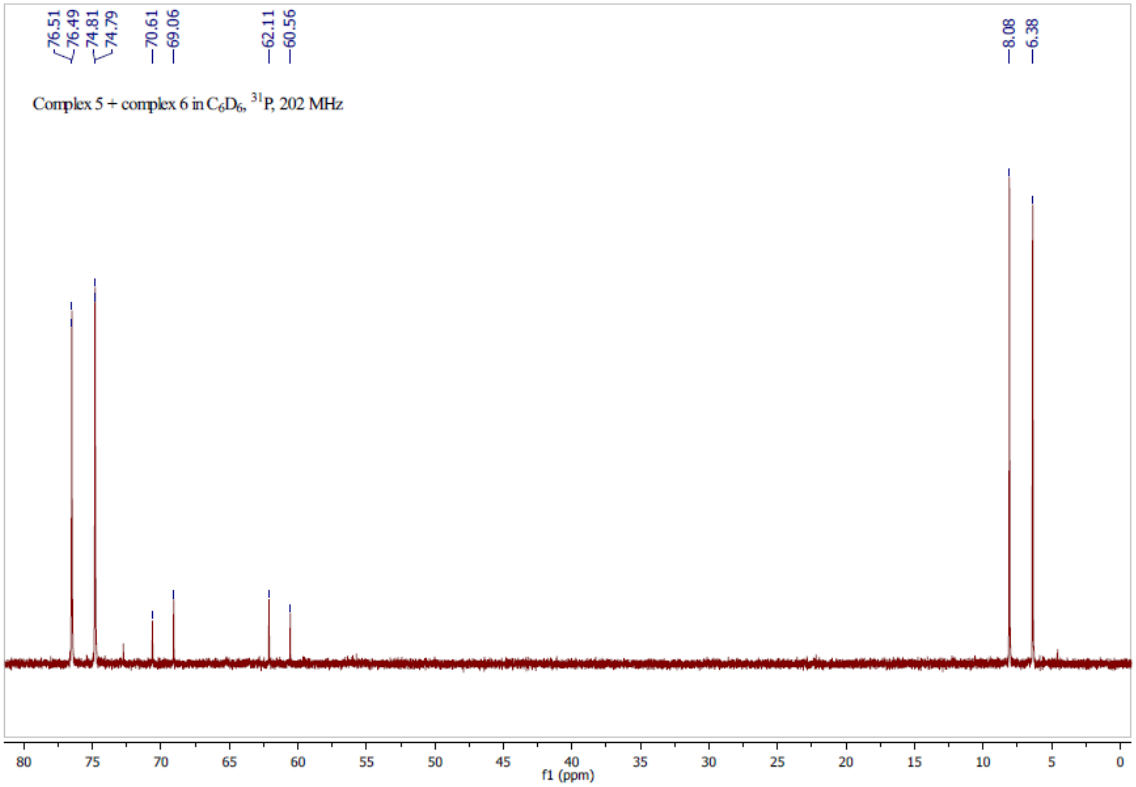
76.51
76.49
74.81
74.79
70.61
69.06
62.11
60.56
8.08
6.38
Complex 5 + complex 6 in C_6D_6, ^{31}P, 202 MHz
80 75 70 65 60 55 50 45 40 35 30 25 20 15 10 5 0
f1 (ppm)

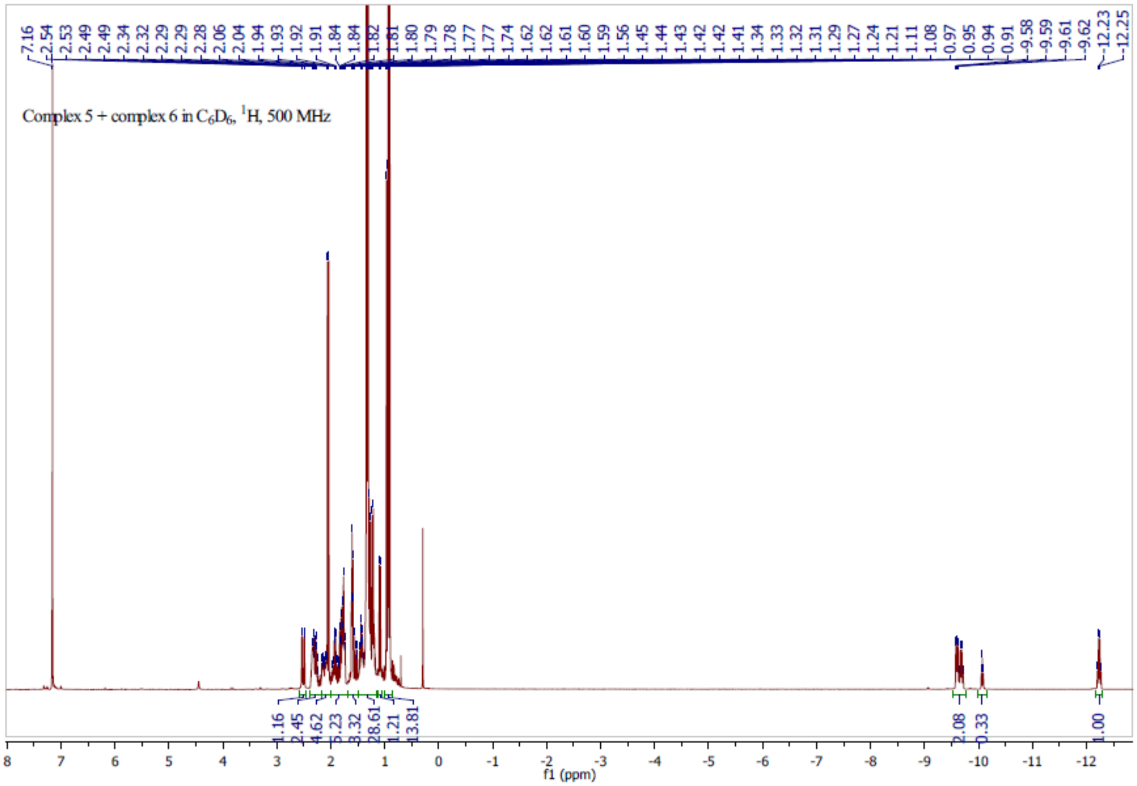
7.16
2.54
2.53
2.49
2.49
2.34
2.32
2.29
2.29
2.28
2.06
2.04
1.94
1.93
1.92
1.91
1.84
1.84
1.82
1.81
1.80
1.79
1.78
1.77
1.77
1.74
1.62
1.62
1.61
1.60
1.59
1.56
1.45
1.44
1.43
1.42
1.42
1.41
1.34
1.33
1.32
1.31
1.29
1.27
1.24
1.21
1.11
1.08
0.97
0.95
0.94
0.91
-9.58
-9.59
-9.61
-9.62
-12.23
-12.25
Complex 5 + complex 6 in C_6D_6, ^{1}H, 500 MHz
1.16
2.45
4.62
5.23
3.32
28.61
1.21
13.81
2.08
0.33
1.00
f1 (ppm)
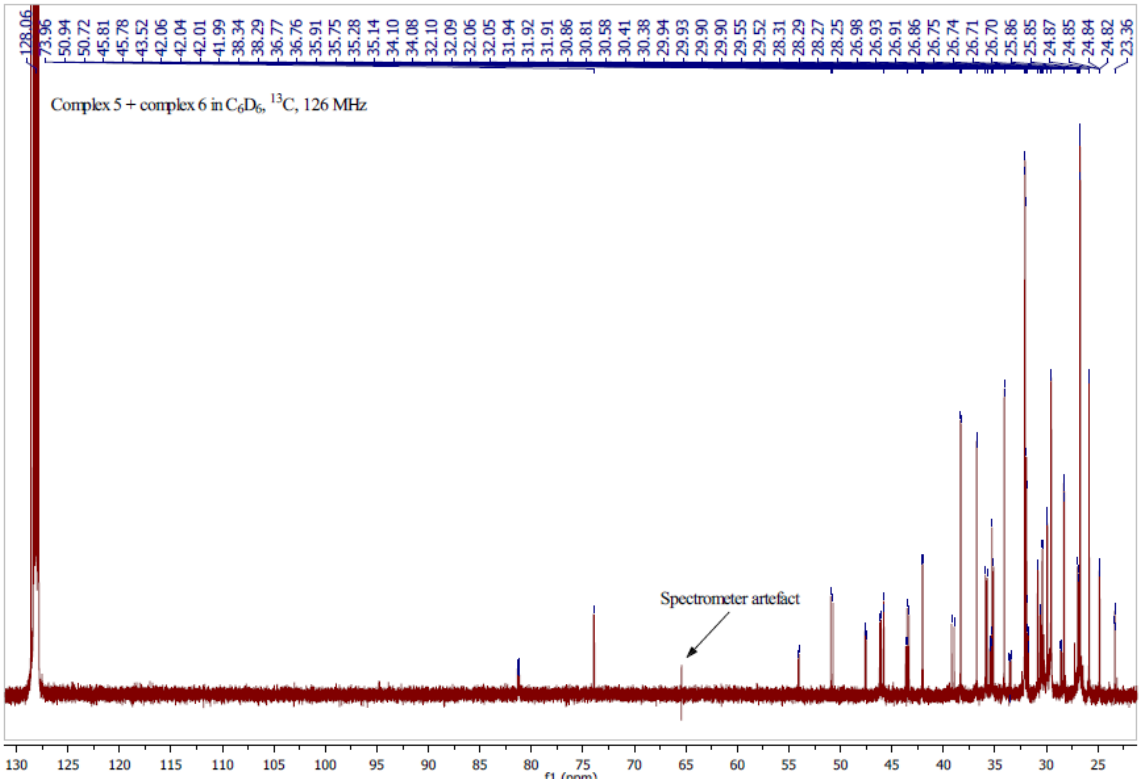
128.06
73.96
50.94
50.72
45.81
45.78
43.52
42.06
42.04
42.01
41.99
38.34
38.29
36.77
36.76
35.91
35.75
35.28
35.14
34.10
34.08
32.10
32.09
32.06
32.05
31.94
31.92
31.91
30.86
30.81
30.58
30.41
30.38
29.94
29.93
29.90
29.55
29.52
28.31
28.29
28.27
28.25
26.98
26.93
26.91
26.86
26.75
26.74
26.71
26.70
25.86
25.85
24.87
24.85
24.84
24.82
23.36
Complex 5 + complex 6 in C_6D_6, ^{13}C, 126 MHz
Spectrometer artefact
f1 (ppm)

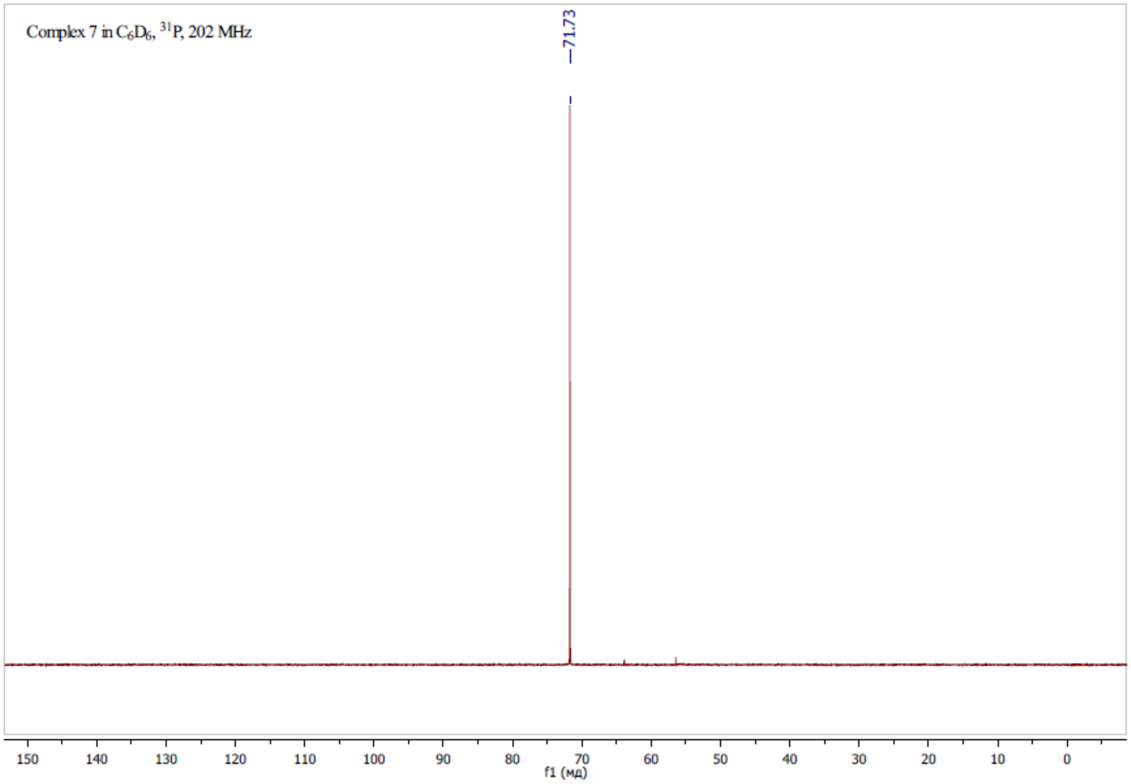
Complex 7 in C_6D_6, ^{31}P, 202 MHz
71.73
f1 (мд)

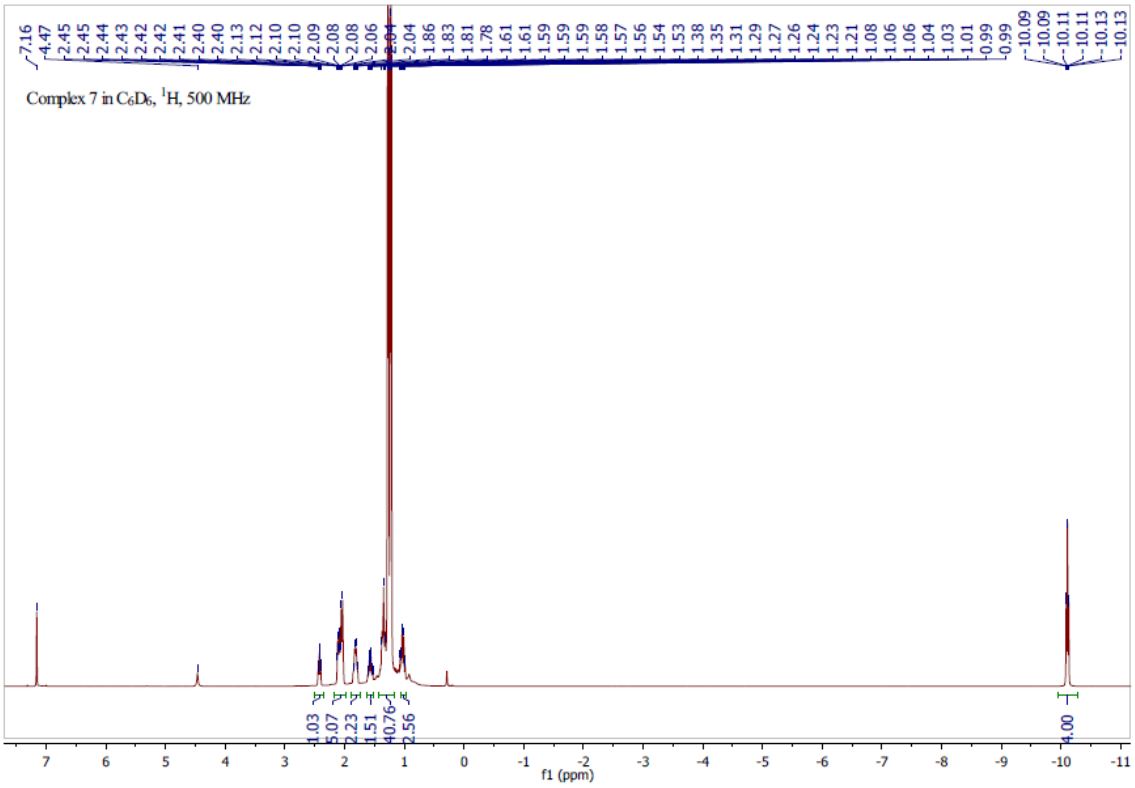
Complex 7 in C_6D_6, ^{1}H, 500 MHz
f1 (ppm)

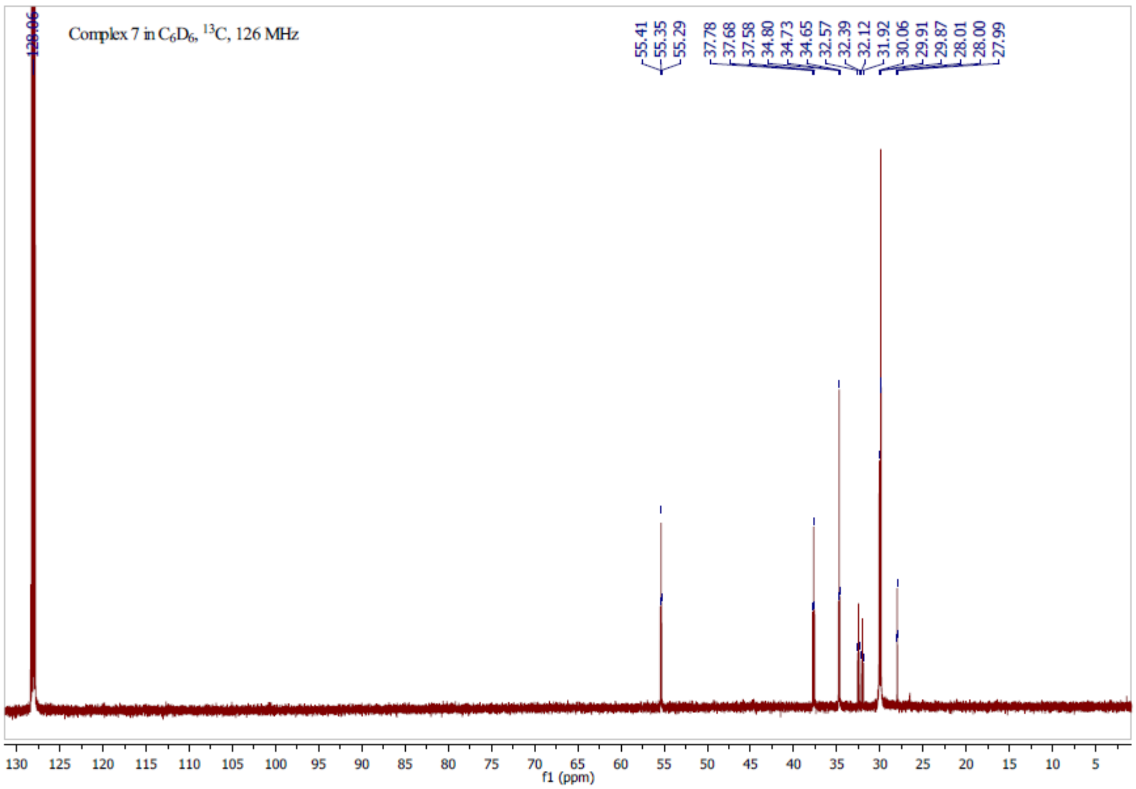
Complex 7 in C_6D_6, ^{13}C, 126 MHz
f1 (ppm)

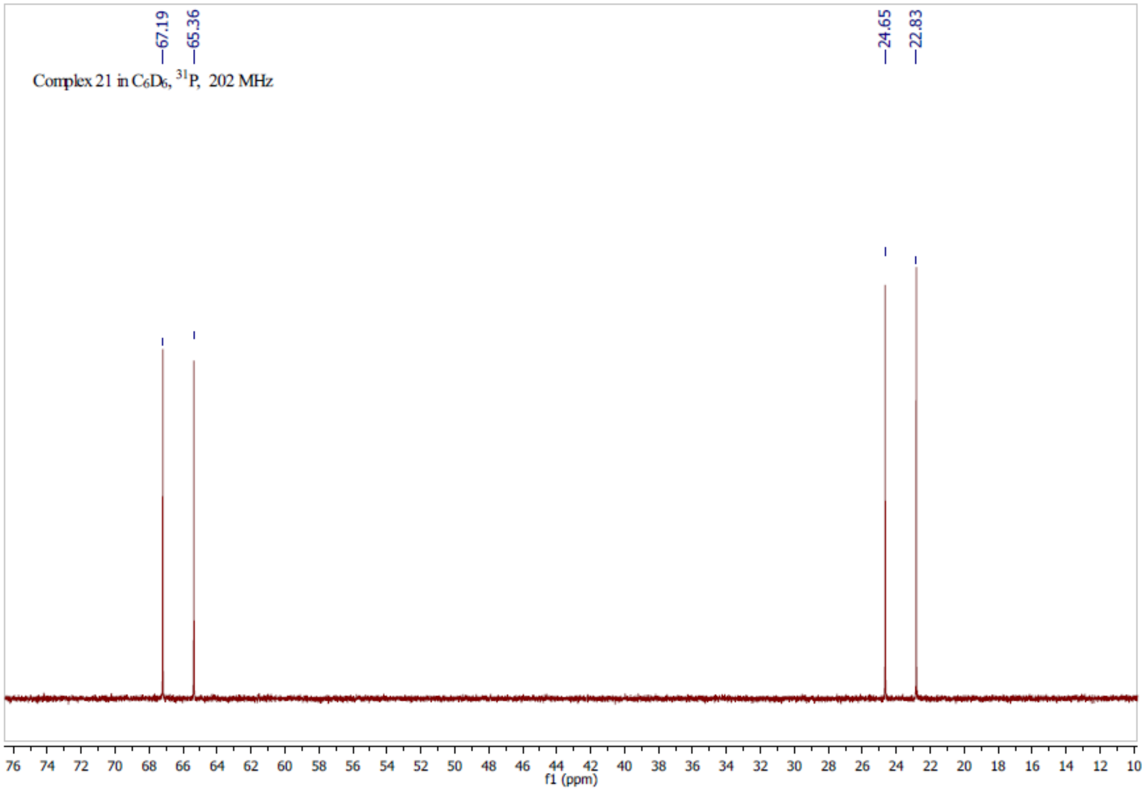
Complex 21 in C_6D_6, ^{31}P, 202 MHz
67.19
65.36
24.65
22.83
f1 (ppm)

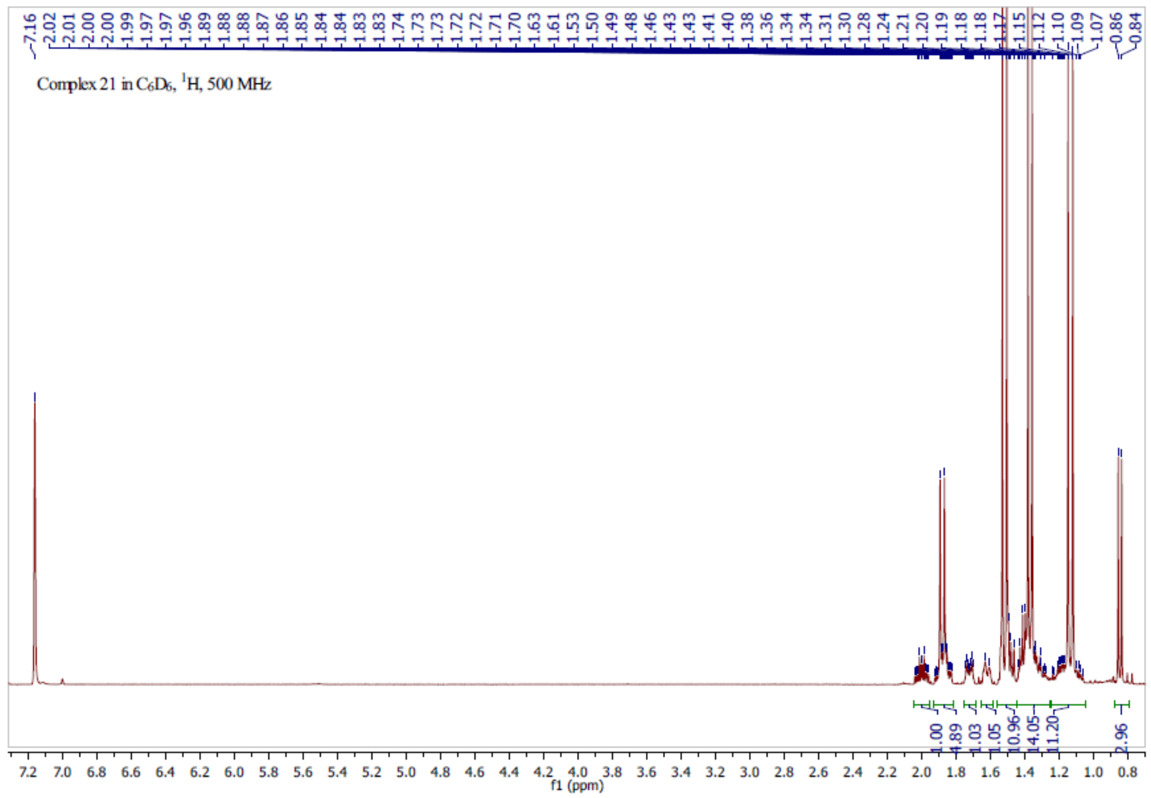
Complex 21 in C_6D_6, ^1H, 500 MHz
f1 (ppm)

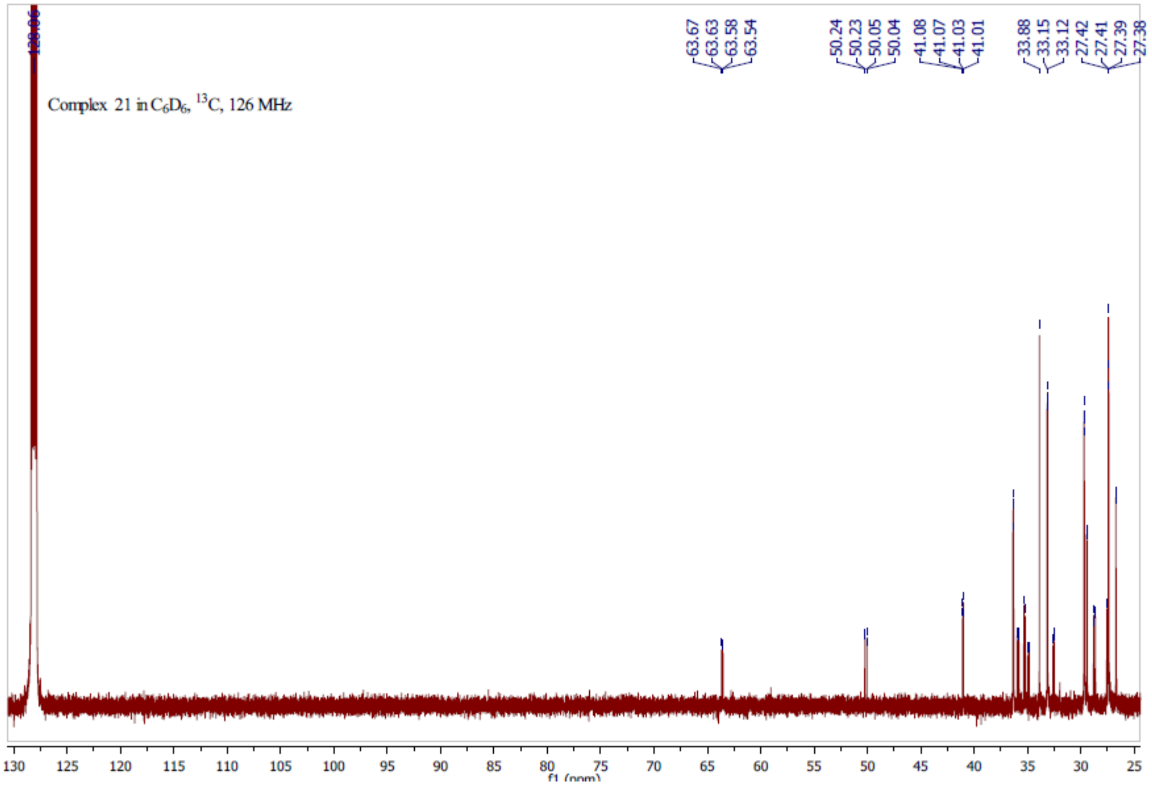
Complex 21 in C_6D_6, ^{13}C, 126 MHz
f1 (ppm)

4. References

1. C. J. Moulton, B. L. Shaw, *J. Chem. Soc., Dalton Trans.* 1976, 1020.
2. Crysalis CCD, Oxford Diffraction Ltd. Abingdon, Oxfordshire, UK, 2005.
3. G. M. Sheldrick, SHELXTL, 5.1, Program for structure solution and least squares refinement, University of Göttingen, Göttingen, Germany, 1998.
4. Jaguar, version 7.6, Schrödinger, LLC, New York, NY, 2009; www.schrodinger.com.
5. A. D. Becke, *J. Chem. Phys,* 1993, **98**, 5648.
6. C. Lee, W. Yang and R. G. Parr, *Phys. Rev. B.* 1988, **37**, 785.
7. Y. Zhao and D. G. Truhlar, *Theor. Chem. Acc.* 2008, **120**, 215.
8. Y. Zhao and D. G. Truhlar, *J. Chem. Phys.* 2006, **125**, 194101.
9. S. Grimme, J. Antony, S. Ehrlich, H. Krieg, *J. Chem. Phys.* 2010, **132**,154104.
10. J. M. L. Martin and A. Sundermann, *J. Chem. Phys.* 2001, **114**, 3408.
11. B. Marten, *et al. J. Phys. Chem.* 1996, **100**, 11775.
12. B. B. Averkiev and D. G. Truhlar, *Catal. Sci. Technol.* 2011, **1**, 1526.
13. G. Frenkingand and N. Fröhlich, *Chem. Rev.* 2000, **100**, 717.
14. G. Frenking, M. Sola and S. F. Vyboishchikov, *J. Organomet. Chem.* **2005**, *690*, 6178.
